# The Size of Activating and Inhibitory Killer Ig-like Receptor Nanoclusters Is Controlled by the Transmembrane Sequence and Affects Signaling

**DOI:** 10.1016/j.celrep.2016.04.075

**Published:** 2016-05-19

**Authors:** Anna Oszmiana, David J. Williamson, Shaun-Paul Cordoba, David J. Morgan, Philippa R. Kennedy, Kevin Stacey, Daniel M. Davis

**Affiliations:** 1 Manchester Collaborative Centre for Inflammation Research, University of Manchester, 46 Grafton Street, Manchester M13 9NT, UK; 2 Division of Cell and Molecular Biology, Sir Alexander Fleming Building, Imperial College London, London SW7 2AZ, UK

## Abstract

Super-resolution microscopy has revealed that immune cell receptors are organized in nanoscale clusters at cell surfaces and immune synapses. However, mechanisms and functions for this nanoscale organization remain unclear. Here, we used super-resolution microscopy to compare the surface organization of paired killer Ig-like receptors (KIR), KIR2DL1 and KIR2DS1, on human primary natural killer cells and cell lines. Activating KIR2DS1 assembled in clusters two-fold larger than its inhibitory counterpart KIR2DL1. Site-directed mutagenesis established that the size of nanoclusters is controlled by transmembrane amino acid 233, a lysine in KIR2DS1. Super-resolution microscopy also revealed two ways in which the nanoscale clustering of KIR affects signaling. First, KIR2DS1 and DAP12 nanoclusters are juxtaposed in the resting cell state but coalesce upon receptor ligation. Second, quantitative super-resolution microscopy revealed that phosphorylation of the kinase ZAP-70 or phosphatase SHP-1 is favored in larger KIR nanoclusters. Thus, the size of KIR nanoclusters depends on the transmembrane sequence and affects downstream signaling.

## Highlights

• Activating and inhibitory NK cell receptors have a distinct nanoscale organization
• The transmembrane sequence of KIR controls their nanoscale organization
• Nanoclusters of KIR2DS1 and its adaptor are juxtaposed but mix upon activation
• Phosphorylation of ZAP-70 or SHP-1 is favored in larger receptor nanoclusters

## Graphical Abstract

**Figure:**
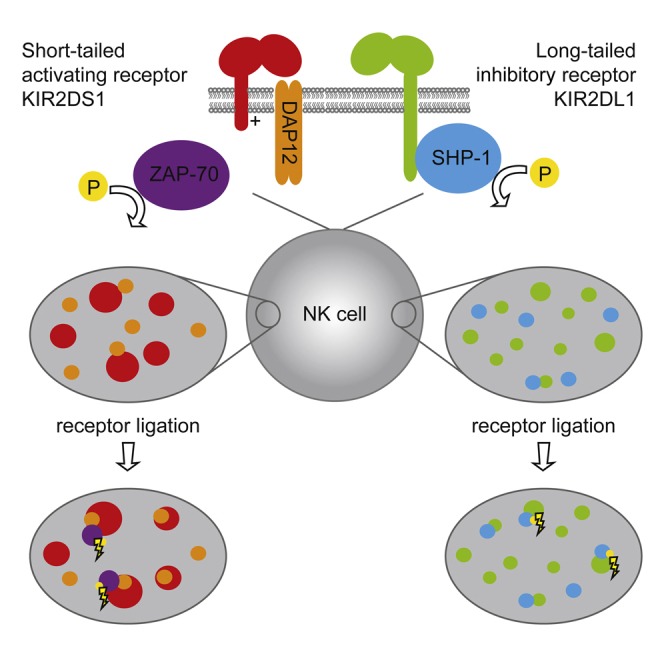

## Teaser

Oszmiana et al. use different super-resolution microscopy techniques to compare the nanoscale organization of paired activating and inhibitory receptors at the human NK cell surface. They show that the size of receptor nanoclusters is controlled by the transmembrane sequence and establish that nanocluster size affects the strength of receptor signaling.

## Introduction

Natural killer (NK) cells are part of our defense against cancer and viral infections and are of medical importance in cancer immunotherapy and bone marrow transplantation (Vivier et al., 2012, Davis, 2014, Della Chiesa et al., 2014, Foley et al., 2014). Their activity depends on the balance of signals from germ-line encoded activating and inhibitory receptors. Activating receptors include NKG2D, which recognizes stress-inducible tumor ligands such as MICA, and the Fc receptor CD16, which mediates antibody-dependent cellular cytotoxicity. Inhibitory receptors that bind self-major histocompatibility complex class I proteins protect healthy cells from NK cell attack and include killer immunoglobulin (Ig)-like receptors (KIR). Interestingly, the KIR family also includes activating receptors, which share ligand specificity with their inhibitory counterparts due to structural homology of extracellular domains (Ivarsson et al., 2014, Biassoni et al., 1997). One example of such a pairing are receptors KIR2DL1 and KIR2DS1, which bind to human leukocyte antigen (HLA) proteins from the C2 group (Stewart et al., 2005, Sivori et al., 2011). KIR3DS1, in combination with its HLA ligand, is associated with delayed progression to AIDS and protection against hepatitis C infection (Khakoo et al., 2004, Alter et al., 2007, Alter et al., 2011, Carr et al., 2007, Alter et al., 2011). Also, *KIR2DS1* in the telomeric region of the *KIR B* haplotype was shown to have a protective effect against complications in pregnancy (Xiong et al., 2013, Hiby et al., 2010).

Functional divergence of KIR2DL1 and KIR2DS1 is conferred by differences in transmembrane and intracellular sequences. The longer cytoplasmic tail of KIR2DL1 contains two immunoreceptor tyrosine-based inhibition motifs (ITIMs), which recruit the tyrosine phosphatase SHP-1 (Fry et al., 1996, Burshtyn et al., 1996) to block the membrane proximal activating signals (Stebbins et al., 2003). KIR2DS1 lacks ITIMs and instead associates with DNAX activation protein 12 (DAP12), an adaptor protein containing an immunoreceptor tyrosine-based activation motif (ITAM) (Lanier et al., 1998). Cytolysis, cytokine production, and cellular proliferation are triggered in NK cells expressing KIR2DS1, but not KIR2DL1, upon interaction with HLA-C2^+^ target cells (Sivori et al., 2011, Moretta et al., 1995, Mandelboim et al., 1998, Rose et al., 2009). In NK cells expressing both activating and inhibitory paired receptors, effector functions are often inhibited (Moretta et al., 1995, Valés-Gómez et al., 1998, Watzl et al., 2000).

The nanoscale organization of NK cell receptors changes with the state of activation of the cell. Specifically, clusters of KIR2DL1 become smaller upon ligation of activating receptor NKG2D, increasing the local density of inhibitory receptors (Pageon et al., 2013). In murine NK cells, fluorescence correlation spectroscopy revealed that confinement of activating receptors at the plasma membrane changes upon NK cell education (Guia et al., 2011). However, a major unknown is whether the nanometer-scale organization of NK cell receptors affects signaling. Here, we compare the nanometer-scale organization of activating and inhibitory KIR2DS1 and KIR2DL1 at the surface of NK cells. We report that these two receptors are organized differently, determined by their transmembrane sequences. Importantly, we also establish that the size of receptor nanoclusters affects signaling.

## Results

### Distinct Nanoscale Organization of KIR2DL1 and KIR2DS1 in NKL Cells

To compare the organization of inhibitory KIR2DL1 and activating KIR2DS1, the human cell line NKL was stably transduced to express each receptor fused to a hemagluttinin (HA) tag at the C terminus (NKL/KIR2DL1-HA and NKL/KIR2DS1-HA; Figure S1). Tagged receptors retained functionality, as ligation of KIR2DL1-HA inhibited the formation of a dense ring of peripheral F-actin at the contact interface, and the secretion of interferon (IFN)-γ, in cells activated via NKG2D (Figures S1D and S1G). In contrast, ligation of KIR2DS1-HA triggered the formation of peripheral actin rings, as well as IFN-γ secretion (Figures S1E and S1H).

**Figure 1:**
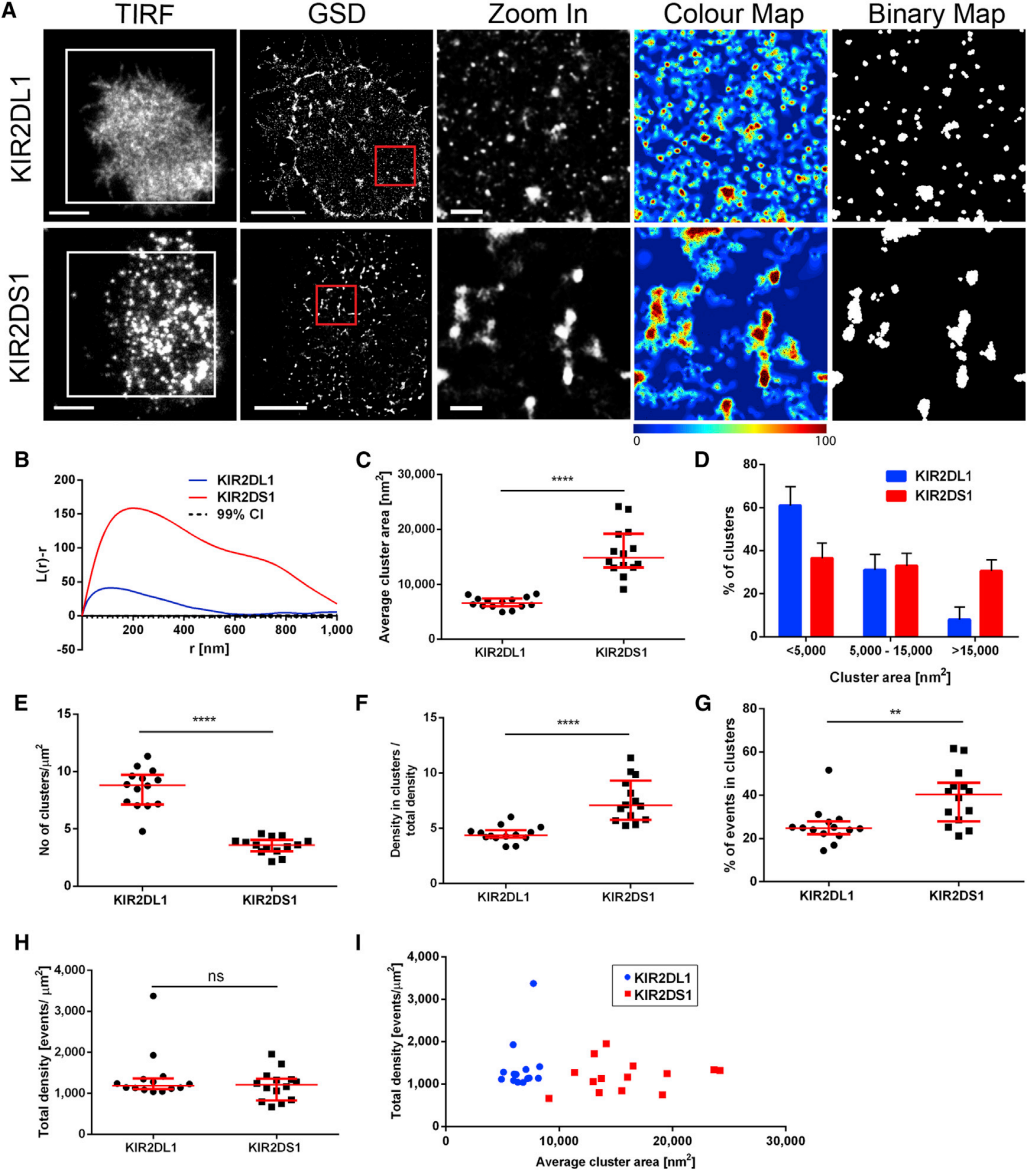
Distinct Organization of KIR2DL1 and KIR2DS1 at the Surface of NKL Depicted with GSD Microscopy (A–I) NKL/KIR2DL1-HA and NKL/KIR2DS1-HA cells on PLL-coated slides were stained with anti-KIR2DL/S1 mAb labeled with AF647. (A) Representative TIRF and GSD images of NKL/KIR2DL1-HA and NKL/KIR2DS1-HA cells. Scale bars represent 5 μm. The 3 × 3 μm regions (red boxes in GSD images) are magnified and shown with corresponding color cluster maps and binary maps (scale bars represent 500 nm). Colors correspond to the L(30) values. (B) Ripley’s *K* function of the events in the selected regions (red boxes) shown in (A). *L(r) − r* represents the degree of clustering at different spatial scales relative to simulated random distributions, indicated by the 99% confidence intervals (CI); r is the radial scale. (C–H) Quantitative analysis of KIR2DL1 and KIR2DS1 clustering in NKL/KIR2DL1-HA and NKL/KIR2DS1-HA cells: average cluster area (C), size distribution of clusters (D), clusters per square micrometer (E), ratio between density of events in clusters to average membrane density (F), percentage of events in clusters (G), and overall density of detected events (H). (I) Total density of events plotted against average cluster area measured in individual cells. (C) and (E)–(I) Each symbol represents the mean from several regions within one cell. Horizontal bars and errors represent the medians and interquartile range. (D) Bars and errors represent means and SD. Data are from 14 cells per receptor from two independent experiments. ns, non-significant. ^∗∗^p < 0.01, ^∗∗∗∗^p < 0.0001, Mann-Whitney test. See also Figures S1, S2, S3, S4, and S5.

The nanoscale organization of KIR2DL1 and KIR2DS1 at the cell surface was compared using ground state depletion microscopy followed by individual molecule return (GSDIM). For this, NKL/KIR2DL1-HA and NKL/KIR2DS1-HA cells were plated on poly-L-lysine-coated slides, fixed and stained with a directly labeled anti-KIR2DL/S1 monoclonal antibody (mAb) EB6 (Figure 1). Visual inspection of images (Figure 1A), as well as Ripley’s *K* function-based analysis (Ripley, 1977) (Figure 1B), showed that both receptors constitutively assembled in nanometer-scale clusters, but the degree and radial scale of clustering were larger for KIR2DS1. We then created quantitative maps of clustering based on univariate Getis and Franklin’s local point pattern analysis (Getis and Franklin, 1987, Williamson et al., 2011, Pageon et al., 2013). Randomized data with the same density of localizations were compared with the experimental data to establish an appropriate threshold for the identification of clusters. Quantifications of this type provide information only on the events detected and are sensitive to changes in parameters such as search radius. In addition, both the hardware and the methods of analysis for super-resolution microscopy data are being developed at a rapid rate. An explicit example of the impact of this is that we report a lower fraction of KIR2DL1 in clusters compared to our earlier research (Pageon et al., 2013). Thus, as in nearly all super-resolution microscopy reports, precise values are indicative rather than definitive and analysis is effective in revealing relative differences between different receptors or conditions.

Our analysis here showed that KIR2DS1 formed clusters of larger area (with a median of 14,800 nm^2^) compared to KIR2DL1 (median 6,600 nm^2^; Figure 1C). The majority of KIR2DL1 clusters (61% ± 9%) had an area ≤5,000 nm^2^ (which corresponds to diameter ≤80 nm), and only 8% ± 6% were bigger than 15,000 nm^2^ (diameter ≥138 nm). In contrast, 31% ± 5% of KIR2DS1 clusters were over 15,000 nm^2^ (Figure 1D). The density of clusters was higher for KIR2DL1 compared to KIR2DS1 (p < 0.0001, Mann-Whitney test; Figure 1E). In addition, clusters of KIR2DS1 were more tightly packed, 7.1-fold denser than the average density within the membrane (Figure 1F) and contained a higher fraction of total molecules (median 40%, Figure 1G), whereas KIR2DL1 clusters were 4.4-fold denser than the membrane overall and contained a lower proportion of molecules (median 25%).

The total density of detected events for both receptors was comparable (p = 0.6913; Figure 1H), indicating that differences in organization were not due to variable numbers of events detected. Also, cross-correlation of cluster size and overall molecular density showed that in individual cells expressing comparable levels of each of the two receptors, KIR2DS1 consistently formed bigger clusters than KIR2DL1 (Figure 1I). Together, these data indicate that nanoclusters of KIR2DS1 are bigger and more densely packed, while clusters of KIR2DL1 are more numerous and smaller.

Several control experiments were carried out as follows. First, because these measurements crucially depend on the efficiency of labeling, we stained the same cells with a directly labeled mAb targeting the HA tag. When visualized this way, clusters of KIR2DS1 were also bigger than clusters of KIR2DL1 (Figures S2A and S2B). To check if the organization of KIR was altered by contact with poly-L-lysine-coated slides, we also imaged cells that were fixed and stained in suspension prior to plating upon glass slides. Again, we observed bigger clusters for KIR2DS1 (Figures S2H and S2I). Finally, we imaged the endogenous interleukin-2 receptor subunit α (IL-2Rα), which assembled into nanoclusters with identical characteristics in both transfectants showing that the presence of KIR2DS1 or KIR2DL1 did not alter the nanoclustering of the cell surface in general (Figures S3A–S3G). Together, these experiments further establish the distinct nanoscale organization of KIR2DL1 and KIR2DS1 at the surface of transfected cell lines.

### Nanoclusters of KIR2DL1 and KIR2DS1 Are Spatially Segregated

Earlier research suggested a role for co-localization of activating and inhibitory receptors on a micrometer-scale in the integration of their signals (Köhler et al., 2010). To characterize the nanoscale organization of KIR2DL1 and KIR2DS1 when co-expressed, NKL cells were transduced to express the two receptors fused to either HA or FLAG peptide tags in both combinations, i.e., NKL/KIR2DL1-HA/KIR2DS1-FLAG as well as NKL/KIR2DL1-FLAG/KIR2DS1-HA, and confirmed as functional (Figures S4A–S4C). Cells were plated on poly-L-lysine-coated slides, fixed, stained with anti-HA mAb conjugated to Alexa Fluor 488 (AF488) and anti-FLAG mAb conjugated to AF532 for imaging by stimulated emission depletion (STED) microscopy. As a positive control for co-localization, the same protein, KIR2DS1-HA, was stained with two different mAb, one against the HA tag (labeled with AF488) and another mAb against the extracellular portion of KIR2DL/S1 (labeled with AF532). To quantify co-localization for KIR2DS1 and KIR2DL1, a Pearson correlation coefficient, which ranges between 1.0 (complete co-localization) and −1.0 (complete segregation), was calculated for each cell. The median correlation coefficient for KIR2DS1 and KIR2DL1 was −0.17 in NKL/KIR2DL1-FLAG/KIR2DS1-HA and −0.22 in NKL/KIR2DL1-HA/KIR2DS1-FLAG cells, as compared to 0.77 in the positive control (Figures S4D and S4E). Thus, KIR2DS1 and KIR2DL1 are constitutively localized within spatially segregated nanoclusters.

### Distinct Nanoclustering of KIR2DL1 and KIR2DS1 in Primary Human NK Clones

**Figure 2:**
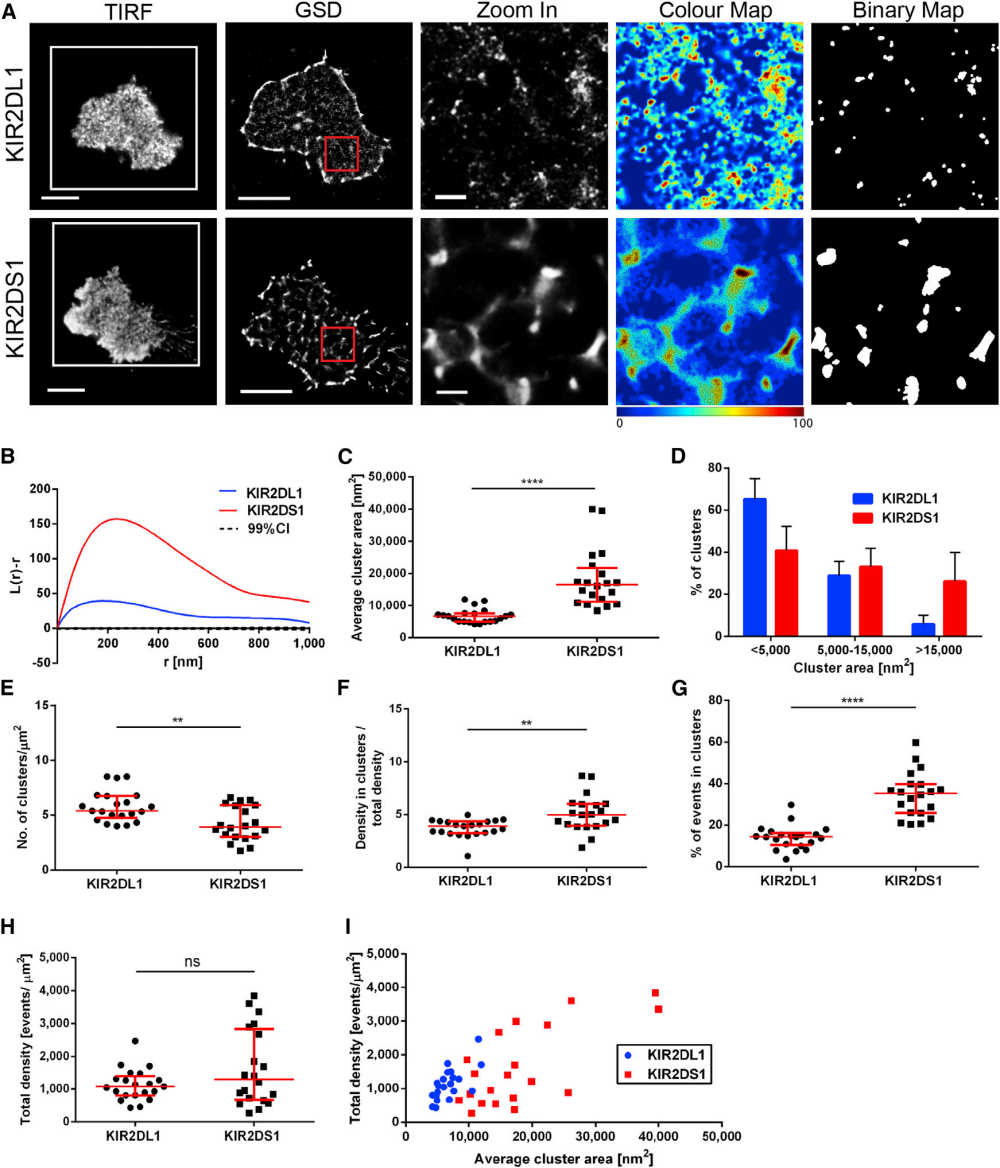
KIR2DL1 and KIR2DS1 Are Organized Differently at the Surface of Primary Human NK Clones (A) Representative TIRF and GSD images of 2DS1^−^/2DL1^+^ and 2DS1^+^/2DL1^−^ NK clones on PLL-coated slides stained with anti-KIR2DL/S1 mAb labeled with Atto 488. Scale bars represent 5 μm. The 3 × 3 μm regions (red boxes in GSD images) are magnified and shown with corresponding color cluster maps and binary maps (scale bars represent 500 nm). (B) Ripley’s *K* function of the events in the selected regions (red boxes) shown in (A). *L(r) − r* represents the degree of clustering at different spatial scales relative to simulated random distributions, indicated by the 99% confidence intervals (CI); r is the radial scale. (C–H) Quantitative analysis of KIR2DL1 and KIR2DS1 clustering in 2DS1^−^/2DL1^+^ and 2DS1^+^/2DL1^−^ NK clones, respectively: average cluster area (C), size distribution of clusters (D), number of clusters per square micrometer (E), ratio between density of events in clusters to overall membrane density (F), percentage of events in clusters (G), and overall density of detected events (H). (I) Total density of events plotted against average cluster area in individual cells for KIR2DL1 and KIR2DS1. Each symbol represents the mean from several regions within one cell (C and E–I). Horizontal bars and errors represent the medians and interquartile range. Bars and errors represent means and SD (D). Data are from 21 (KIR2DL1) and 20 (KIR2DS1) cells each from five clones derived from two donors. ns, non-significant. ^∗∗^p < 0.01, ^∗∗∗∗^p < 0.0001, Mann-Whitney test. See also Figure S5.

We next compared the nanoscale organization of KIR2DL1 and KIR2DS1 in human NK clones derived from peripheral blood. Clones expressing either KIR2DL1 or KIR2DS1 only, were identified using two mAb that compete for binding. mAb EB6 binds to NK cells expressing either one or both of the receptors, whereas mAb 143211 ligates only KIR2DL1 which allows discrimination between 2DS1^+^/2DL1^−^, 2DS1^−^/2DL1^+^ and 2DS1^+^/2DL1^+^ clones (Fauriat et al., 2010). Clones phenotyped as 2DS1^+^/2DL1^−^ and 2DS1^−^/2DL1^+^ were plated on poly-L-lysine-coated slides, fixed, stained with fluorescently labeled anti-KIR2DL/S1 mAb and imaged by GSDIM. As in transfectants, KIR2DL1 and KIR2DS1 assembled in nanoclusters with distinct characteristics (Figure 2A), demonstrated by *L(r) − r* function plots (Figure 2B) and quantitative analysis. Clusters of KIR2DS1 had significantly larger area (with a median of 16,600 nm^2^), than KIR2DL1 clusters (median 6,700 nm^2^; Figure 2C). In addition, only 6% ± 4% of KIR2DL1 clusters had area above 15,000 nm^2^, while such large clusters of KIR2DS1 were 4.5 times more frequent (26% ± 14%; Figure 2D). The density of KIR2DL1 clusters on primary cells was higher than for KIR2DS1 (p = 0.0036, Mann-Whitney test; Figure 2E). Clusters of KIR2DS1 were more tightly packed than KIR2DL1 (p = 0.0024; Figure 2F) and contained a higher fraction of the molecules (p < 0.0001, Figure 2G). The total density of receptors in 2DS1^+^/2DL1^−^ and 2DS1^−^/2DL1^+^ clones was similar (Figures 2H and 2I), implying that the differences in nanoscale organization of KIR2DL1 and KIR2DS1 cannot be accounted for by differences in expression level. Therefore, endogenously expressed KIR2DS1 and KIR2DL1 are distinctly organized at the surface of resting primary human NK cells.

Each super-resolution microscopy technique has caveats (Pageon et al., 2012). Therefore, we also compared the organization of KIR2DL1 and KIR2DS1 in primary cells and transfectants of NKL using STED microscopy. STED images of NKL/KIR2DL1-HA and NKL/KIR2DS1-HA cells stained with anti-HA mAb (Figures S5A and S5B) and 2DS1^+^/2DL1^−^ and 2DS1^−^/2DL1^+^ NK clones stained with anti-KIR2DL/S1 mAb (Figures S5C and S5D) were deconvolved and thresholded to create binary maps of nanoclusters. Measured this way, KIR2DS1 clusters had larger area than KIR2DL1 in both NKL (p < 0.0001; Mann-Whitney test; Figure S5B) and primary NK cells (p < 0.0001; Figure S5D).

### Nanoscale Clustering of KIR2DS1 and KIR2DL1 Is Controlled by the Transmembrane Sequence

**Figure 3:**
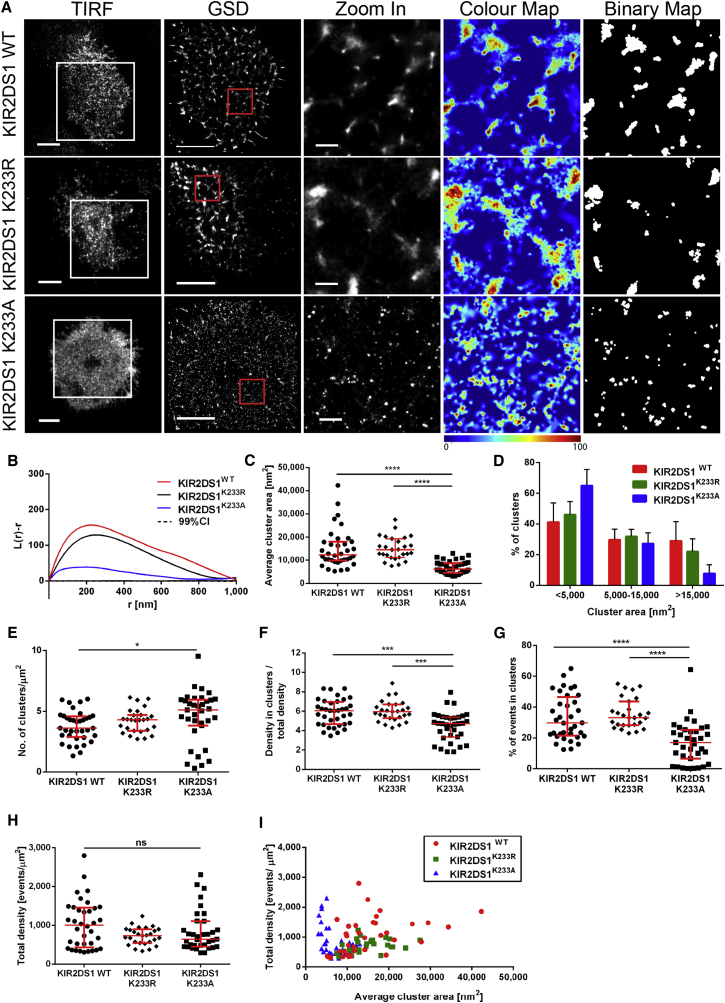
Substitution of the Transmembrane Lysine in KIR2DS1 Changes Its Nanometer-Scale Organization (A) Representative TIRF and GSD images of NKL/KIR2DS1-HA, NKL/KIR2DS1^K233R^, and NKL/KIR2DS1^K233A^ cells incubated on PLL-coated slides and stained with anti-HA mAb labeled with AF488. Scale bars represent 5 μm. The 3 × 3 μm regions (red boxes in GSD images) are magnified and shown with corresponding color maps and binary maps (scale bars represent 500 nm). (B) Ripley’s *K* function of the events in the selected regions (red boxes) shown in (A). (C–H) Analysis of wild-type KIR2DS1, KIR2DS1^K233R^, and KIR2DS1^K233A^ clustering: average cluster area (C), size distribution of clusters (D), clusters per square micrometer (E), ratio between density of events in clusters to average membrane density (F), percentage of events in clusters (G), and overall density of detected events (H). (I) Total density of detected events plotted against average cluster area in individual cells. (C and E–I) Each symbol represents the mean from several regions within one cell. Horizontal bars and errors represent the medians and interquartile range. (D) Bars and errors represent means and SD. Data are from 36 cells from five experiments (KIR2DS1^WT^), 27 cells from three experiments (KIR2DS1^K233R^), and 35 cells from four experiments (KIR2DS1^K233A^). ns, non-significant. ^∗^p < 0.05, ^∗∗∗^p < 0.001, ^∗∗∗∗^p < 0.0001, Kruskal-Wallis test by ranks with Dunn’s post-test. See also Figure S6.

KIR2DL1 and KIR2DS1 are highly homologous. The two alleles used here differ by only seven amino acids in their extracellular domain (representing 97% homology within this domain), with the bulk of the differences being in the transmembrane and cytoplasmic domains. Thus, it is not immediately obvious why these proteins organize differently at cell surfaces. To establish this, we tested the importance of the positively charged lysine at position 233 within transmembrane region of KIR2DS1, which facilitates interactions with DAP12 (Lanier et al., 1998, Bottino et al., 2000, Feng et al., 2006). For this, NKL was transduced to express mutant versions of KIR2DS1-HA where the transmembrane lysine had been substituted with neutral-charged alanine (KIR2DS1^K233A^) or positively charged arginine (KIR2DS1^K233R^). Cells were plated on poly-L-lysine-coated slides, fixed, stained with anti-HA mAb and imaged by GSDIM. Visual inspection (Figure 3A), *L(r) − r* plots (Figure 3B) and quantitative comparison of clusters establish that substitution of lysine 233 of KIR2DS1 with alanine, but not arginine, affected its organization.

The area of KIR2DS1^K233A^ clusters was significantly lower than wild-type KIR2DS1 (p < 0.0001, Kruskal-Wallis test by ranks with Dunn’s post-test; Figure 3C) and had a median of 6,100 nm^2^. The majority of KIR2DS1^K233A^ clusters (65% ± 11%) had area ≤5,000 nm^2^ (as opposed to 41% ± 13% for wild-type) and KIR2DS1^K233A^ clusters larger than 15,000 nm^2^ were rarely detected (7.5% ± 6% of all clusters, as opposed to 29% ± 13% for wild-type, Figure 3D). Density of KIR2DS1^K233A^ clusters was slightly increased compared to wild-type KIR2DS1 (p = 0.0145, Figure 3E). On the other hand, the size and density of KIR2DS1^K233R^ clusters was indistinguishable from wild-type receptor. Clusters of KIR2DS1^K233A^ were less densely packed and contained a lower proportion of molecules than clusters of wild-type KIR2DS1 and KIR2DS1^K233R^ (Figures 3F and 3G). The total density of events in cells imaged was similar (Figure 3H) and differences between cluster sizes of different receptors were consistent across a range of molecular densities (Figure 3I). Overall, KIR2DS1 in which the transmembrane lysine was replaced by alanine was organized similarly to KIR2DL1 across many parameters (*cf*. Figure 3 with Figure 1), while substituting the lysine for another charged amino acid, arginine, had little, if any, effect.

**Figure 4:**
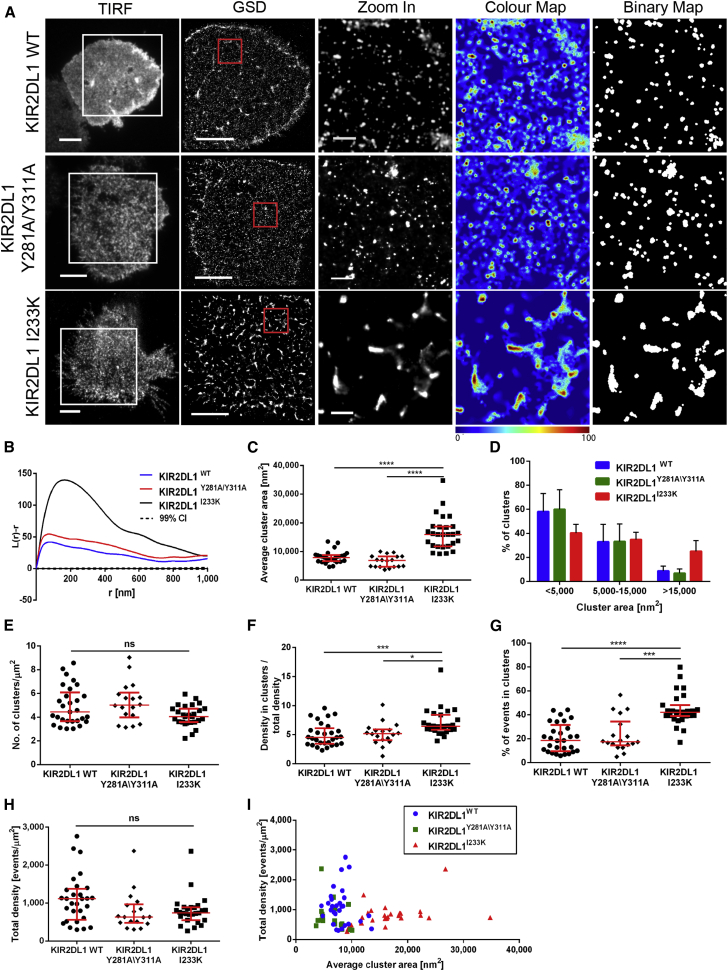
Substitution of the Transmembrane Isoleucine with Lysine in KIR2DL1 Changes Its Nanometer-Scale Organization (A) Representative TIRF and GSD images of NKL/KIR2DL1-HA, NKL/KIR2DL1^Y281A/Y311A^, and NKL/KIR2DL1^I233K^ cells incubated on PLL-coated slides, stained with anti-HA mAb labeled with AF488. Scale bars represent 5 μm. The 3 × 3 μm regions (red boxes in GSD images) are magnified and shown with corresponding color maps and binary maps (scale bars represent 500 nm). (B) Ripley’s *K* function of the events in the selected regions (red boxes) shown in (A). (C–H) Quantitative analysis of wild-type KIR2DL1, KIR2DL1^Y281A/Y311A^, and KIR2DL1^I233K^ clustering: average cluster area (C), size distribution of clusters (D), clusters per square micrometer (E), ratio between density of events in clusters to overall membrane density (F), percentage of events in clusters (G), and overall density of detected events (H). (I) Total density of detected events plotted against average cluster area in individual cells for wild-type KIR2DL1, KIR2DL1^Y281A/Y311A^, and KIR2DL1^I233K^. (C and E–I) Each symbol represents the mean from several regions within one cell. Horizontal bars and errors represent the medians and interquartile range. (D) Bars and errors represent means and SD. Data are from 29 cells from five experiments (KIR2DL1^WT^), 27 cells from four experiments (KIR2DL1^I233K^), and 18 cells from three experiments (KIR2DL1^Y281A/Y311A^). ns, non-significant. ^∗^p < 0.05, ^∗∗∗^p < 0.001, ^∗∗∗∗^p < 0.0001, Kruskal-Wallis test by ranks with Dunn’s post-test. See also Figure S6.

We next assessed whether, reciprocally, the insertion of a charged residue in the transmembrane sequence of inhibitory KIR2DL1 would influence its clustering. For this, NKL was transfected to express KIR2DL1 in which neutral isoleucine at position 233 was substituted with lysine (NKL/KIR2DL1^I233K^). To test whether tonic signaling by KIR could influence its clustering, we made a separate transfectant of NKL expressing KIR2DL1 in which the two ITIM tyrosines were substituted with alanine (NKL/KIR2DL1^Y281A/Y311A^). Introduction of lysine into the transmembrane domain of KIR2DL1 had a dramatic effect on the protein’s nanoscale organization (Figure 4A), as shown in plots of *L(r) − r* (Figure 4B) and quantitative analysis of clusters. In contrast, replacing the signaling tyrosines had little, if any, effect on clustering. The clusters of KIR2DL1^I233K^ were significantly larger than wild-type KIR2DL1 (p < 0.0001) and had a median area of 16,000 nm^2^ (Figure 4C). The distribution of KIR2DL1^I233K^ cluster sizes was skewed toward larger clusters (Figure 4D). We detected similar number of clusters per μm^2^ for KIR2DL1^I233K^ and wild-type KIR2DL1 (Figure 4E). The relative density of events in clusters and the proportion of events localized within clusters also increased for KIR2DL1^I233K^ (Figures 4F and 4G).

The mean levels of surface expression for mutated receptors differed to some extent from wild-type proteins (Figures S6A and S6C), but several lines of evidence determine that this did not account for the differences in nanoscale clustering. First, cells imaged had comparable levels of detected molecules (Figures 3H and 4H). Second, there was no obvious correlation between the total density of molecules detected and the average cluster size (Figure 3I and 4I). Third, to directly test for any effect of expression level, cells within the population that naturally express different densities of receptors were compared. For cells with low or high levels of expression, wild-type KIR2DS1 clusters were similar in size to KIR2DS1^K233R^ but consistently larger than clusters of KIR2DS1^K233A^ (Figure S6B). Similarly, KIR2DL1^I233K^ formed bigger and denser clusters than wild-type receptor or KIR2DL1^Y281A/Y311A^ in cells expressing the protein at different densities (Figure S6D).

### Association with DAP12 Does Not Account for the Differences in Clustering between KIR2DS1 and KIR2DL1

The lysine in the transmembrane sequence of KIR2DS1 facilitates its interaction with DAP12 (Lanier et al., 1998, Bottino et al., 2000, Feng et al., 2006). Thus, we next tested if an association with DAP12 affects the nanoscale clustering of KIR2DS1. For this, we stably transduced the human leukemic T cell line Jurkat E6.1, which lacks DAP12 (Lanier et al., 1998), to express KIR2DL1-HA or KIR2DS1-HA. Cells were plated on poly-L-lysine-coated slides, fixed, stained with anti-HA mAb and imaged by GSDIM (Figures S3H–S3N). The organization of KIR2DL1 and KIR2DS1 at the surface of Jurkat cells closely resembled that seen in NK cells (Figure S3H). Clusters of KIR2DS1 in Jurkat T cells were significantly larger than KIR2DL1 (p < 0.0001, Mann-Whitney test; Figure S3I) and were less abundant (p = 0.0001; Figure S3J). Also, a higher fraction of KIR2DS1 molecules was found within more densely packed clusters (Figures S3K and S3L). As in NK cells, the differences in KIR2DL1 and KIR2DS1 nanoclusters sizes were consistent across a range of molecular densities (Figures S3N and S6F). Thus, although it cannot be excluded that KIR2DS1 might associate with other adaptor molecules in Jurkat T cells, the presence of DAP12 is not required for KIR2DS1 to assemble in larger clusters.

### Nanoclusters of KIR2DS1 and DAP12 Mix upon Ligation of KIR2DS1

**Figure 5:**
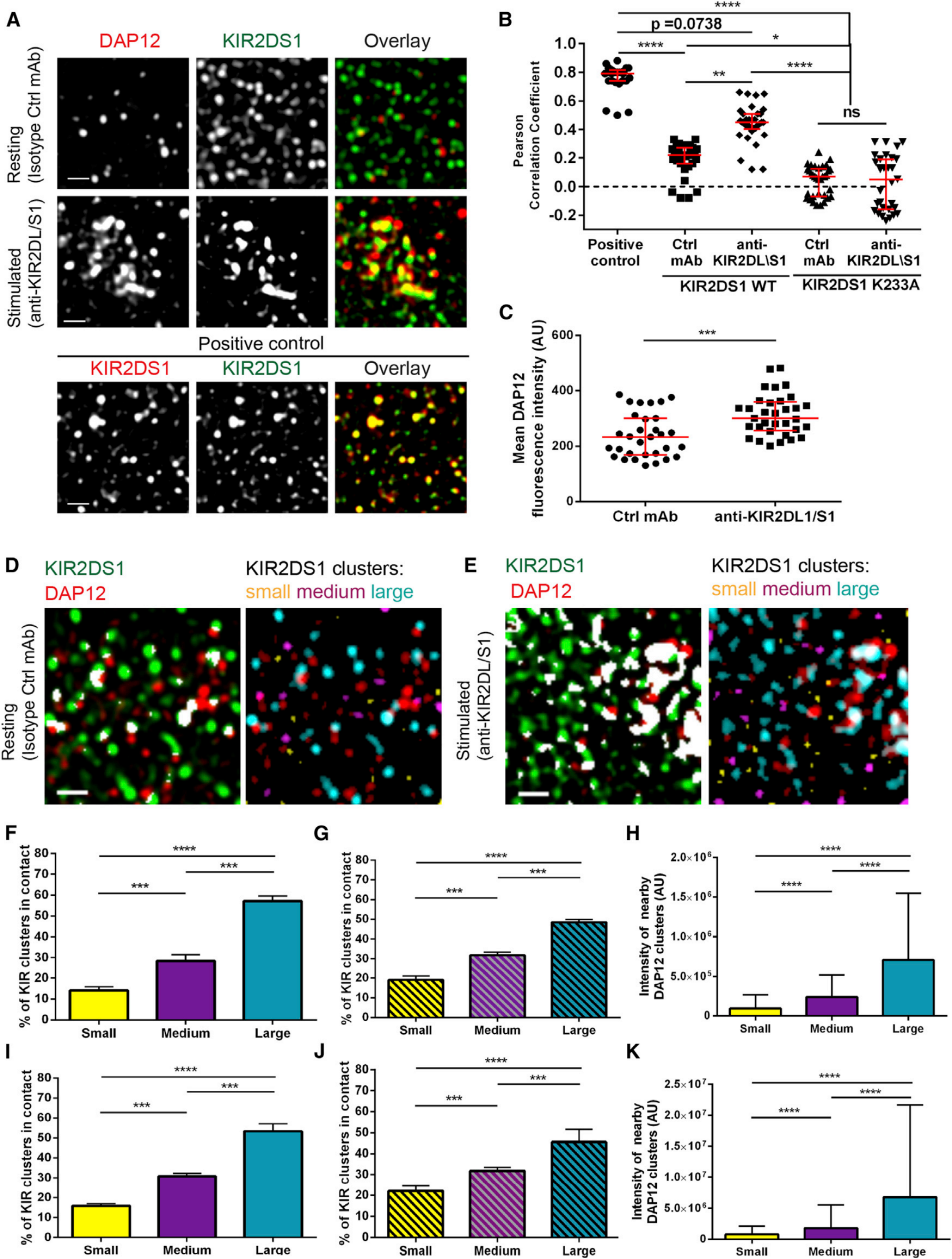
Interaction between Nanoclusters of KIR2DS1 and DAP12 (A) NKL/KIR2DS1-HA cells were incubated on isotype control-coated (top row) or anti-KIR2DL/S1 mAb-coated (middle row) slides, stained for KIR2DS1 (with an anti-HA mAb, AF488) and DAP12 (AF568), and imaged with STED. For a positive control (bottom row), NKL/KIR2DS1-HA was stained with anti-KIR2DL/S1 mAb (green; Atto488) and anti-HA mAb (red; AF568). Representative 3 × 3 μm regions from STED images of NKL/KIR2DS1-HA cells with channels overlaid and separated (overlapping green and red pixels are marked yellow). Scale bars represent 500 nm. (B) Pearson correlation coefficients for KIR2DS1 or KIR2DS1^K233A^ and DAP12 in cells on isotype control-coated and anti-KIR2DL/S1 mAb-coated slides, compared to positive control. (C) Mean DAP12-derived fluorescence intensity in NKL/KIR2DS1-HA cells on isotype control- and anti-KIR2DL/S1 mAb-coated slides. In (B) and (C), each symbol represents one cell. Horizontal bars and errors represent the medians and interquartile range. (D–K) Quantification of interaction between differently sized KIR2DS1 clusters and DAP12 clusters in NKL/KIR2DS1-HA cells incubated on isotype control-coated (D, F–H) and anti-KIR2DL/S1 mAb-coated slides (E, I–K). In (D) and (E), 3 × 3 μm regions of cell membrane with KIR2DS1 (green) and DAP12 (red) are shown with overlapping pixels marked in white (left) or with KIR2DS1 clusters color-coded according to their size (right). Each size bin corresponds to one-third of all KIR2DS1 clusters. (F and I) The fractions of all KIR2DS1 clusters associated with DAP12 that fall into each size group. (G and J) The fractions of all KIR2DS1 clusters associated with DAP12 that fall into each size group in simulated data where the positions of KIR2DS1 clusters were maintained and positions of DAP12 clusters were randomized within the boundaries of cell area. (H and K) Average intensity of DAP12 clusters found nearby KIR2DS1 clusters from specified size bins. (F–K) Bars and errors represent the medians and interquartile range. Data are from 25–34 cells from two independent experiments. AU, arbitrary units; ns, non-significant. ^∗^p < 0.05, ^∗∗^p < 0.01, ^∗∗∗^p < 0.001, ^∗∗∗∗^p < 0.0001. Kruskal-Wallis test by ranks with Dunn’s post-test (B), Mann-Whitney test (C), row-matched Friedman test with Dunn’s post-test (F–K). See also Figure S3 and Figure S7.

Having established that clustering of KIR2DS1 is independent of its association with DAP12, we next examined how these proteins are organized at the surface of NK cells with and without receptor ligation. For this, NKL/KIR2DS1-HA cells were plated on slides coated with anti-KIR2DL/S1 mAb or isotype-matched control mAb, and imaged by STED microscopy. Positive co-localization was indicated using KIR2DS1 labeled with anti-KIR2DS/L1 mAb conjugated to Atto 488 and anti-HA mAb followed by secondary Ab conjugated to AF568 (Figures 5A and 5B). Unexpectedly, on non-activating control-coated slides there was little direct overlap between clusters of KIR2DS1 and DAP12 (Figure 5A). This was reflected by a low Pearson correlation coefficient (median 0.22; Figure 5B). However, clusters of DAP12 tended to contact the edges of clusters of KIR2DS1 (Figure 5A). Importantly, when cells were stimulated on anti-KIR2DL/S1 mAb, the degree of mixing between nanoclusters of KIR2DS1 and DAP12 significantly increased. This was clearly seen in the overlay of images as well as by an increase in the Pearson correlation coefficient (median 0.45) (Figures 5A and 5B) and a decrease in the average distance between the KIR2DS1 and DAP12 cluster centroids, from a median of 240 nm in unstimulated cells to 153 nm upon ligation of KIR2DS1 (Figure S7A). Ligation of KIR2DS1 also led to the recruitment of DAP12 to the membrane at the contact site, as evidenced by an increase in DAP12 fluorescence intensity (Figure 5C), suggesting that at least some of the increased mixing between DAP12 and KIR2DS1 can come from increased levels of DAP12 at the membrane. Thus, ligation of KIR2DS1 triggers a closer association of receptor clusters with DAP12. In contrast, the Pearson correlation coefficient between the KIR and DAP12 clusters was significantly lower in both resting and activated NKL/KIR2DS1^K233A^ cells (Figure 5B). Clusters of DAP12 were localized significantly further from KIR2DS1^K233A^ clusters than wild-type KIR2DS1 (Figures S7A and S7B) and less DAP12 was detected at the membrane (Figure S7C) consistent with the prediction that DAP12 does not associate with this receptor.

### Larger Clusters of KIR2DS1 Are More Often Associated with Clusters of DAP12

Ligation of KIR2DS1 also led to an increase in the mean area of the receptor clusters (Figure S7D). There was also a small but not significant increase in the area of DAP12 clusters (Figure S7E). Thus, we set out to investigate whether the size of KIR2DS1 clusters is important for its signaling. To test this, we exploited the inherent heterogeneity in the size of KIR nanoclusters within individual cells. Standard methods to quantify protein co-localization only take into account fluorescence that is directly overlapping, e.g., within one pixel, and do not report lateral connections between nanoclusters, such as those apparent in our images. Therefore, to quantify the association between clusters, we screened a circular area around the centroid of each KIR2DS1 cluster for the presence of DAP12 clusters. KIR2DS1 clusters that were associated with DAP12 were split into tertiles according to their area (designated small, medium, or large; Figures 5D–5K). This demonstrated that both in resting cells and upon KIR2DS1 ligation, the largest clusters of KIR2DS1 were most often found in association with DAP12 clusters, accounting for more than half of all cluster interactions (Figures 5F and 5I).

The amount of DAP12 proximal to KIR2DS1 clusters was also higher for large clusters (Figures 5H and 5K). Interestingly, a similar frequency of contacts between differently sized KIR clusters and DAP12 was calculated from imaging data altered so that the positions of DAP12 clusters were randomized within the cell area (Figures 5G and 5J). Thus, large clusters of KIR2DS1 are inherently more likely to associate with clusters of DAP12 on account of their size.

### The Size of KIR2DS1 Nanoclusters Is Important for Recruitment and Phosphorylation of ZAP-70

**Figure 6:**
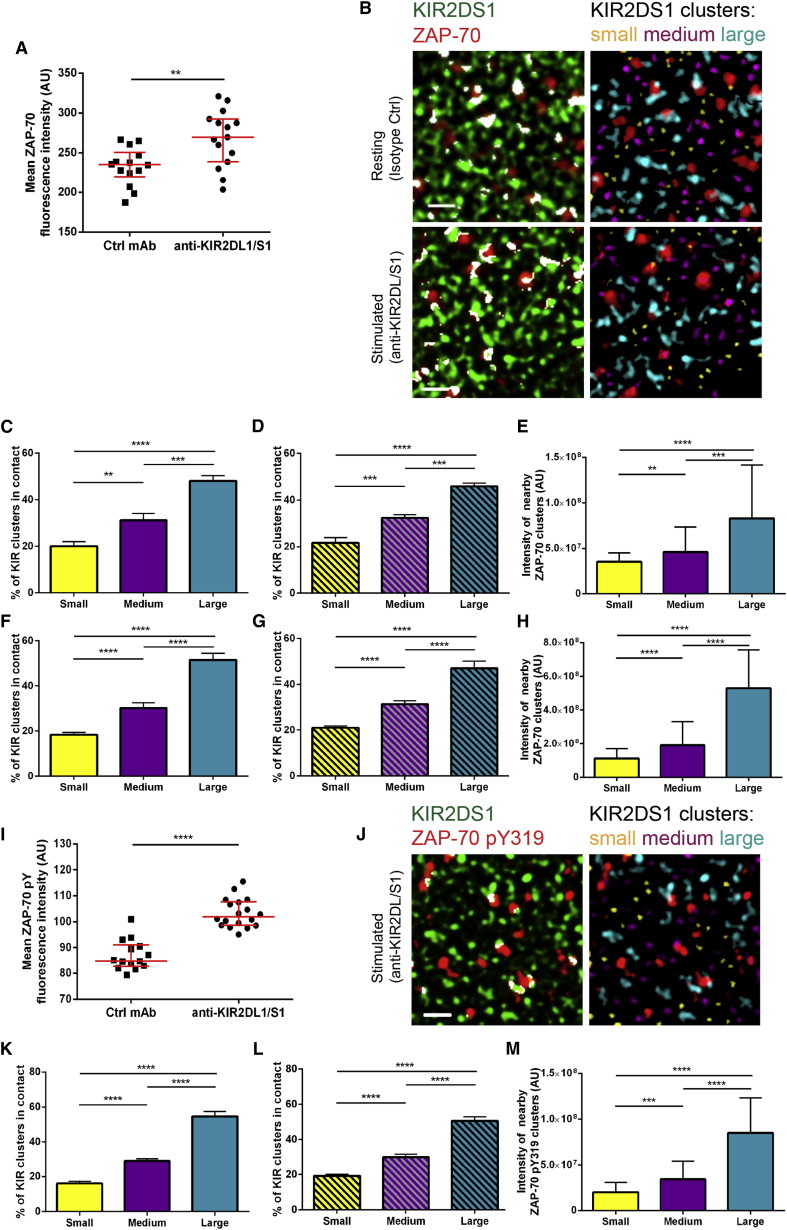
ZAP-70 Is More Often Recruited to and Phosphorylated at Larger KIR2DS1 Clusters (A–M) NKL/KIR2DS1-HA cells were incubated for 5 min on isotype control-coated or anti-KIR2DL/S1 mAb-coated slides, fixed, stained with anti-HA mAb (AF488) and anti-ZAP-70 Ab (AF568; A–H) or anti-ZAP-70 pY319 Ab (AF568; I–M) and imaged with STED microscopy. (A and I) Mean ZAP-70 (A) or ZAP-70 pY319 (I) fluorescence intensity in NKL/KIR2DS1-HA cells on isotype control-coated and anti-KIR2DL/S1 mAb-coated slides. (B and J) 3 × 3 μm regions of cell membrane with KIR2DS1 (green) and ZAP-70 (B) or ZAP-70 pY319 (J; red) with overlapping pixels marked in white (left) or with KIR2DS1 clusters color-coded according to size (right). Each size bin corresponds to one-third of all KIR2DS1 clusters. (C and F) The fractions of all KIR2DS1 clusters associated with ZAP-70 that fall into each size group in cells on isotype control (C) or anti-KIR2DL/S1 (F) coated slides. (D and G) The fractions of all KIR2DS1 clusters associated with ZAP-70 that fall into each size group in simulated data where the positions of KIR2DS1 clusters were maintained and positions of ZAP-70 clusters were randomized within the boundaries of cell area, in cells on isotype control (D) or anti-KIR2DL/S1 (G) coated slides. (E and H) Average intensity of ZAP-70 clusters found nearby KIR2DS1 clusters from specified size bins in cells on isotype control (E) or anti-KIR2DL/S1 (H) mAb-coated slides. (K) The fraction of all KIR2DS1 clusters associated with ZAP-70 pY319 that fall into each size group, in cells on anti-KIR2DL/S1 mAb. (L) The fraction of all KIR2DS1 clusters associated with ZAP-70 pY319 that fall into each size group where ZAP-70 pY319 clusters were randomized within the boundaries of cell area. (M) Average intensity of ZAP-70 pY319 clusters found nearby KIR2DS1 clusters from each size bin. (A and I) Each symbol represents one cell. Horizontal bars and errors represent the medians and interquartile range. (C–H and K–M) Bars and errors represent the medians and interquartile range. The data are from 13–18 cells from two independent experiments (A and I) or 29–39 cells from three independent experiments (C–H and K–M). AU, arbitrary units. ^∗∗^p < 0.01,^∗∗∗^p < 0.001, ^∗∗∗∗^p < 0.0001, Mann- Whitney test (A) and (I) or row-matched Friedman test with Dunn’s post-test (C–H and K–M). See also Figure S7.

To determine whether the size of KIR2DS1 nanoclusters also affects downstream signaling, we next imaged this receptor and the membrane-proximal kinase ZAP-70 which participates in KIR2DS1 signaling (Lanier et al., 1998), in NK cells plated on isotype-matched or anti-KIR2DL/S1 mAb-coated slides (Figure 6). Ligation of wild-type KIR2DS1, but not KIR2DS1^K233A^, led to an increase in the amount of ZAP-70 (Figures 6A and S7F) and its activated form phosphorylated on tyrosine 319 (ZAP-70 pY319; Figures 6I and S7G) proximal to the membrane at the contact site. Many clusters of KIR2DS1 (Figures 6B and 6J, green) were not associated with clusters of ZAP-70 or pZAP-70 (red). However, most clusters of the kinase did connect with the periphery of KIR2DS1 nanoclusters (overlap highlighted in white; Figures 6B and 6J). Clusters of ZAP-70 were preferably found in contact with larger clusters of KIR2DS1 in both resting and stimulated cells (Figures 6C and 6F). Only very few clusters of ZAP-70 pY319 were detected in unstimulated cells, but in stimulated cells, clusters of phosphorylated ZAP-70 were more often found near the largest KIR clusters (Figure 6K).

Comparison with simulated datasets, in which the positions of ZAP-70 clusters were randomized, indicated that large clusters were intrinsically more likely to contact clusters of ZAP-70 or ZAP-70 pY319 (Figures 6D, 6G, and 6L). The amount of ZAP-70 and phosphorylated ZAP-70 also increased when associated with larger KIR clusters (Figures 6E, 6H, and 6M). Thus, larger clusters are inherently more likely to contact a cluster of ZAP-70 and trigger its phosphorylation.

### The Size of KIR2DL1 Nanoclusters Is Important for SHP-1 Phosphorylation

**Figure 7:**
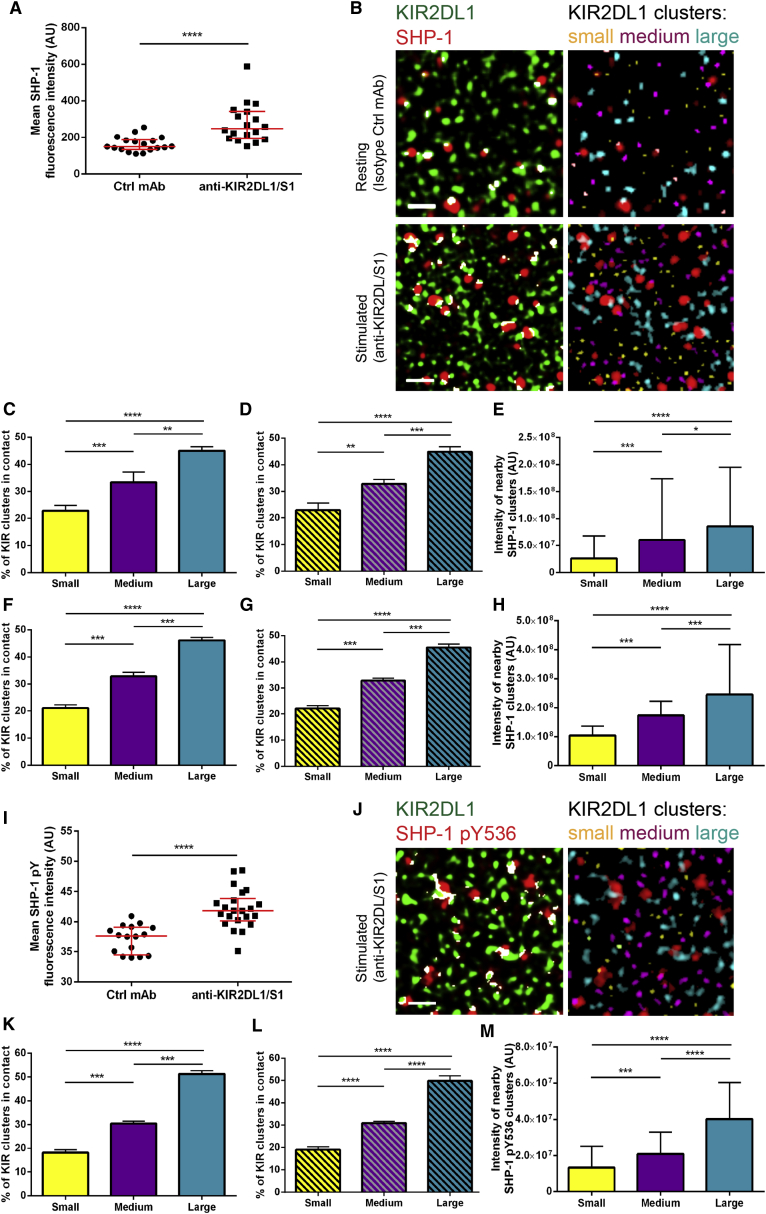
SHP-1 Is More Often Recruited to and Phosphorylated at Larger KIR2DL1 Clusters (A–M) NKL/KIR2DL1-HA cells were incubated for 5 min on isotype control-coated or anti-KIR2DL/S1 mAb-coated slides, fixed, stained with anti-HA mAb (AF488) and anti-SHP-1 mAb (AF568; A–H) or anti-SHP-1 pY536 Ab (AF568; I–M) and imaged with STED microscopy. (A and I) Mean SHP-1 (A) or SHP-1 pY536 (I) fluorescence intensity in NKL/KIR2DL1-HA cells on isotype control-coated and anti-KIR2DL/S1 mAb-coated slides. (B and J) 3 × 3 μm regions of cell membrane with KIR2DL1 (green) and SHP-1 (B; red) or SHP-1 pY536 (J; red) with overlapping pixels marked in white (left) or with KIR2DL1 clusters color-coded according to their size (right). Each size bin corresponds to one-third of all KIR2DL1 clusters. (C and F) The fractions of all KIR2DL1 clusters associated with SHP-1 that fall into each size group, in cells on isotype control (C) or anti-KIR2DL/S1 (F) mAb. (D and G) The fractions of all KIR2DL1 clusters associated with SHP-1 that fall into each size group in simulated datasets where the positions of KIR2DL1 clusters were maintained and positions of SHP-1 clusters were randomized within the boundaries of cell area, in cells on isotype control (D) or anti-KIR2DL/S1 (G) mAb. (E and H) Average intensity of SHP-1 clusters found nearby KIR2DL1 clusters from specified size bins in cells on isotype control (E) or anti-KIR2DL/S1 (H) mAb. (K) The fraction of all KIR2DL1 clusters associated with SHP-1 pY536 that fall into each size group in cells on anti-KIR2DL/S1 mAb. (L) The fraction of all KIR2DL1 clusters associated with SHP-1 pY536 that fall into each size group in simulated datasets where the positions of KIR2DL1 clusters were maintained and positions of SHP-1 pY536 clusters were randomized within the boundaries of cell area. (M) Average intensity of SHP-1 pY536 clusters found nearby KIR2DL1 clusters from each size bin. (A and I) Each symbol represents one cell. Horizontal bars and errors represent the medians and interquartile range. (C–H and K–M) Bars and errors represent the medians and interquartile range. The data are from 14–22 cells from two independent experiments (A and I) or 27–33 cells from three independent experiments (C–H and K–M). AU, arbitrary units. ^∗^p < 0.05, ^∗∗^p < 0.01,^∗∗∗^p < 0.001, ^∗∗∗∗^p < 0.0001, Mann- Whitney test (A and I) or row-matched Friedman test with Dunn’s post-test (C–H and K–M). See also Figure S7.

To determine whether the size of KIR2DL1 nanoclusters also affects signaling, we imaged KIR2DL1 with SHP-1, the phosphatase recruited in response to inhibitory KIR ligation (Fry et al., 1996, Burshtyn et al., 1996), or SHP-1 phosphorylated on tyrosine 536 (SHP-1 pY536), as a mark of phosphatase activity (Zhang et al., 2003). Ligation of KIR2DL1, but not KIR2DL1^Y281A/Y311A^, led to an increase in the fluorescence intensity of membrane-proximal SHP-1 and pSHP1 (Figure 7A, 7I, S7H, and S7I). Larger clusters of KIR2DL1 were more often associated with clusters of SHP-1, under both resting and activating conditions (Figures 7B, 7C, and 7F). pSHP-1 clusters were rarely detected on slides coated with control mAb, while clusters of phosphorylated SHP-1 detected upon KIR2DL1 ligation were preferentially in the vicinity of large KIR2DL1 clusters (Figures 7J and 7K). The median amount of SHP-1 and SHP-1 pY536 was also highest within the proximity of large KIR clusters (Figures 7E, 7H, and 7M). The frequency of contacts between differently sized KIR clusters and SHP-1 calculated for simulated datasets in which positions of SHP-1 clusters were randomized, was again comparable to the experimental data (Figures 7D, 7G, and 7L). Thus, larger clusters of KIR2DL1 are inherently more likely to contact SHP-1 clusters for signaling.

## Discussion

Over the past decade, it has emerged that immune cell receptors and ligands are organized into micrometer-scale domains at cell surfaces and immune synapses, and this organization is important for signaling in T cells, B cells, NK cells, and other immune cells (Davis and Dustin, 2004, Harwood and Batista, 2008, Orange, 2008). KIRs, like other signaling receptors, assemble into microclusters where phosphorylation occurs (Davis et al., 1999, Treanor et al., 2006, Abeyweera et al., 2011). Recently however, super-resolution microscopy has forced a revision to our understanding of how immune cell receptors organize within the cell membrane, by revealing sub-micron nanoscale receptor assemblies. However, little is understood about the mechanisms or functions of receptor nanoclusters.

Many factors including cholesterol-rich membrane domains (lipid rafts) and cortical actin have been implicated in controlling the clustering of surface proteins (Saka et al., 2014, Lagrue et al., 2013). Here, we demonstrate that the size of KIR nanoclusters is influenced by the transmembrane sequence. The positively charged residue within the transmembrane domain creates larger nanoclusters of KIR2DS1. The transmembrane lysine can affect the organization of KIR2DS1 directly or perhaps indirectly, for example by modulating an interaction in the endoplasmic reticulum, which modifies the protein’s glycosylation (Mulrooney et al., 2013).

A crucial unknown is whether the size of immune receptor nanoclusters is functionally important. Primary antigen-experienced T cells have larger oligomers of TCR at their surface, which likely relate to TCR nanoclusters (Kumar et al., 2011). In NK cells, loss of histidine at position 36 in KIR2DL1 increases KIR self-association and ITIM phosphorylation (Kumar et al., 2015). B cell receptor, on the other hand, is signaling-incompetent within tight nanoclusters, but upon activation, clusters open up to permit signal amplification (Kläsener et al., 2014). However, from these studies, the importance of the size of nanoclusters is not obvious. To test whether the size of clusters affects signaling here, we directly imaged membrane-proximal signaling events by super-resolution microscopy. We found that SHP-1 is more efficiently recruited to and phosphorylated within larger KIR nanoclusters. Thus, inhibitory signaling would be preferentially transduced by larger nanoclusters of KIR2DL1. Similarly for activating signals, larger nanoclusters of KIR2DS1 were more often interacting with clusters of the signaling adaptor DAP12, and downstream kinase ZAP-70 was preferentially recruited to, and activated within, larger nanoclusters of KIR2DS1. This establishes a relationship between the size of NK cell receptor nanoclusters and signaling outcomes.

Comparison of the experimental data with simulated data in which the positions of signaling clusters were randomized, demonstrated that larger receptor clusters were inherently more likely to contact independently distributed clusters of signaling molecules. This implies that the size of protein nanoclusters can directly affect signaling efficiencies by influencing the likelihood that clusters meet by chance. Previous work from our lab established that cellular activation can affect the nanoscale organization of NK cell receptors (Pageon et al., 2013). Together with the current data, this implies that changes in nanoclustering could tune the thresholds at which immune cells are switched on and off, independently of changes in expression levels.

In addition to changing the sizes of clusters, cellular activation also changed the relative position of different nanoclusters. Here, we show that cellular activation led to increased coalescence of clusters of KIR2DS1 and its signaling adaptor DAP12. This may occur by recruitment of DAP12 clusters to the interface, since the level of expression of this adaptor increases upon ligation of KIR2DS1, as well as by a lateral reorganization of protein nanoclusters. Because KIR2DS1 only signals via this adaptor, the mixing of the two proteins would allow more KIR2DS1 molecules to become signaling-competent, thus providing a positive feedback-loop to amplify or sustain signaling. This model for NK cell activating receptors is reminiscent of a convergence of BCR and co-receptor CD19 postulated to be important for antigen stimulation of B cells (Mattila et al., 2013). Thus, coalescence of nanoclusters might be a common mechanism for spatiotemporal positive feedback for activating receptor signals. In summary, we have established that the nanometre-scale organization of NK cell receptors nanoclusters is controlled by the transmembrane sequence and affects signaling.

## Experimental Procedures

All donors were healthy and gave informed consent for their blood to be used in accordance with the Declaration of Helsinki (with ethics approved by The National Research Ethics Service, Ref 05/Q0401/108).

### Cell Lines

The NK cell line NKL (a kind gift from Jack Strominger, Harvard University), leukemic T cell line Jurkat E6.1 (TIB-152, American Type Culture Collection), as well as B cell lines 721.221/Cw4 and 721.221/Cw4/MICA (used as target cells, as previously described; Pageon et al., 2013) were cultured in RPMI 1640 (Sigma) supplemented with 10% fetal bovine serum (FBS; Invitrogen), 2 mM L-glutamine, and 1% penicillin and streptomycin (both GIBCO). NKL were cultured in the presence of IL-2 (100 U/ml; Roche). All cell lines were routinely tested for mycoplasma infection using a PCR-based kit (PromoCell). All transfectants were generated by retroviral transduction (as detailed in the Supplemental Experimental Procedures). Expression of the receptors was checked by flow cytometry, and their functionality was confirmed by assessing spreading response and IFN-γ production, as detailed in the Supplemental Experimental Procedures.

### Generation of NK Clones

NK clones were generated from NK cells freshly isolated from peripheral blood of healthy donors by negative magnetic selection (NK cell isolation kit, Miltenyi Biotec) by plating single NK cells in individual wells of 96-well plates (details can be found in the Supplemental Experimental Procedures). Cells were tested by flow cytometry for clonality and the presence of KIR2DS1 and KIR2DL1. Additionally, cells were checked for expression of KIR2DL3 due to cross-reactivity of the anti-KIR2DL/S1 mAb (EB6) with the KIR2DL3^∗^005 allele (Falco et al., 2010), using DX27 mAb (Biolegend, three clones), or 180701 mAb (R&D Systems, two clones), and 2DL3-positive cells were excluded.

### Sample Preparation for Imaging

Cells were plated on chambered glass coverslips pre-coated with PLL, fixed and blocked as detailed in the Supplemental Experimental Procedures. For stimulation of cells, slides were additionally coated with 5 μg/ml anti-KIR2DL/S1 mAb (clone EB6) or murine IgG1. KIR2DL1, KIR2DS1, IL-2R alpha subunit, DAP12, ZAP-70, ZAP-70 pY319, SHP-1, and SHP-1 pY536 were fluorescently labeled with antibody, as detailed in the Supplemental Experimental Procedures. To check for changes in membrane organization induced by contact with a glass surface we also compared KIR2DL1 and KIR2DS1 clustering in NK cells fixed in suspension, as detailed in the Supplemental Experimental Procedures.

### Microscopy

Single-molecule localization microscopy images were acquired on a Leica SR GSD microscope and STED images were acquired on Leica TCS SP8 STED CW microscope, as detailed in the Supplemental Experimental Procedures.

### Image Analysis

GSD image reconstruction was performed using Thunderstorm software (Ovesný et al., 2014), as detailed in the Supplemental Experimental Procedures. Multiple regions of 3 × 3 μm were selected for quantitative analysis based on Ripley’s *K* function (Ripley, 1977) and univariate Getis and Franklin’s local point pattern analysis (Getis and Franklin, 1987) method, as described previously (Williamson et al., 2011, Pageon et al., 2013).

For comparison of the intensity of fluorescence derived from signaling molecules in STED images, the mean fluorescence intensity per pixel was measured in raw STED images in ImageJ. The size of pixel in the compared images was kept constant. To compare the area of clusters, the co-localization of proteins and the distances between cluster centroids as well as to identify clusters localized within a close proximity, STED images were deconvolved in Huygens Professional 10.1 software (Scientific Volume Imaging). Images were converted into binary maps for quantitative comparison of clusters and measurements of the distances between cluster centroids. To assess protein co-localization, Pearson correlation coefficients were calculated using an ImageJ (Schneider et al., 2012) plug-in Coloc 2 (written by Daniel J. White, Tom Kazimiers, and Johannes Schindelin; available online), which uses the Costes auto-threshold.

To identify clusters of signaling molecules overlapping or in contact with KIR clusters or in proximity, a circular area around the centroid of each KIR2DL/S1 cluster was searched for the presence of DAP12/SHP-1/ZAP-70 clusters. The results were compared to simulated datasets in which positions of signaling molecules clusters were randomized within the cell area. Fractions of KIR2DL/S1 clusters with at least one signaling molecule cluster nearby and relative efficiency of phosphorylation within clusters of certain sizes were quantified in a custom- MATLAB script.

Contrast and brightness of the representative images in the figures has been adjusted to make details visible but all quantitative analysis has been performed on unprocessed files and datasets throughout the manuscript.

Detailed information on image analysis can be found in the Supplemental Experimental Procedures.

### Statistical Analysis

Sample sizes chosen were appropriate to provide enough power for statistical tests used in this study (StatMate software, GraphPad Software). The statistical significance of differences between two datasets was assessed by Mann-Whitney test comparing ranks; multiple comparisons were made with Kruskal-Wallis test by ranks or matched-values Friedman test with Dunn’s post-testing. All statistical analyses were performed using GraphPrism 6.0 (GraphPad Software).

## Author Contributions

A.O., S-P.C., and D.M.D. conceived the project; A.O., S-P.C., and K.S. generated cell lines and NK clones; A.O. and D.M.D designed experiments and wrote the manuscript; A.O performed most of the experiments; D.J.M. and P.R.K performed additional experiments; D.J.W wrote MATLAB scripts; and A.O. and D.J.W. analyzed the data.

## Supplemental Information

Document S1. Supplemental Experimental Procedures, Figures S1–S7, and Table S1

Document S2. Article plus Supplemental Information
