# Supplementary material for: The Size of Activating and Inhibitory Killer Ig-like Receptor Nanoclusters Is Controlled by the Transmembrane Sequence and Affects Signaling
Source: Cell Rep. 2016 May 19;15(9):1957–72. doi: 10.1016/j.celrep.2016.04.075 (PMC4893158; doi:10.1016/j.celrep.2016.04.075)
Supplement: Document S1. Supplemental Experimental Procedures, Figures S1–S7, and Table S1 [file mmc1.pdf]

Cell Reports, Volume *15*

Supplemental Information

The Size of Activating and Inhibitory Killer Ig-like Receptor Nanoclusters Is Controlled by the Transmembrane Sequence and Affects Signaling

Anna Oszmiana, David J. Williamson, Shaun-Paul Cordoba, David J. Morgan, Philippa R. Kennedy, Kevin Stacey, and Daniel M. Davis

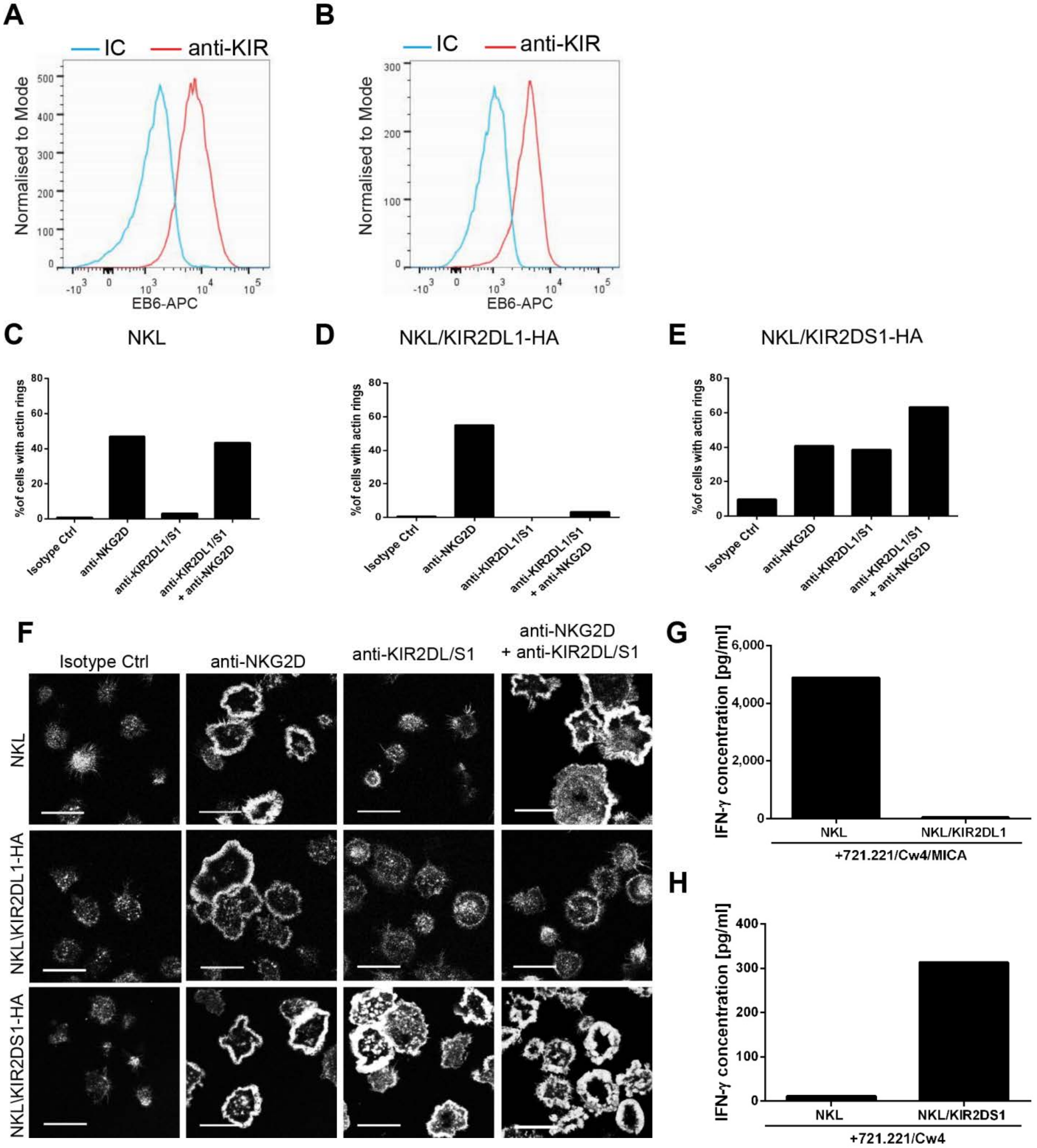

Figure S1. Generation and characterization of NKL/KIR2DL1-HA and NKL/KIR2DS1-HA transfectants (referring to figure 1). NKL/KIR2DL1-HA and NKL/KIR2DS1-HA transfectants were created by retroviral transduction of the human NK cell line NKL. NKL/KIR2DL1-HA (**A**) and NKL/KIR2DS1-HA (**B**) cells (red) were stained with anti-KIR2DL/S1 mAb (clone EB6) conjugated with APC and analyzed by flow cytometry. Respective cells stained with isotype-matched control mAb (blue) were analyzed as negative controls. (**C**-**F**) Parental NKL cells (**C**), NKL/KIR2DL1-HA (**D**) and NKL/KIR2DS1-HA (**E**) transfectants were incubated for 10 min at 37°C on slides coated with anti-KIR2DL/S1 or anti-NKG2D mAb at 5 µg/ml plus 5 µg/ml of murine IgG1, 10 µg/ml of IgG1 or 5 µg/ml of anti-KIR2DL/S1 plus 5 µg/ml of anti-NKG2D mAb. Filamentous actin was visualized using fluorescently labeled phalloidin and percentages of cells forming peripheral actin rings were quantified. One representative experiment of two is shown, $n \geq 100$ cells per condition. Representative fields of view are shown in (**F**). Scale bars 5 µm. (**G**-**H**) Parental NKL cells and NKL/KIR2DL1-HA (**G**) or NKL/KIR2DS1-HA (**H**) were co-cultured for 24 hours with 721.221 transfectants expressing HLA-Cw4 and MICA (**G**) or -Cw4 only (**H**). IFN-γ release was measured by ELISA. E:T ratio was 10:1 in all experiments. One representative experiment of two is shown.

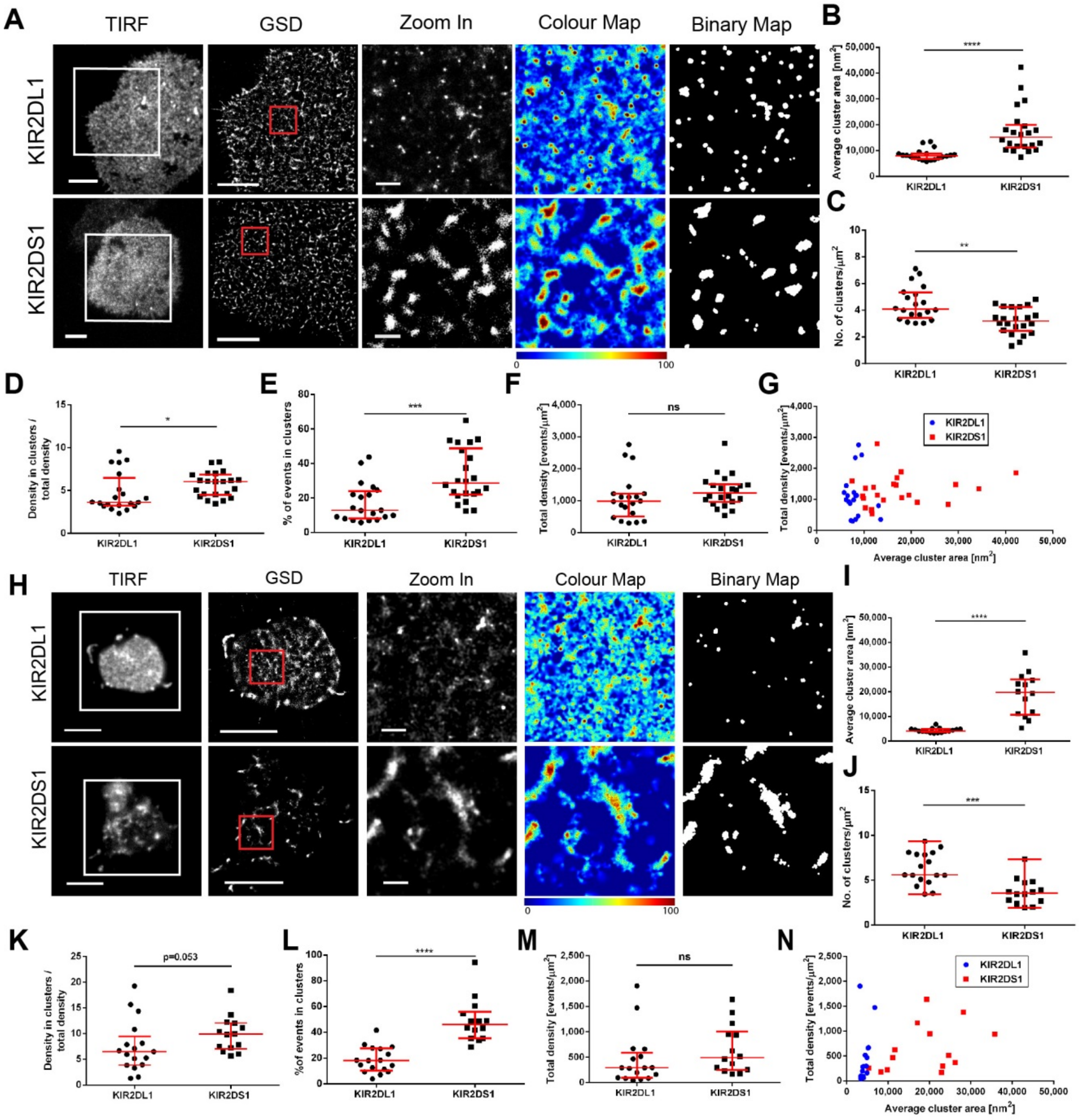

Figure S2. KIR2DL1-HA and KIR2DS1-HA display different clustering patterns at the surface of human NK cells irrespective of labeling strategy and contact with glass slides (referring to figure 1). (**A-G**) NKL/KIR2DL1-HA and NKL/KIR2DS1-HA cells were incubated on slides coated with PLL and stained with anti-HA mAb labeled with AF488. (**A**) Representative TIRF and GSD images of NKL/KIR2DL1-HA and NKL/KIR2DS1-HA cells stained with anti-HA mAb. The 3 × 3 μm regions (red boxes in GSD images) are zoomed-in and shown with corresponding color maps and binary maps. Colors correspond to the L(30) values. (**B** to **F**) Quantitative analysis of KIR2DL1 and KIR2DS1 clustering in NKL/KIR2DL1-HA and NKL/KIR2DS1-HA cells stained with anti-HA mAb. (**G**) Total density of detected molecules plotted against average cluster area measured for individual NKL/KIR2DL1-HA and NKL/KIR2DS1-HA cells stained with anti-HA mAb. Data are from 20 (KIR2DL1) and 22 (KIR2DS1) cells from three independent experiments. (**H** to **N**) The same cells were fixed in suspension, stained with anti-KIR2DL/S1 mAb labeled with AF647, thoroughly washed and added to chambered glass slides pre-coated with PLL. (**H**) Representative TIRF and GSD images of NKL/KIR2DL1-HA and NKL/KIR2DS1-HA cells fixed in suspension. The 3 × 3 μm regions are zoomed-in and shown with corresponding color maps and binary maps. (**I** to **M**) Quantitative analysis of KIR2DL1 and KIR2DS1 clustering in NKL/KIR2DL1-HA and NKL/KIR2DS1-HA cells fixed in suspension. (**N**) Total density of detected molecules plotted against average cluster area measured for individual NKL/KIR2DL1-HA and NKL/KIR2DS1-HA cells fixed in suspension. Data are from 17 (KIR2DL1) and 14 (KIR2DS1) cells from two independent experiments. Each symbol represents the mean from several regions within one cell. Horizontal bars and errors represent the medians and interquartile range. ns non-significant, * $p < 0.05$, ** $p < 0.01$, *** $p < 0.001$, **** $p < 0.0001$, Mann-Whitney test. Scale bars 5 μm in TIRF images and 500 nm in zoomed-in regions.

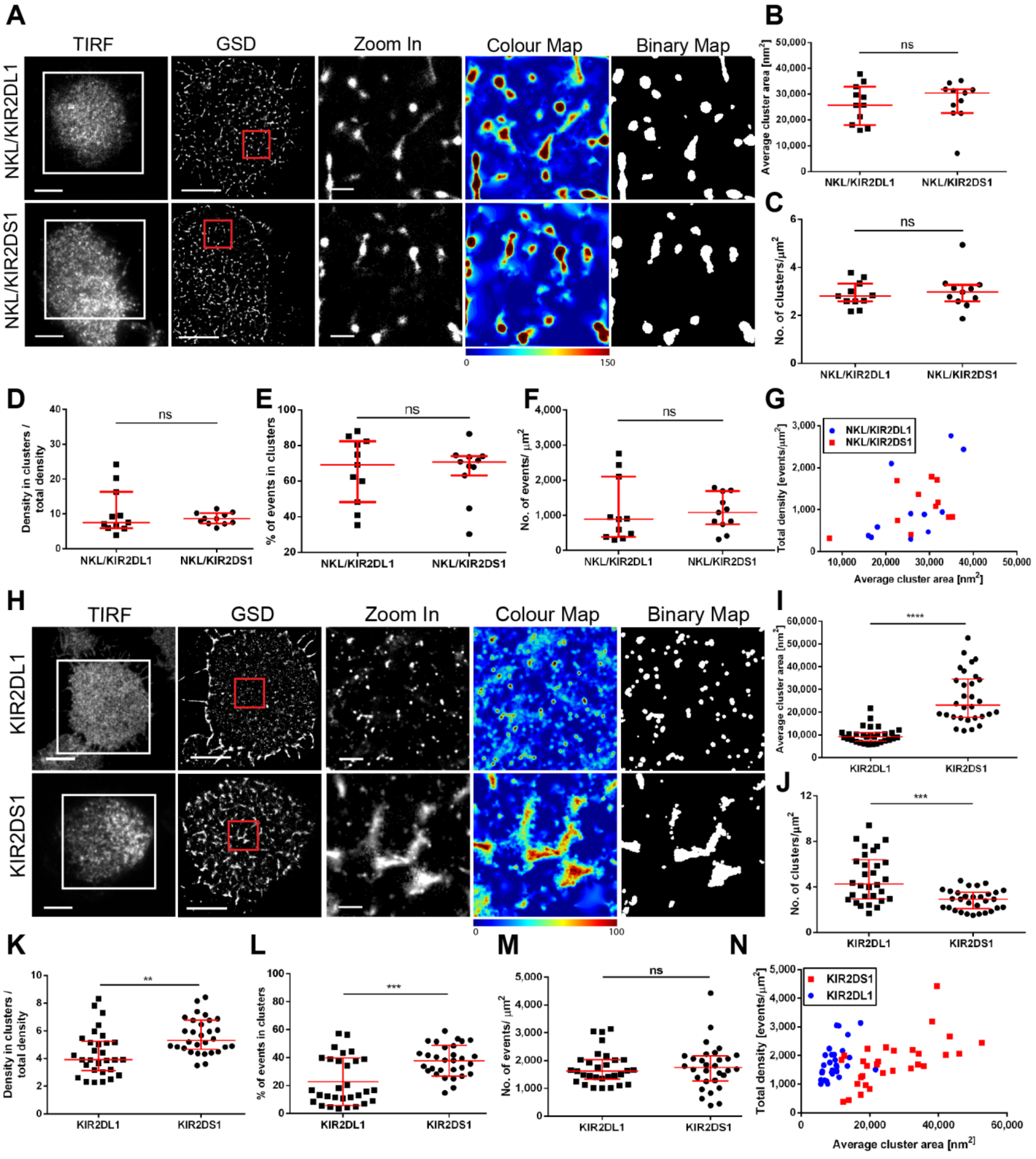

Figure S3. Differential organization of KIR2DL1 and KIR2DS1 is not a result of general differences in plasma membrane organization and does not depend on the association with DAP12 (referring to figure 1 and 5). (**A**) Representative TIRF and GSD images of IL-2Rα labeled with AF 488 in NKL/KIR2DL1-HA and NKL/KIR2DS1-HA cells on slides coated with PLL. The 3 × 3 µm regions (red boxes in GSD images) are zoomed-in and shown with corresponding color maps and binary maps. (**B** to **G**) Quantitative analysis of IL-2Rα clustering in NKL/KIR2DL1-HA and NKL/KIR2DS1-HA cells. (**G**) Total density of detected events plotted against average IL-2Rα cluster area in individual NKL/KIR2DL1-HA and NKL/KIR2DS1-HA cells. (**H**) Representative TIRF and GSD images of Jurkat/KIR2DL1-HA and Jurkat/KIR2DS1-HA cells stained with anti-HA mAb labeled with AF488. The 3 × 3 µm regions (red boxes) are zoomed-in and shown with corresponding color and binary maps. (**I** to **M**) Quantitative analysis of KIR2DL1 and KIR2DS1 clustering in Jurkat cells. (**N**) Total density of detected events plotted against average KIR2DL1 and KIR2DS1 cluster area in individual Jurkat cells. (**B - G** and **I - N**) Each symbol represents the mean from several regions within one cell. Horizontal bars and errors represent the medians and interquartile range. Data are from 11 cells from two independent experiments (**B** – **G**) or 30 cells from 3 independent experiments (**I** – **N**). Scale bars 5 µm in TIRF and 500 nm in zoomed-in regions. ns non-significant, ** $p < 0.01$, *** $p < 0.001$, Mann-Whitney test.

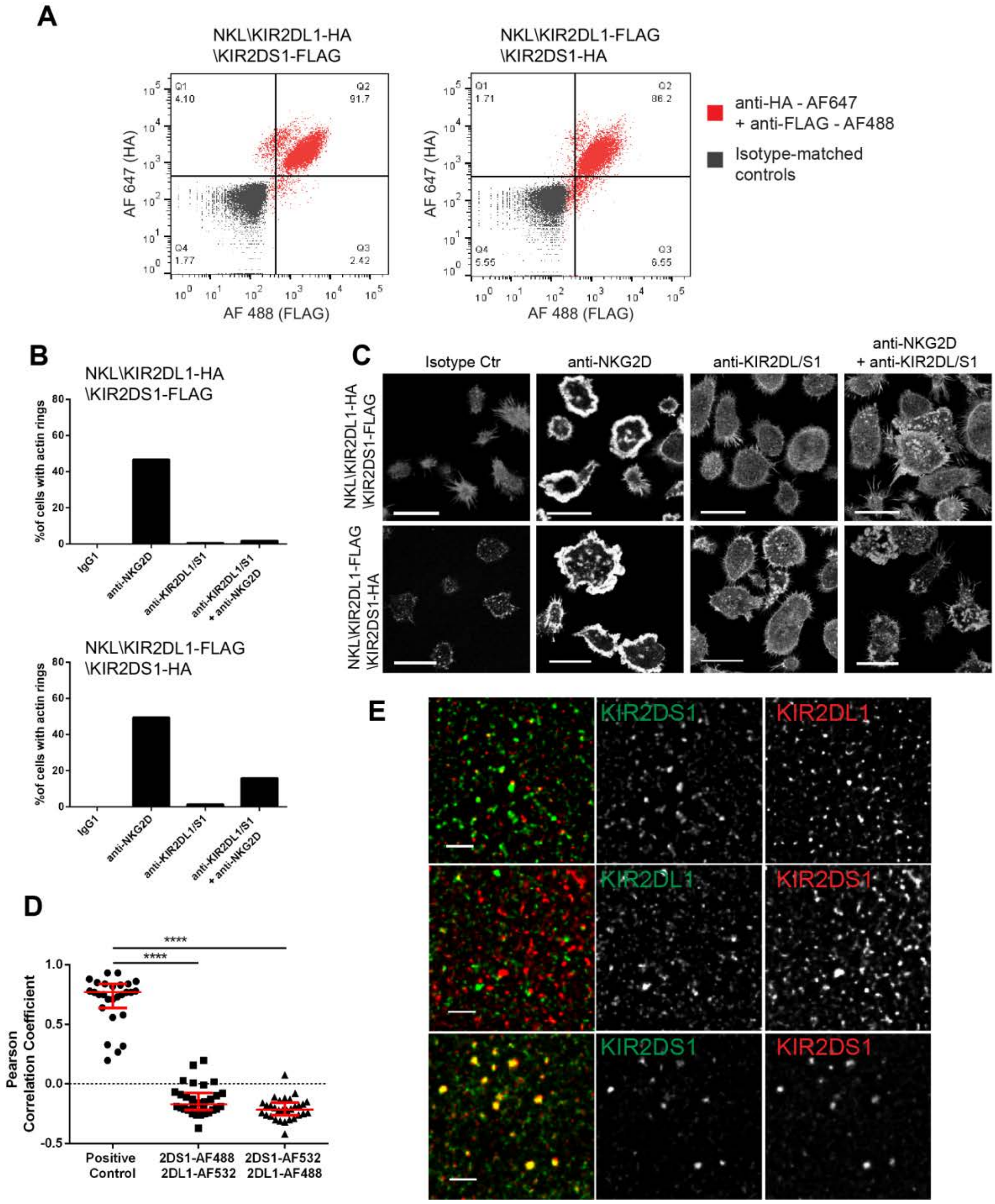

Figure S4. KIR2DL1 and KIR2DS1 are not localized in the same clusters in double-positive cells (referring to figure 1). NKL/KIR2DL1-HA/KIR2DS1-FLAG and NKL/KIR2DL1-FLAG/KIR2DS1-HA cell lines were created by retroviral transduction of human NK cell line NKL. (**A**) NKL/KIR2DL1-HA/KIR2DS1-FLAG and NKL/KIR2DL1-FLAG/KIR2DS1-HA cells were fixed, permeabilized, stained with anti-HA mAb conjugated with AF647 and anti-FLAG mAb conjugated with AF488 (red) and analyzed by flow cytometry. Respective cells stained with isotype-matched control mAb (grey) were analyzed as negative controls. (**B**-**C**) NKL/KIR2DL1-HA/KIR2DS1-FLAG and NKL/KIR2DL1-FLAG/KIR2DS1-HA transfectants were incubated for 10 min at 37°C on slides coated with anti-KIR2DL/S1 or anti-NKG2D mAb at 5 µg/ml plus 5 µg/ml of murine IgG1, 10 µg/ml of IgG1 or 5 µg/ml of anti-KIR2DL/S1 plus 5 µg/ml of anti-NKG2D mAb. Actin was visualized using fluorescently labeled phalloidin and percentages of cells forming peripheral actin rings were calculated. One representative experiment of three is shown, n ≥ 100 cells per condition. Representative fields of view are shown in (**C**). (**D**-**E**) NKL/KIR2DL1-FLAG/KIR2DS1-HA and NKL/KIR2DL1-HA/KIR2DS1-FLAG cells on PLL-coated slides were stained with anti-HA mAb (AF488) and anti-FLAG mAb (AF532) and imaged by STED microscopy. As a positive control, NKL/KIR2DS1-HA cells were stained with anti-HA mAb (AF488) and anti-KIR2DL/S1 mAb (AF532). (**D**) Pearson's correlation coefficient values calculated for KIR2DL1 and KIR2DS1 are compared to KIR2DS1 stained in both channels. Each symbol represents one cell. Horizontal bars and errors represent the medians and interquartile range. Data are from 27-30 cells from two (positive control) or three (KIR2DS1 vs. KIR2DL1) independent experiments. Representative 3 × 3 µm regions from STED images are shown in (**E**), scale bars 500 nm. *** $p < 0.001$, Kruskal-Wallis test with Dunn's post-test.

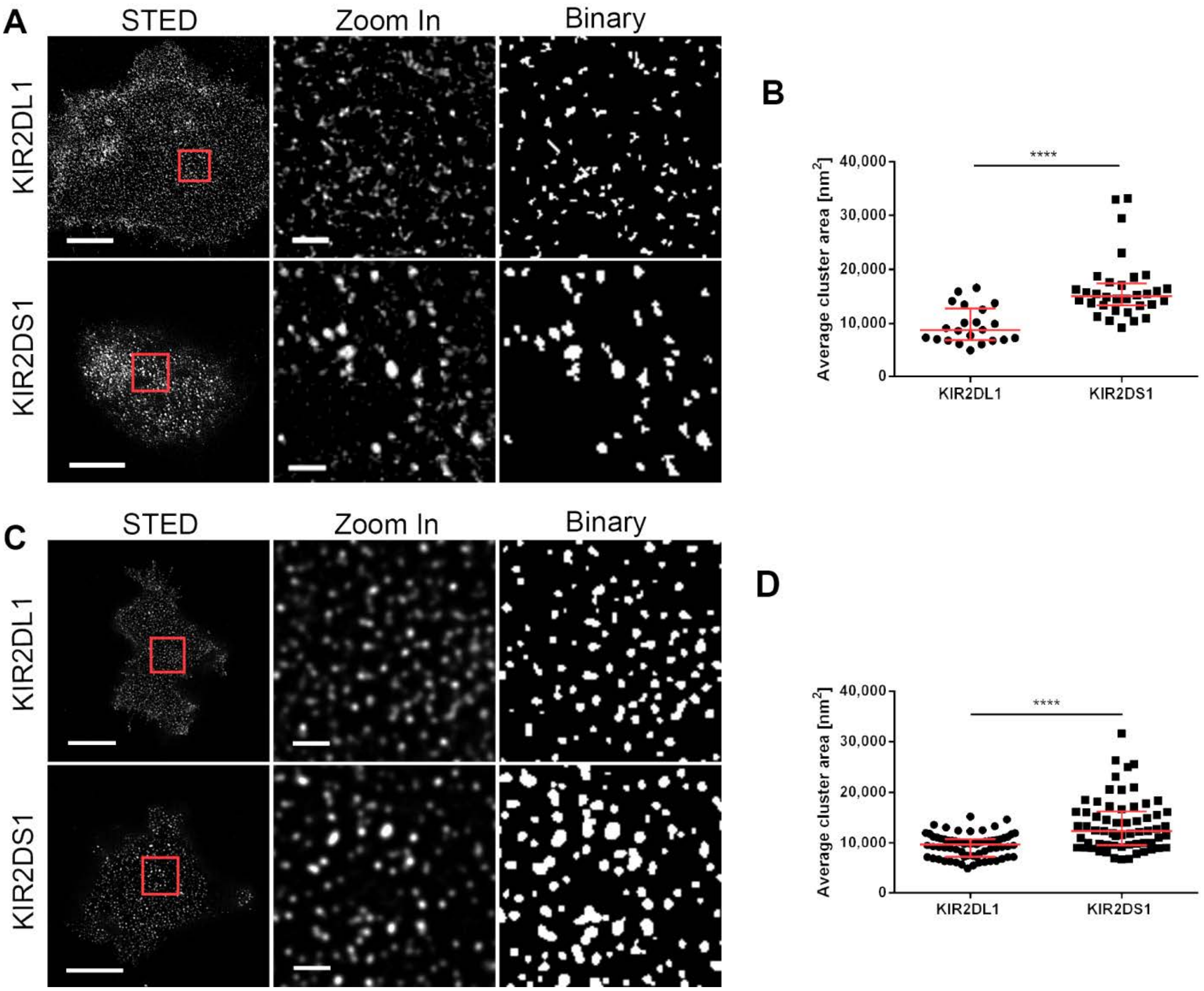

Figure S5. KIR2DL1 and KIR2DS1 are differentially organized at the surface of human NK cells as visualized by STED microscopy (referring to figure 1 and figure 2). NKL/KIR2DL1-HA and NKL/KIR2DS1-HA cells (**A**-**B**) as well as $2DS1^-/2DL1^+$ and $2DS1^+/2DL1^-$ NK clones (**C**-**D**) on PLL-coated slides were stained with anti-HA mAb conjugated to AF 488 (**A**-**B**) or anti-KIR2DL/S1 mAb conjugated to Atto 488 (**C**-**D**) and imaged by STED microscopy. (**A** and **C**) Representative STED images of KIR2DL1 and KIR2DS1 stained in NKL transfectants (**A**) and in primary NK clones (**C**). Scale bars 5 µm. The 3 × 3 µm regions outlined in red in STED images are zoomed-in and shown with corresponding binary maps (scale bars 500 nm). (**B**) Comparison of average cluster area between KIR2DL1 and KIR2DS1 in NKL/KIR2DL1-HA and NKL/KIR2DS1-HA cells, respectively. Data are from 22 (KIR2DS1) and 32 (KIR2DL1) cells from three independent experiments. (**D**) Comparison of average cluster area between KIR2DL1 and KIR2DS1 in $2DS1^-/2DL1^+$ and $2DS1^+/2DL1^-$ NK clones, respectively. Data are from 69 (KIR2DL1) and 62 (KIR2DS1) cells each from two clones derived from one donor. Each symbol represents the mean value from one cell. Horizontal bars and errors represent the medians and interquartile range. **** $p < 0.0001$, Mann-Whitney test.

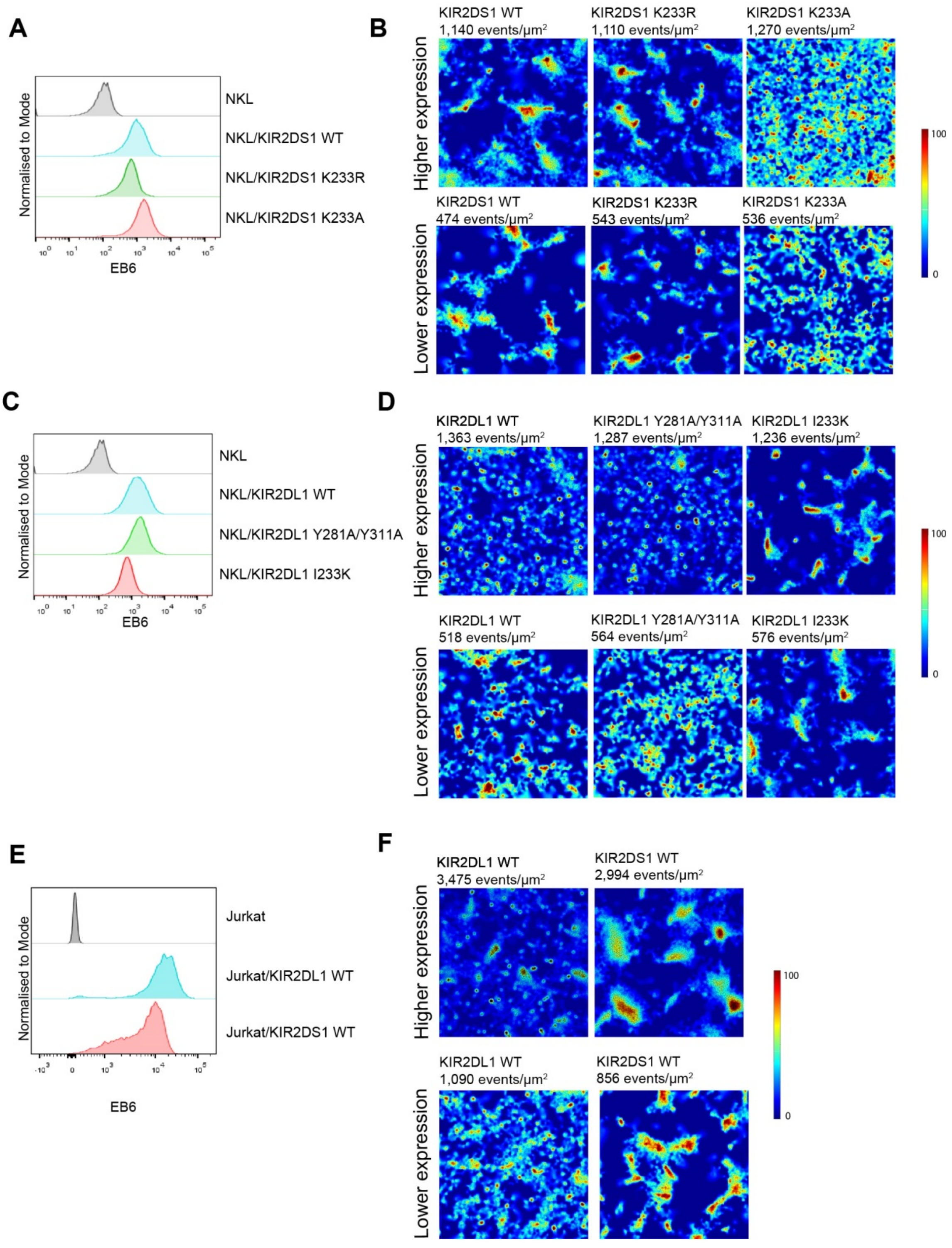

Figure S6. Differences in clustering of wild-type KIR2DS1 and KIR2DL1 and mutated receptors KIR2DS1^{K233A}, KIR2DS1^{K233R}, KIR2DL1^{I233K} and KIR2DL1$^{Y281A/Y311A}$ are not affected by relative density of detected molecules (referring to figure 3 and figure 4). (**A**) Parental NKL, NKL/KIR2DS1-HA, NKL/KIR2DS1^{K233A}, NKL/KIR2DS1^{K233R}, (**C**) Parental NKL, NKL/KIR2DL1-HA, NKL/KIR2DL1^{I233K} NKL/KIR2DL1$^{Y281A/Y311A}$, (**E**) Parental Jurkat, Jurkat/KIR2DL1-HA and Jurkat/KIR2DS1-HA cells were stained with anti-KIR2DL/S1 mAb (clone EB6) and analyzed by flow cytometry. (**B**, **D** and **F**) Wild-type and mutated forms of KIR2DS1 (**B**) and KIR2DL1 (**D**) in NKL and wild-type KIR2DL1 and KIR2DS1 in Jurkat (**F**) were stained with anti-HA mAb conjugated with AF488 and imaged by GSD microscopy. Selected 3 × 3 µm regions are shown as color maps where colors correspond to the extent of clustering according to pseudo-color scale. Regions from cells with relatively high (upper rows) and low (lower rows) densities of detected events are shown.

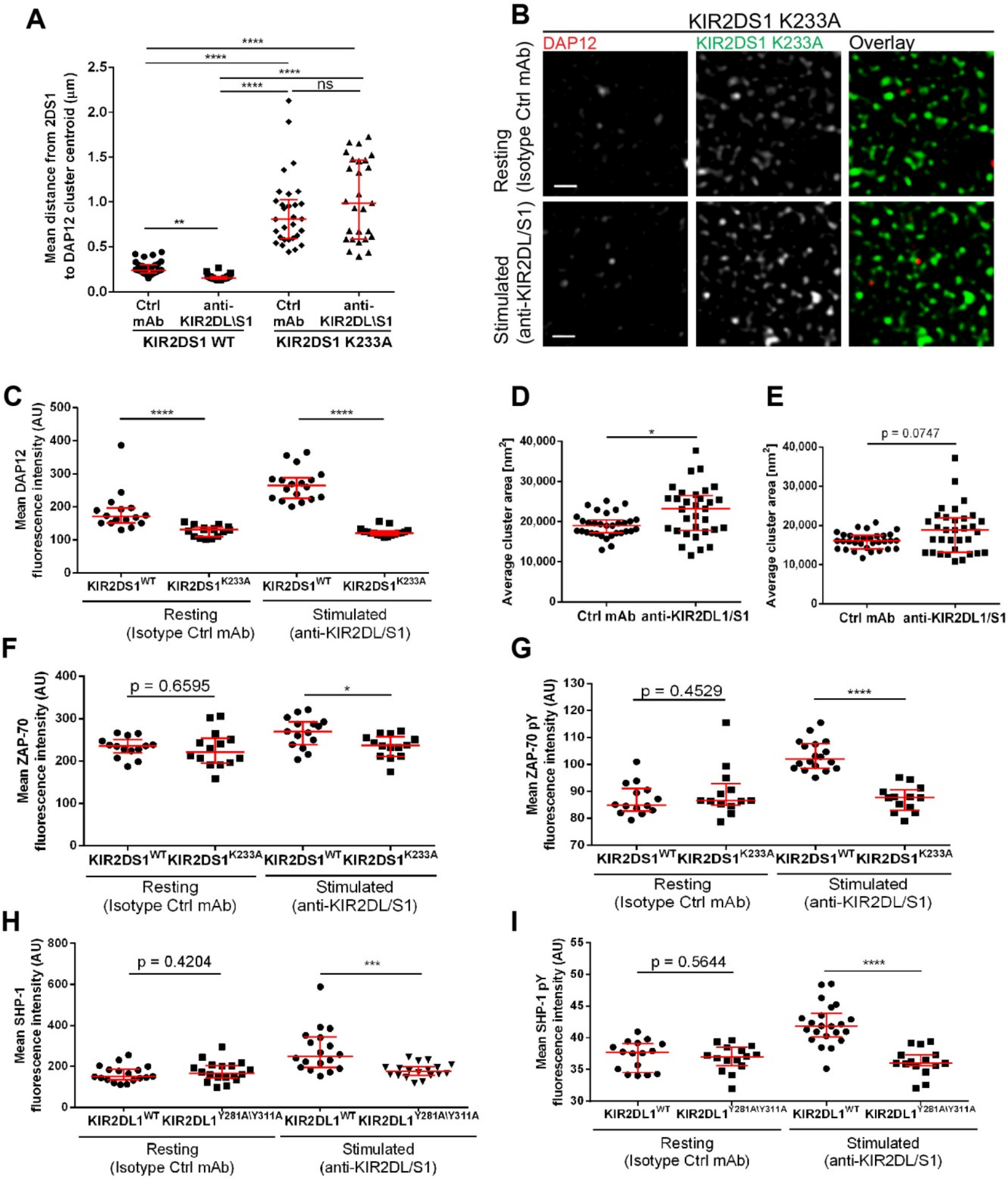

Figure S7. The effect of ligation of wild-type KIR2DL1 and KIR2DS1 and mutated receptors (referring to figure 5, figure 6 and figure 7). (A – E) NKL/KIR2DS1-HA or NKL/KIR2DS1^{K233A} cells were incubated on isotype control- or anti-KIR2DL/S1 mAb-coated slides, stained for KIR2DS1 (AF488) and DAP12 (AF568) and imaged by STED. (**A**) The average distances between centroids of KIR2DS1 clusters and a nearest cluster of DAP12. (**B**) Representative 3 × 3 µm regions from STED images of NKL/KIR2DS1^{K233A} cells with channels overlaid and separated. Scale bars 500 nm. (**C**) Mean DAP12-derived fluorescence intensity compared between NKL/KIR2DS1-HA and NKL/KIR2DS1^{K233A} cells on isotype control- and anti-KIR2DL/S1 mAb-coated slides. **(D – E)** Average area of KIR2DS1 (**D**) and DAP12 (**E**) clusters in NKL/KIR2DS1-HA cells on isotype control- or anti-KIR2DL/S1 mAb. (**F** and **G**) Mean ZAP-70- (**F**) and ZAP-70 pY319- (**G**) derived fluorescence intensity compared between NKL/KIR2DS1-HA and NKL/KIR2DS1^{K233A} cells on isotype control- and anti-KIR2DL/S1 mAb-coated slides. (**H** and **I**) Mean SHP-1- (**H**) and SHP-1 pY536- (**I**) derived fluorescence intensity compared between NKL/KIR2DL1-HA and NKL/KIR2DL1$^{Y281A/Y311A}$ cells on isotype control- and anti-KIR2DL/S1 mAb-coated slides. Each symbol represents one cell. Horizontal bars and errors represent the medians and interquartile range.

ns non-significant, * $p < 0.05$, ** $p < 0.01$, *** $p < 0.001$, **** $p < 0.0001$, Kruskal-Wallis test by ranks with Dunn's post-test (**A**) or Mann-Whitney test (**C** - **I**).

Supplemental Experimental Procedures

Plasmid generation.

The coding sequences of *KIR2DL1*002* and *KIR2DS1*00502* were ligated into a retroviral transfer vector pIB2 at the BamHI and EcoRI sites. HA and FLAG tags were then inserted at EcoRI site by non-directional ligation resulting in a glutamic acid, phenylalanine linker. Point mutations in KIR2DS1 and KIR2DL1 sequence were introduced by site-directed mutagenesis (Q5 Site-directed Mutagenesis Kit, New England Biolabs) using primers listed in Table S1.

Table S1. Primers used for site-directed mutagenesis (referring to figure 3 and figure 4).

**KIR2DS1 K233A**	Forward 5'CTCAGTGGTCGCCATCCCTTTCACC3'
	Reverse 5'GTCCCAATCAGAACATGTAG3'
**KIR2DS1 K233R**	Forward 5'CTCAGTGGTCCGGATCCCTTTCAC3'
	Reverse 5'GTCCCAATCAGAACATGTAG3'
**KIR2DL1 I233K**	Forward 5'CTCAGTGGTCAAGATCCTCTTCATC3'
	Reverse 5'GTCCCAATCAGAATGTGC3'
**KIR2DL1/Y281A/Y311A**	Forward 5'CCCTTCTCAGAGGCCCAAGACACCCCCAACAGATATCATCGTGGCCACGGAACTTCCAAATGCTG3'
	Reverse 5'CGAGTGATTTTTCTCTGTGTGAAAACGCAGTGATTCAACTGTGTGGCTGTCACCTCCTGAGGGTC3'

Retroviral transduction of NKL and Jurkat E6.1 cells.

The packaging cell line Phoenix-Amphotropic (Nolan Lab, Stanford) was transfected with Lipofectamine 3000 (Invitrogen) with a retroviral transfer vector pIB2 encoding KIR2DL1 or KIR2DS1 fused to HA tag or FLAG tag, or the same receptors in which single amino-acids were mutated. Viral supernatant collected 48 hours after transfection was used for three sequential centrifugations (300g, 45 min, 32°C) for infection of 10^6 cells. Cells expressing desired receptors were selected by addition of 10 µg/ml of blasticidin (InvivoGen) to the culture media.

NK clones generation.

Single NK cells were plated in individual wells of 96-well plates and cultured in clone medium (DMEM, 10% human serum, 15% F-12 HAMS, 1% penicillin and streptomycin, 2mM L-glutamine, 1mM sodium pyruvate, 1% MEM non-essential amino acids) in the presence of 10^8/ml irradiated (40 Gy) peripheral blood mononuclear cells (PBMCs) from two allogeneic individuals, 5×10^6/ml irradiated (40 Gy) RPMI 8866 cells, 400 U/ml of IL-2 and 5 ng/ml phytohemagglutinin for a week. On day 7, fresh clone media supplemented with 10^8/ml irradiated (40 Gy) PBMCs from two allogeneic individuals, 5×10^6/ml irradiated (40 Gy) RPMI 8866 cells and 400 U/ml of IL-2 were added. After a further seven days, cells were cultured in clone medium supplemented with 400 U/ml of IL-2 and passaged every 3 - 4 days.

Flow cytometry.

To assess surface expression of KIR2DL1-HA, KIR2DS1-HA and mutated forms of the receptors cells were washed in washing buffer (1% FBS/phosphate-buffered saline (PBS)), blocked with 1% AB human serum (Sigma)/washing buffer for 20 min at room temperature (RT) and stained with anti-KIR2DL/S1 mAb (clone EB6, Beckman Coulter) or isotype-matched control mAb (clone P3.6.2.8.1, eBioscience) conjugated with APC for 30 min at 4°C.

To assess expression of KIR2DL1-HA and KIR2DS1-FLAG or KIR2DL1-FLAG and KIR2DS1-HA in double transfectants, cells were fixed by resuspending in 4% PFA/PBS, incubated for 30 min incubation at RT, washed and blocked with 1% AB human serum/washing and permeabilizing buffer (1% FBS/0.1% saponin (Sigma)/PBS). Cells were then stained with 5 µg/ml of anti-HA mAb (clone 6E2, Cell Signaling) conjugated with AF647 and 5 µg/ml of anti-DYKDDDK Tag mAb (rabbit polyclonal, Cell Signaling) conjugated with AF488 or appropriate isotype-matched control Ab (mouse IgG1 isotype control, clone MOPC-21 from Biolegend and rabbit IgG isotype control, #4340 from Cell Signaling Technology) for 30 min at 4°C. Cells were then washed with washing buffer, fixed in 2% paraformaldehyde/PBS, assessed by BD FACS Canto II flow cytometer (BD Biosciences) and analyzed (FlowJo_V10 software).

Activation on slides assay.

Chambered glass coverslips (#1.5 Lab-Tek, Nunc) were coated with 0.01% poly-L-lysine (PLL, Sigma) and anti-KIR2DL/S1 (clone EB6) or anti-NKG2D (clone 149810; R&D Systems) mAb at 5 µg/ml plus 5 µg/ml of control murine IgG1 (clone MOPC-21, Biolegend), 10 µg/ml of IgG1 or 5 µg/ml of anti-KIR2DL1 plus 5µg/ml of anti-NKG2D mAb. Cell were allowed to settle on the slides for 10 min at 37°C and fixed in 4% PFA/PBS at RT for 30 min. Actin was visualized with AF488-labeled phalloidin (Invitrogen, 1/200 dilution) in 0.1% saponin/PBS and imaged by confocal microscopy (Leica TCS SP8 STED CW microscope). Images were exported to ImageJ and the percentage of cells forming peripheral actin rings was quantified.

Enzyme-linked immunosorbent assay (ELISA).

To assess the effect of KIR2DL1 ligation on the NKG2D-mediatd production of IFN-γ, parental NKL and NKL/KIR2DL1-HA cells were co-incubated with 721.221 target cells transfected to express MICA (a ligand for NKG2D) and HLA-Cw4 (a ligand for KIR2DL1 and KIR2DS1; 721.221/MICA/Cw4). To assess the effect of KIR2DS1 ligation on the production of IFN-γ, parental NKL and NKL/KIR2DS1-HA cells were co-incubated with 721.221 target cells transfected to express HLA-Cw4 only (721.221/Cw4). Cells were co-incubated with at an E:T ratio of 10:1 (200,000 NK cells and 20,000 target cells) for 24 hours. ELISA plates were coated with anti-IFN-γ mAb (clone NIB42, BD Biosciences, 1 µg/ml) in binding buffer (carbonate bicarbonate; Sigma) and blocked with 1% bovine serum albumin (BSA)/0.05% Tween-20/PBS. Supernatants were added in triplicate to the plate and incubated for 1 hour at RT. Plates were washed and incubated with biotinylated IFN-γ mAb (clone 4S.B3, BD Biosciences, 1 µg/ml) and then streptavidin HRP (BD Biosciences). The plates were developed with TMB ELISA substrate (Sigma-Aldrich); reaction was stopped with 1 N H_2SO_4. Absorbance was measured at 450 nm using a 570 nm reference line.

Sample preparation for imaging.

Chambered glass coverslips were coated with PLL and used for imaging of unstimulated cells or coated with 5 µg/ml anti-KIR2DL/S1 mAb (clone EB6) or murine IgG1 in PBS overnight at 4°C for stimulation of cells. Cells were allowed to settle on the slides for 10 min (resting on PLL) or 5 min (stimulating and isotype control-coated surfaces) at 37°C, fixed with 4% PFA/PBS for 30 min at RT and washed three times in PBS. Samples were blocked in 4% BSA/PBS for 40 min at RT. For immunostaining antibodies were diluted in 0.2% BSA/PBS at 5 µg/ml and where indicated cells were permeabilized with 0.1% saponin/0.2%BSA/PBS.

To visualize KIR2DL1 and/or KIR2DS1 cells were stained with following Ab: anti-KIR2DL/S1 (clone EB6) labeled in-house with Atto488 (Atto Tec), AF532 or AF647, anti-HA labeled with AF488 (clone 16B12, Invitrogen), anti-FLAG (clone M2; Sigma) conjugated in house with AF532 or rabbit anti-HA (C29F4, Cell Signaling Technology) followed by fluorescently labeled anti-rabbit secondary Ab (Invitrogen). The number of fluorophore molecules per antibody was 6 – 8. To visualize other proteins, the following primary Ab were used: anti-IL-2R alpha subunit (clone 7G7/B6, Millipore), anti-DAP12 (rabbit mAb, clone EPR5173, Abcam), anti-ZAP-70 (rabbit polyclonal, ab134509, Abcam) anti-ZAP-70 pY319 (rabbit polyclonal, ab131270 Abcam), anti-SHP-1 (rat mAb, clone 255402, R&D Systems), anti-SHP-1 pY536 (rabbit polyclonal, AP08023PU-N, Acris GmbH,); followed by fluorescently labeled secondary antibodies (Invitrogen). Staining was performed at RT for 60 min. Samples were then washed and imaged. GSD imaging was performed in 50 mM Tris-HCI (pH 8.0) with addition of 10 mM NaCl, 10% (w/v) Glucose, 1% (v/v) 2-mercaptoethanol, 0.7 mg/ml glucose oxidase and 42.5 µg/ml catalase.

Preparation of cells fixed in suspension.

To check for changes in membrane organization induced by contact with a glass surface we compared KIR2DL1 and KIR2DS1 clustering in NK cells fixed in suspension. For this, cells were suspended in 500 µl of 4% PFA/PBS and incubated for 30 min at RT, spun down, washed three times in PBS and blocked in 4% BSA/PBS for 20 min at RT. Cells were then incubated in 0.2% BSA/PBS in the presence of 5 µg/ml anti-KIR2DL/S1 mAb (clone EB6) conjugated with Atto 488, for 60 min at RT, washed 5 times with 1 ml of PBS, added to chambered slides pre-coated with PLL and allowed to settle for 5 min before imaging.

Microscopy

Single-molecule localization microscopy images were acquired on a Leica SR GSD microscope using an 160× oil immersion total internal reflection fluorescence (TIRF) objective (NA 1.43), and fluorescence was collected on an electron-multiplying charge-coupled device (EMCCD) camera (Andor iXon DU897E-C50-#BV). During acquisition, cells were illuminated in TIRF mode with 488 nm and 642 nm lasers. Up to 20,000 frames were acquired with a camera integration time of 15 ms.

STED images were acquired on Leica TCS SP8 STED CW microscope with a 100× oil immersion objective (1.40 NA). STED of AF488 and AF532 dyes was achieved using 592 nm continuous-wave fibre laser and for STED of AF568 a 660 nm continuous-wave fibre laser was used. Time-gated detection with a gate of 0.9–6.0 ms was applied. To exclude cross-excitation of fluorophores in two-channel imaging, we acquired control images with only one of the two used laser lines (495 nm or 532/575 nm) active. To control for possible anti-Stokes excitation of AF532 by the 592 nm depletion laser, for each STED image of AF532 we took an additional image with only the depletion laser active and subtracted this image from its respective STED image. To ensure the specificity of the immuno-staining, all images were compared to images of respective cells stained with an isotype-matched Ab.

Image analysis.

For GSD image reconstruction using Thunderstorm software, each raw image frame was filtered using a wavelet transform (Izeddin et al., 2012) followed by calculation of approximate molecular positions from local intensity maxima. Sub-pixel localization of molecules was performed by fitting a symmetric two-dimensional Gaussian point-spread function models using a maximum likelihood estimation fitting method (Thompson et al., 2002). Poorly-localized molecular events were discarded by filtering events with intensity < 400 photons, sigma > 9 and uncertainty ≤ 50 nm. During the course of an image acquisition, the sample may undergo drift. Lateral drift was corrected using a cross-correlation method in Thunderstorm. To correct for multiple localizations originating from the same molecule, each event with subsequent localizations appearing nearby in space (within 30 nm) and time (using an off-gap of 25 frames) were combined and counted as one molecule. Merging parameters have to be selected to balance the desired merging of re-blinking events against inadvertently merging two truly discrete emitters that happen to be nearby (e.g. because they are in a real cluster). Here, parameters for merging events were selected by preparing a dilute solution of fluorophore-labeled antibody adsorbed onto glass and assessing the spatial and temporal scale over which re-blinking occurred when imaged by GSD microscopy. It remains possible that the merging parameters used here impose a systematic error in the precise counting of molecules and data are best interpreted comparatively. The table of event localizations was exported as a text file and raster images saved by rendering each localized event as a normalized Gaussian function. For quantitative analysis, between 1 and 5 non-overlapping 3 × 3 µm regions were selected for each cell from which data was then averaged, giving a representative result for that cell. Spatial pattern analysis using Ripley's *K* function was performed with SpPack (Perry, 2004), an add-in for Microsoft Excel.

Quantitative color-scale cluster maps based on univariate Getis and Franklin's local point pattern analysis method were generated using a custom MATLAB (Mathworks) with a sampling radius of 30 nm. The sampling radius of 30 nm for mapping clusters was chosen in an empirical way by analysing the same data set using several different radii and selecting the value which allowed clusters to be identified in the binary maps most accurately. Two-dimensional pseudo-color heat-maps were created by interpolating a surface plot with L(30) as the z-axis on a grid of resolution 5 nm. Binary maps were generated by overlaying a disc element of 25 nm radius around all point localizations with L(30) above a threshold value, L(30) ≥ 65. The threshold value was chosen by comparison of distribution of L(30) values between localizations in experimental regions and randomized regions of the same area and number of molecules. Number of clusters, cluster size and other cluster parameters were measured from the binary cluster maps using ImageJ.

Signaling molecules-derived fluorescence intensity was measured in raw STED images. For other measurements, STED images were deconvolved in Huygens Professional 10.1 software (Scientific Volume Imaging) using CMLE algorithm. For nanocluster measurements, STED images were converted into binary maps by applying an automatic triangle threshold using ImageJ to regions-of-interest containing a single cell. To avoid false detections caused by noise fluctuations or unbound labeled antibodies, objects with an area smaller than 1,000 nm^2 were excluded from further analysis. Analogical binary maps of clusters identified in two-channel STED images were used for calculations of distances between KIR2DS1 and DAP12 cluster centroids using a custom MATLAB script.

To assess protein co-localization we calculated thresholded Pearson's correlation coefficients in deconvolved multi-channel STED images using an ImageJ plug-in Coloc 2 (written by Daniel J White, Tom Kazimiers and Johannes Schindelin; available on-line), which employs the Costes auto-threshold.

To identify clusters of signaling molecules overlapping or in contact with KIR clusters and residing within their close proximity (i.e. partially overlapping or remaining in contact at the edges) we converted STED images into binary maps as described above and identified x-y coordinates of cluster-centroids using the particle analysis function in ImageJ. The area and integrated fluorescence intensity of each detected cluster were also measured.

A custom MATLAB script was used to search a circular area around the centroid of each KIR2DL/S1 cluster for the presence of DAP12/SHP-1/ZAP-70 clusters. For each KIR cluster, the search radius was the sum of that cluster's own radius (calculated from the cluster area and assuming circularity) and the 75th-percentile value for cluster radii of the cognate signaling molecule (for DAP12 78 nm in unstimulated and 80 nm in activated cells; for total SHP-1 122 nm in 103 unstimulated and 114 nm in activated cells; for pY536 SHP-1 84 nm; for total ZAP-70 99 nm in resting and 126 nm in activated cells; for pY319 ZAP-70 90 nm). The distribution of those KIR2DL/S1 cluster areas with at least one signaling molecule cluster nearby was compared against the distribution of all KIR cluster areas by dividing them according to their size into the three bins. The bins corresponded to tertiles, i.e. each bin contained one third of the total number of KIR clusters detected in a particular cell.

To assess whether covering a larger area by bigger clusters could account for these clusters forming more contacts with signaling molecules, data were compared against randomised equivalents. Here, the cluster segmentation data (comprising a list of cluster descriptors including centroid coordinates and the pixels comprising each segmented cluster) for the signaling molecule channel image were randomly assigned new centroid coordinates within a region of interest delineating the cell boundary. The pixel intensity values for each cluster were also repositioned about the new centroid location and the analysis (using the original cluster segmentation data for the KIR channel) was performed. In this way, signaling molecule clusters were disconnected from their original spatial locations (while retaining their original shape and intensity properties) and a measurement of a randomised, spatially unrelated co-clustering of KIR and signaling molecules could be obtained.

To assess relative efficiency of signaling proteins recruitment and phosphorylation within clusters of certain sizes, the median integrated fluorescence intensity of DAP12/SHP-1/ZAP-70 clusters was plotted against area bins of KIR2DL/S1 clusters. Only KIR2DL/S1 clusters in contact with at least one cluster of a respective molecule were included in this analysis.

Supplemental References

Izeddin, I., Boulanger, J., Racine, V., Specht, C. G., Kechkar, A., Nair, D., Triller, A., Choquet, D., Dahan, M. & Sibarita, J. B. 2012. Wavelet analysis for single molecule localization microscopy. *Opt Express,* 20**,** 2081-95.

Perry, G. L. W. 2004. SpPack: spatial point pattern analysis in Excel using Visual Basic for Applications (VBA). *Environmental Modelling & Software,* 19**,** 559-569.

Thompson, R. E., Larson, D. R. & Webb, W. W. 2002. Precise nanometer localization analysis for individual fluorescent probes. *Biophys J,* 82**,** 2775-83.
