# Supplementary material for: The Size of Activating and Inhibitory Killer Ig-like Receptor Nanoclusters Is Controlled by the Transmembrane Sequence and Affects Signaling
Source: Cell Rep. 2016 May 19;15(9):1957–72. doi: 10.1016/j.celrep.2016.04.075 (PMC4893158; doi:10.1016/j.celrep.2016.04.075)
Supplement: Document S2. Article plus Supplemental Information [file mmc2.pdf]

Cell Reports

Article

The Size of Activating and Inhibitory Killer Ig-like Receptor Nanoclusters Is Controlled by the Transmembrane Sequence and Affects Signaling

Graphical Abstract

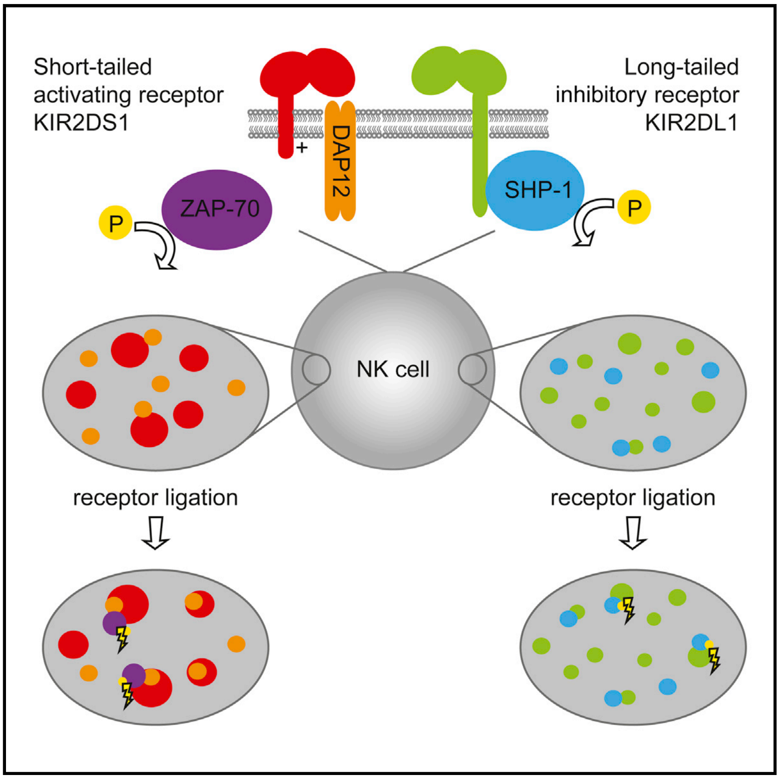

Highlights

- Activating and inhibitory NK cell receptors have a distinct nanoscale organization
- The transmembrane sequence of KIR controls their nanoscale organization
- Nanoclusters of KIR2DS1 and its adaptor are juxtaposed but mix upon activation
- Phosphorylation of ZAP-70 or SHP-1 is favored in larger receptor nanoclusters

Authors

Anna Oszmiana, David J. Williamson, Shaun-Paul Cordoba, David J. Morgan, Philippa R. Kennedy, Kevin Stacey, Daniel M. Davis

Correspondence

daniel.davis@manchester.ac.uk

In Brief

Oszmiana et al. use different super-resolution microscopy techniques to compare the nanoscale organization of paired activating and inhibitory receptors at the human NK cell surface. They show that the size of receptor nanoclusters is controlled by the transmembrane sequence and establish that nanocluster size affects the strength of receptor signaling.

CrossMark

Oszmiana et al., 2016, Cell Reports *15*, 1957–1972
May 31, 2016 © 2016 The Authors.
http://dx.doi.org/10.1016/j.celrep.2016.04.075

CellPress

OPEN ACCESS
CellPress

The Size of Activating and Inhibitory Killer Ig-like Receptor Nanoclusters Is Controlled by the Transmembrane Sequence and Affects Signaling

Anna Oszmiana,[1] David J. Williamson,[1] Shaun-Paul Cordoba,[2] David J. Morgan,[1] Philippa R. Kennedy,[1] Kevin Stacey,[1] and Daniel M. Davis[1,*]
[1]Manchester Collaborative Centre for Inflammation Research, University of Manchester, 46 Grafton Street, Manchester M13 9NT, UK
[2]Division of Cell and Molecular Biology, Sir Alexander Fleming Building, Imperial College London, London SW7 2AZ, UK
*Correspondence: daniel.davis@manchester.ac.uk
http://dx.doi.org/10.1016/j.celrep.2016.04.075

SUMMARY

Super-resolution microscopy has revealed that immune cell receptors are organized in nanoscale clusters at cell surfaces and immune synapses. However, mechanisms and functions for this nanoscale organization remain unclear. Here, we used super-resolution microscopy to compare the surface organization of paired killer Ig-like receptors (KIR), KIR2DL1 and KIR2DS1, on human primary natural killer cells and cell lines. Activating KIR2DS1 assembled in clusters two-fold larger than its inhibitory counterpart KIR2DL1. Site-directed mutagenesis established that the size of nanoclusters is controlled by transmembrane amino acid 233, a lysine in KIR2DS1. Super-resolution microscopy also revealed two ways in which the nanoscale clustering of KIR affects signaling. First, KIR2DS1 and DAP12 nanoclusters are juxtaposed in the resting cell state but coalesce upon receptor ligation. Second, quantitative super-resolution microscopy revealed that phosphorylation of the kinase ZAP-70 or phosphatase SHP-1 is favored in larger KIR nanoclusters. Thus, the size of KIR nanoclusters depends on the transmembrane sequence and affects downstream signaling.

INTRODUCTION

Natural killer (NK) cells are part of our defense against cancer and viral infections and are of medical importance in cancer immunotherapy and bone marrow transplantation (Vivier et al., 2012; Davis, 2014; Della Chiesa et al., 2014; Foley et al., 2014). Their activity depends on the balance of signals from germ-line encoded activating and inhibitory receptors. Activating receptors include NKG2D, which recognizes stress-inducible tumor ligands such as MICA, and the Fc receptor CD16, which mediates antibody-dependent cellular cytotoxicity. Inhibitory receptors that bind self-major histocompatibility complex class I proteins protect healthy cells from NK cell attack and include killer immunoglobulin (Ig)-like receptors (KIR). Interestingly, the KIR family also includes activating receptors, which share ligand specificity with their inhibitory counterparts due to structural homology of extracellular domains (Ivarsson et al., 2014; Biassoni et al., 1997). One example of such a pairing are receptors KIR2DL1 and KIR2DS1, which bind to human leukocyte antigen (HLA) proteins from the C2 group (Stewart et al., 2005; Sivori et al., 2011). KIR3DS1, in combination with its HLA ligand, is associated with delayed progression to AIDS and protection against hepatitis C infection (Khakoo et al., 2004; Alter et al., 2007, 2011; Carr et al., 2007; Alter et al., 2011). Also, *KIR2DS1* in the telomeric region of the *KIR B* haplotype was shown to have a protective effect against complications in pregnancy (Xiong et al., 2013; Hiby et al., 2010).

Functional divergence of KIR2DL1 and KIR2DS1 is conferred by differences in transmembrane and intracellular sequences. The longer cytoplasmic tail of KIR2DL1 contains two immunoreceptor tyrosine-based inhibition motifs (ITIMs), which recruit the tyrosine phosphatase SHP-1 (Fry et al., 1996; Burshtyn et al., 1996) to block the membrane proximal activating signals (Stebbins et al., 2003). KIR2DS1 lacks ITIMs and instead associates with DNAX activation protein 12 (DAP12), an adaptor protein containing an immunoreceptor tyrosine-based activation motif (ITAM) (Lanier et al., 1998). Cytolysis, cytokine production, and cellular proliferation are triggered in NK cells expressing KIR2DS1, but not KIR2DL1, upon interaction with HLA-C2^{+} target cells (Sivori et al., 2011; Moretta et al., 1995; Mandelboim et al., 1998; Rose et al., 2009). In NK cells expressing both activating and inhibitory paired receptors, effector functions are often inhibited (Moretta et al., 1995; Valés-Gómez et al., 1998; Watzl et al., 2000).

The nanoscale organization of NK cell receptors changes with the state of activation of the cell. Specifically, clusters of KIR2DL1 become smaller upon ligation of activating receptor NKG2D, increasing the local density of inhibitory receptors (Pageon et al., 2013). In murine NK cells, fluorescence correlation spectroscopy revealed that confinement of activating receptors at the plasma membrane changes upon NK cell education (Guia et al., 2011). However, a major unknown is whether the nanometer-scale organization of NK cell receptors affects signaling. Here, we compare the nanometer-scale organization of activating and inhibitory KIR2DS1 and KIR2DL1 at the surface of NK cells. We report that these two receptors are organized

This is an open access article under the CC BY license (http://creativecommons.org/licenses/by/4.0/).

differently, determined by their transmembrane sequences. Importantly, we also establish that the size of receptor nanoclusters affects signaling.

RESULTS

Distinct Nanoscale Organization of KIR2DL1 and KIR2DS1 in NKL Cells

To compare the organization of inhibitory KIR2DL1 and activating KIR2DS1, the human cell line NKL was stably transduced to express each receptor fused to a hemagglutinin (HA) tag at the C terminus (NKL/KIR2DL1-HA and NKL/KIR2DS1-HA; Figure S1). Tagged receptors retained functionality, as ligation of KIR2DL1-HA inhibited the formation of a dense ring of peripheral F-actin at the contact interface, and the secretion of interferon (IFN)-γ, in cells activated via NKG2D (Figures S1D and S1G). In contrast, ligation of KIR2DS1-HA triggered the formation of peripheral actin rings, as well as IFN-γ secretion (Figures S1E and S1H).

The nanoscale organization of KIR2DL1 and KIR2DS1 at the cell surface was compared using ground state depletion microscopy followed by individual molecule return (GSDIM). For this, NKL/KIR2DL1-HA and NKL/KIR2DS1-HA cells were plated on poly-L-lysine-coated slides, fixed and stained with a directly labeled anti-KIR2DL/S1 monoclonal antibody (mAb) EB6 (Figure 1). Visual inspection of images (Figure 1A), as well as Ripley's *K* function-based analysis (Ripley, 1977) (Figure 1B), showed that both receptors constitutively assembled in nanometer-scale clusters, but the degree and radial scale of clustering were larger for KIR2DS1. We then created quantitative maps of clustering based on univariate Getis and Franklin's local point pattern analysis (Getis and Franklin, 1987; Williamson et al., 2011; Pageon et al., 2013). Randomized data with the same density of localizations were compared with the experimental data to establish an appropriate threshold for the identification of clusters. Quantifications of this type provide information only on the events detected and are sensitive to changes in parameters such as search radius. In addition, both the hardware and the methods of analysis for super-resolution microscopy data are being developed at a rapid rate. An explicit example of the impact of this is that we report a lower fraction of KIR2DL1 in clusters compared to our earlier research (Pageon et al., 2013). Thus, as in nearly all super-resolution microscopy reports, precise values are indicative rather than definitive and analysis is effective in revealing relative differences between different receptors or conditions.

Our analysis here showed that KIR2DS1 formed clusters of larger area (with a median of 14,800 nm^2) compared to KIR2DL1 (median 6,600 nm^2; Figure 1C). The majority of KIR2DL1 clusters (61% ± 9%) had an area $\leq$5,000 nm^2 (which corresponds to diameter $\leq$80 nm), and only 8% ± 6% were bigger than 15,000 nm^2 (diameter $\geq$138 nm). In contrast, 31% ± 5% of KIR2DS1 clusters were over 15,000 nm^2 (Figure 1D). The density of clusters was higher for KIR2DL1 compared to KIR2DS1 ($p < 0.0001$, Mann-Whitney test; Figure 1E). In addition, clusters of KIR2DS1 were more tightly packed, 7.1-fold denser than the average density within the membrane (Figure 1F) and contained a higher fraction of total molecules (median 40%, Figure 1G), whereas KIR2DL1 clusters were 4.4-fold denser than the membrane overall and contained a lower proportion of molecules (median 25%).

The total density of detected events for both receptors was comparable ($p = 0.6913$; Figure 1H), indicating that differences in organization were not due to variable numbers of events detected. Also, cross-correlation of cluster size and overall molecular density showed that in individual cells expressing comparable levels of each of the two receptors, KIR2DS1 consistently formed bigger clusters than KIR2DL1 (Figure 1I). Together, these data indicate that nanoclusters of KIR2DS1 are bigger and more densely packed, while clusters of KIR2DL1 are more numerous and smaller.

Several control experiments were carried out as follows. First, because these measurements crucially depend on the efficiency of labeling, we stained the same cells with a directly labeled mAb targeting the HA tag. When visualized this way, clusters of KIR2DS1 were also bigger than clusters of KIR2DL1 (Figures S2A and S2B). To check if the organization of KIR was altered by contact with poly-L-lysine-coated slides, we also imaged cells that were fixed and stained in suspension prior to plating upon glass slides. Again, we observed bigger clusters for KIR2DS1 (Figures S2H and S2I). Finally, we imaged the endogenous interleukin-2 receptor subunit α (IL-2Rα), which assembled into nanoclusters with identical characteristics in both transfectants showing that the presence of KIR2DS1 or KIR2DL1 did not alter the nanoclustering of the cell surface in general (Figures S3A–S3G). Together, these experiments further establish the distinct nanoscale organization of KIR2DL1 and KIR2DS1 at the surface of transfected cell lines.

Nanoclusters of KIR2DL1 and KIR2DS1 Are Spatially Segregated

Earlier research suggested a role for co-localization of activating and inhibitory receptors on a micrometer-scale in the integration of their signals (Köhler et al., 2010). To characterize the nanoscale organization of KIR2DL1 and KIR2DS1 when co-expressed, NKL cells were transduced to express the two receptors fused to either HA or FLAG peptide tags in both combinations, i.e., NKL/KIR2DL1-HA/KIR2DS1-FLAG as well as NKL/KIR2DL1-FLAG/KIR2DS1-HA, and confirmed as functional (Figures S4A–S4C). Cells were plated on poly-L-lysine-coated slides, fixed, stained with anti-HA mAb conjugated to Alexa Fluor 488 (AF488) and anti-FLAG mAb conjugated to AF532 for imaging by stimulated emission depletion (STED) microscopy. As a positive control for co-localization, the same protein, KIR2DS1-HA, was stained with two different mAb, one against the HA tag (labeled with AF488) and another mAb against the extracellular portion of KIR2DL/S1 (labeled with AF532). To quantify co-localization for KIR2DS1 and KIR2DL1, a Pearson correlation coefficient, which ranges between 1.0 (complete co-localization) and −1.0 (complete segregation), was calculated for each cell. The median correlation coefficient for KIR2DS1 and KIR2DL1 was −0.17 in NKL/KIR2DL1-FLAG/KIR2DS1-HA and −0.22 in NKL/KIR2DL1-HA/KIR2DS1-FLAG cells, as compared to 0.77 in the positive control (Figures S4D and S4E). Thus, KIR2DS1 and KIR2DL1 are constitutively localized within spatially segregated nanoclusters.

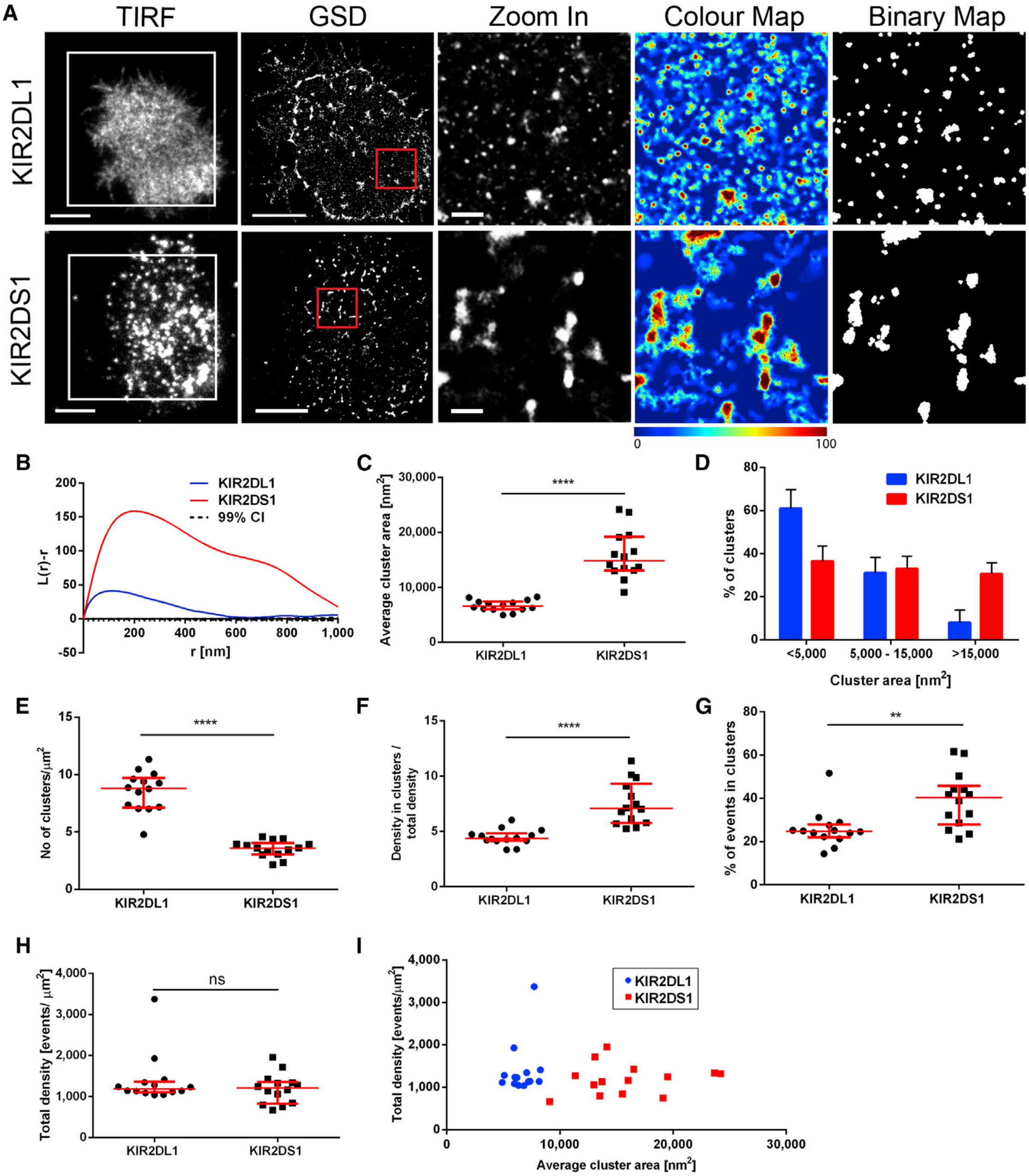

Figure 1. Distinct Organization of KIR2DL1 and KIR2DS1 at the Surface of NKL Depicted with GSD Microscopy

(A–I) NKL/KIR2DL1-HA and NKL/KIR2DS1-HA cells on PLL-coated slides were stained with anti-KIR2DL/S1 mAb labeled with AF647. (A) Representative TIRF and GSD images of NKL/KIR2DL1-HA and NKL/KIR2DS1-HA cells. Scale bars represent 5 μm. The 3 × 3 μm regions (red boxes in GSD images) are magnified and shown with corresponding color cluster maps and binary maps (scale bars represent 500 nm). Colors correspond to the L(30) values. (B) Ripley's *K* function of the events in the selected regions (red boxes) shown in (A). $L(r) - r$ represents the degree of clustering at different spatial scales relative to simulated random distributions, indicated by the 99% confidence intervals (CI); r is the radial scale. (C–H) Quantitative analysis of KIR2DL1 and KIR2DS1 clustering in NKL/KIR2DL1-HA and NKL/KIR2DS1-HA cells: average cluster area (C), size distribution of clusters (D), clusters per square micrometer (E), ratio between density of events in clusters to average membrane density (F), percentage of events in clusters (G), and overall density of detected events (H). (I) Total density of events plotted against average cluster area measured in individual cells. (C) and (E)–(I) Each symbol represents the mean from several regions within one cell. Horizontal bars and errors represent the medians and interquartile range. (D) Bars and errors represent means and SD. Data are from 14 cells per receptor from two independent experiments. ns, non-significant. **$p < 0.01$, ****$p < 0.0001$, Mann-Whitney test.

See also Figures S1, S2, S3, S4, and S5.

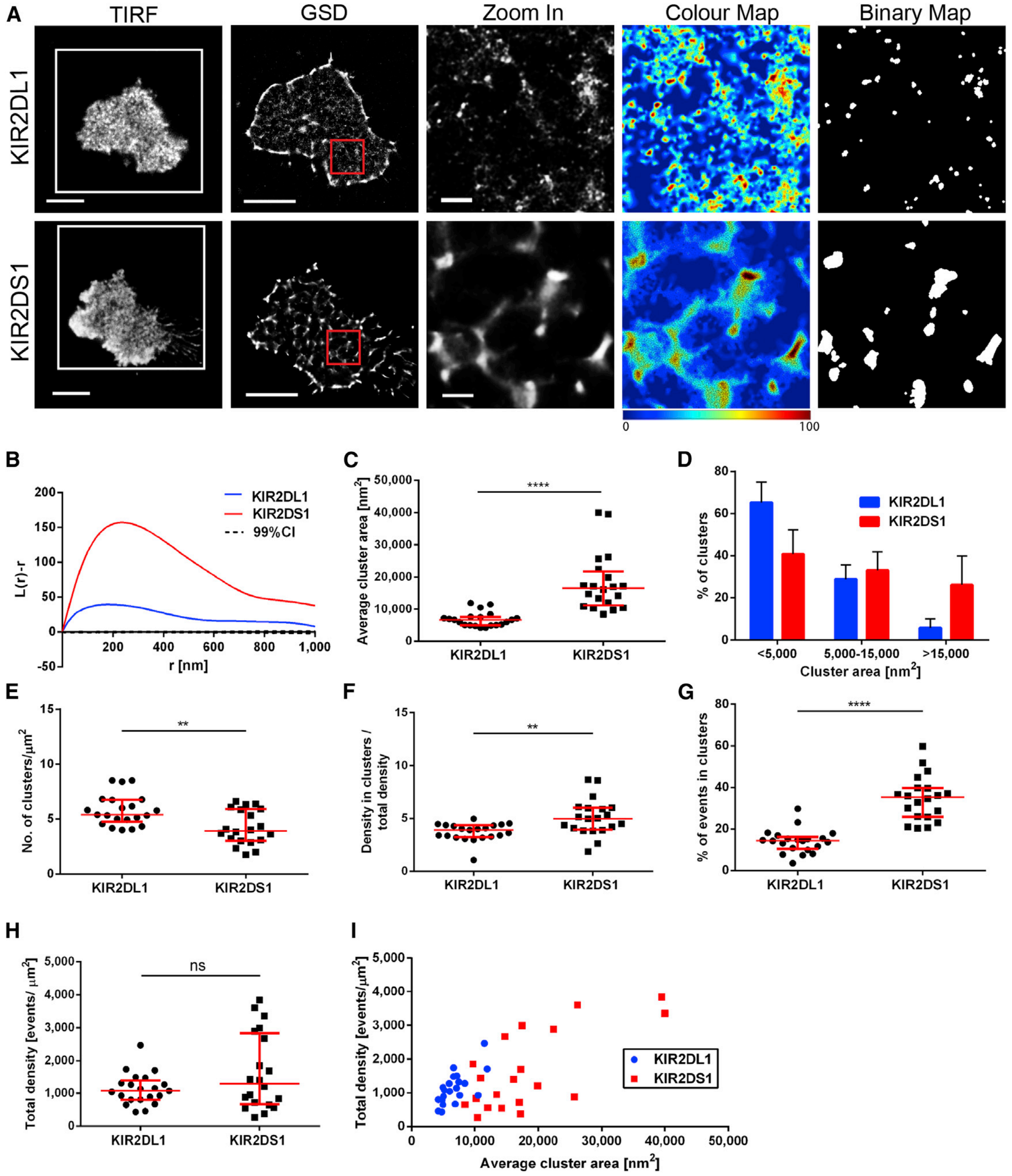

Figure 2. KIR2DL1 and KIR2DS1 Are Organized Differently at the Surface of Primary Human NK Clones

(A) Representative TIRF and GSD images of 2DS1$^-$/2DL1$^+$ and 2DS1$^+$/2DL1$^-$ NK clones on PLL-coated slides stained with anti-KIR2DL/S1 mAb labeled with Atto 488. Scale bars represent 5 μm. The 3 × 3 μm regions (red boxes in GSD images) are magnified and shown with corresponding color cluster maps and binary maps (scale bars represent 500 nm).

(B) Ripley's *K* function of the events in the selected regions (red boxes) shown in (A). $L(r) - r$ represents the degree of clustering at different spatial scales relative to simulated random distributions, indicated by the 99% confidence intervals (CI); r is the radial scale.

(C–H) Quantitative analysis of KIR2DL1 and KIR2DS1 clustering in 2DS1$^-$/2DL1$^+$ and 2DS1$^+$/2DL1$^-$ NK clones, respectively: average cluster area (C), size distribution of clusters (D), number of clusters per square micrometer (E), ratio between density of events in clusters to overall membrane density (F), percentage of events in clusters (G), and overall density of detected events (H).

(I) Total density of events plotted against average cluster area in individual cells for KIR2DL1 and KIR2DS1.

Each symbol represents the mean from several regions within one cell (C and E–I). Horizontal bars and errors represent the medians and interquartile range. Bars and errors represent means and SD (D). Data are from 21 (KIR2DL1) and 20 (KIR2DS1) cells each from five clones derived from two donors. ns, non-significant. $^{**}p < 0.01$, $^{****}p < 0.0001$, Mann-Whitney test. See also Figure S5.

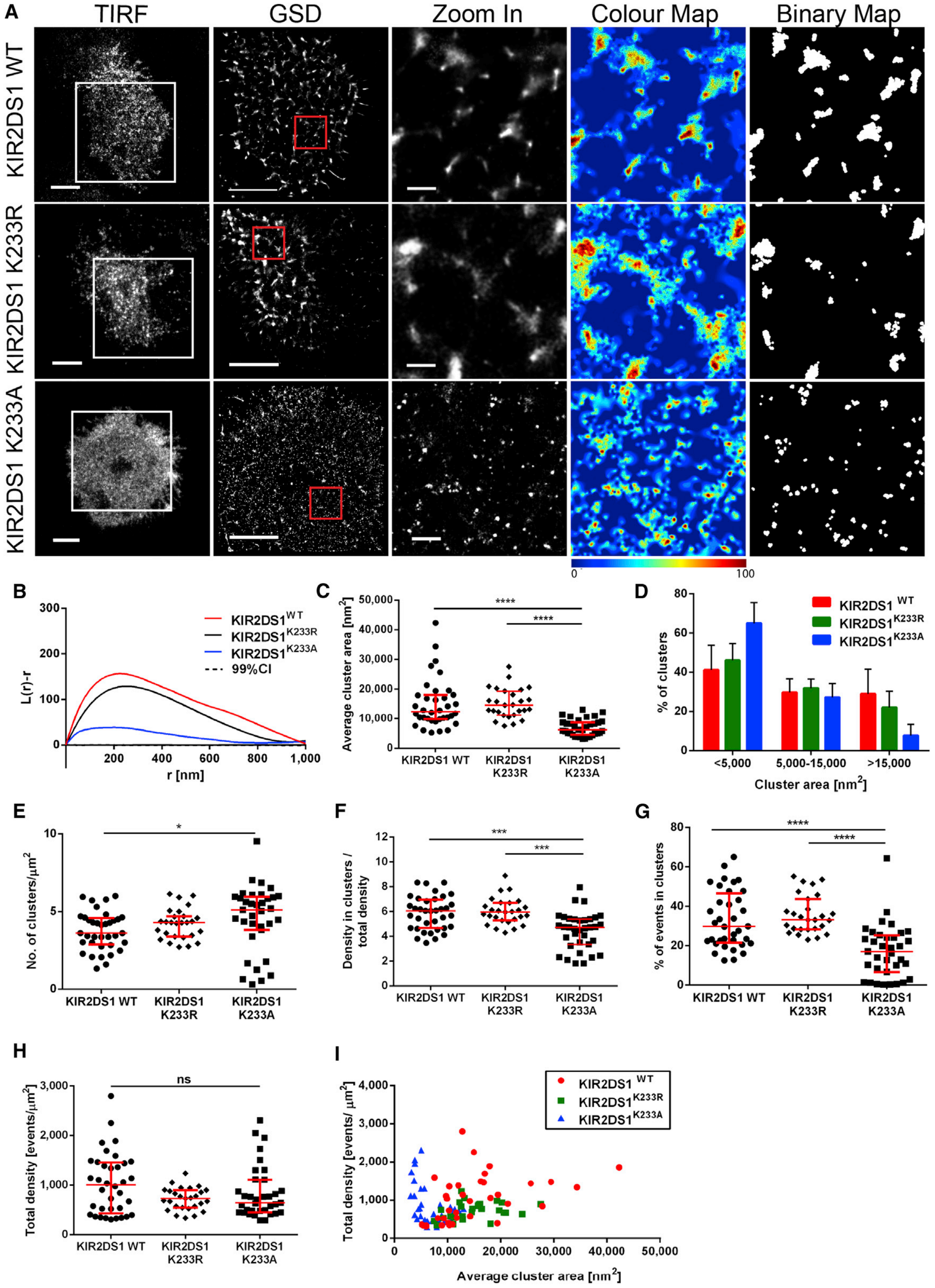

Figure 3. Substitution of the Transmembrane Lysine in KIR2DS1 Changes Its Nanometer-Scale Organization

(A) Representative TIRF and GSD images of NKL/KIR2DS1-HA, NKL/KIR2DS1^{K233R}, and NKL/KIR2DS1^{K233A} cells incubated on PLL-coated slides and stained with anti-HA mAb labeled with AF488. Scale bars represent 5 μm. The 3 × 3 μm regions (red boxes in GSD images) are magnified and shown with corresponding color maps and binary maps (scale bars represent 500 nm).

(legend continued on next page)

Distinct Nanoclustering of KIR2DL1 and KIR2DS1 in Primary Human NK Clones

We next compared the nanoscale organization of KIR2DL1 and KIR2DS1 in human NK clones derived from peripheral blood. Clones expressing either KIR2DL1 or KIR2DS1 only, were identified using two mAb that compete for binding. mAb EB6 binds to NK cells expressing either one or both of the receptors, whereas mAb 143211 ligates only KIR2DL1 which allows discrimination between 2DS1^{+}/2DL1^{-}, 2DS1^{-}/2DL1^{+} and 2DS1^{+}/2DL1^{+} clones (Fauriat et al., 2010). Clones phenotyped as 2DS1^{+}/2DL1^{-} and 2DS1^{-}/2DL1^{+} were plated on poly-L-lysine-coated slides, fixed, stained with fluorescently labeled anti-KIR2DL/S1 mAb and imaged by GSDIM. As in transfectants, KIR2DL1 and KIR2DS1 assembled in nanoclusters with distinct characteristics (Figure 2A), demonstrated by $L(r) - r$ function plots (Figure 2B) and quantitative analysis. Clusters of KIR2DS1 had significantly larger area (with a median of 16,600 nm^2), than KIR2DL1 clusters (median 6,700 nm^2; Figure 2C). In addition, only 6% ± 4% of KIR2DL1 clusters had area above 15,000 nm^2, while such large clusters of KIR2DS1 were 4.5 times more frequent (26% ± 14%; Figure 2D). The density of KIR2DL1 clusters on primary cells was higher than for KIR2DS1 (p = 0.0036, Mann-Whitney test; Figure 2E). Clusters of KIR2DS1 were more tightly packed than KIR2DL1 (p = 0.0024; Figure 2F) and contained a higher fraction of the molecules ($p < 0.0001$, Figure 2G). The total density of receptors in 2DS1^{+}/2DL1^{-} and 2DS1^{-}/2DL1^{+} clones was similar (Figures 2H and 2I), implying that the differences in nanoscale organization of KIR2DL1 and KIR2DS1 cannot be accounted for by differences in expression level. Therefore, endogenously expressed KIR2DS1 and KIR2DL1 are distinctly organized at the surface of resting primary human NK cells.

Each super-resolution microscopy technique has caveats (Pageon et al., 2012). Therefore, we also compared the organization of KIR2DL1 and KIR2DS1 in primary cells and transfectants of NKL using STED microscopy. STED images of NKL/KIR2DL1-HA and NKL/KIR2DS1-HA cells stained with anti-HA mAb (Figures S5A and S5B) and 2DS1^{+}/2DL1^{-} and 2DS1^{-}/2DL1^{+} NK clones stained with anti-KIR2DL/S1 mAb (Figures S5C and S5D) were deconvolved and thresholded to create binary maps of nanoclusters. Measured this way, KIR2DS1 clusters had larger area than KIR2DL1 in both NKL ($p < 0.0001$; Mann-Whitney test; Figure S5B) and primary NK cells ($p < 0.0001$; Figure S5D).

Nanoscale Clustering of KIR2DS1 and KIR2DL1 Is Controlled by the Transmembrane Sequence

KIR2DL1 and KIR2DS1 are highly homologous. The two alleles used here differ by only seven amino acids in their extracellular domain (representing 97% homology within this domain), with the bulk of the differences being in the transmembrane and cytoplasmic domains. Thus, it is not immediately obvious why these proteins organize differently at cell surfaces. To establish this, we tested the importance of the positively charged lysine at position 233 within transmembrane region of KIR2DS1, which facilitates interactions with DAP12 (Lanier et al., 1998; Bottino et al., 2000; Feng et al., 2006). For this, NKL was transduced to express mutant versions of KIR2DS1-HA where the transmembrane lysine had been substituted with neutral-charged alanine (KIR2DS1^{K233A}) or positively charged arginine (KIR2DS1^{K233R}). Cells were plated on poly-L-lysine-coated slides, fixed, stained with anti-HA mAb and imaged by GSDIM. Visual inspection (Figure 3A), $L(r) - r$ plots (Figure 3B) and quantitative comparison of clusters establish that substitution of lysine 233 of KIR2DS1 with alanine, but not arginine, affected its organization.

The area of KIR2DS1^{K233A} clusters was significantly lower than wild-type KIR2DS1 ($p < 0.0001$, Kruskal-Wallis test by ranks with Dunn's post-test; Figure 3C) and had a median of 6,100 nm^2. The majority of KIR2DS1^{K233A} clusters (65% ± 11%) had area $\leq$5,000 nm^2 (as opposed to 41% ± 13% for wild-type) and KIR2DS1^{K233A} clusters larger than 15,000 nm^2 were rarely detected (7.5% ± 6% of all clusters, as opposed to 29% ± 13% for wild-type, Figure 3D). Density of KIR2DS1^{K233A} clusters was slightly increased compared to wild-type KIR2DS1 (p = 0.0145, Figure 3E). On the other hand, the size and density of KIR2DS1^{K233R} clusters was indistinguishable from wild-type receptor. Clusters of KIR2DS1^{K233A} were less densely packed and contained a lower proportion of molecules than clusters of wild-type KIR2DS1 and KIR2DS1^{K233R} (Figures 3F and 3G). The total density of events in cells imaged was similar (Figure 3H) and differences between cluster sizes of different receptors were consistent across a range of molecular densities (Figure 3I). Overall, KIR2DS1 in which the transmembrane lysine was replaced by alanine was organized similarly to KIR2DL1 across many parameters (*cf*. Figure 3 with Figure 1), while substituting the lysine for another charged amino acid, arginine, had little, if any, effect.

We next assessed whether, reciprocally, the insertion of a charged residue in the transmembrane sequence of inhibitory KIR2DL1 would influence its clustering. For this, NKL was transfected to express KIR2DL1 in which neutral isoleucine at position 233 was substituted with lysine (NKL/KIR2DL1^{I233K}). To test whether tonic signaling by KIR could influence its clustering, we made a separate transfectant of NKL expressing KIR2DL1 in which the two ITIM tyrosines were substituted with alanine (NKL/KIR2DL1$^{Y281A/Y311A}$). Introduction of lysine into the transmembrane domain of KIR2DL1 had a dramatic effect on the

(B) Ripley's *K* function of the events in the selected regions (red boxes) shown in (A).
(C–H) Analysis of wild-type KIR2DS1, KIR2DS1^{K233R}, and KIR2DS1^{K233A} clustering: average cluster area (C), size distribution of clusters (D), clusters per square micrometer (E), ratio between density of events in clusters to average membrane density (F), percentage of events in clusters (G), and overall density of detected events (H).
(I) Total density of detected events plotted against average cluster area in individual cells.
(C and E–I) Each symbol represents the mean from several regions within one cell. Horizontal bars and errors represent the medians and interquartile range. (D) Bars and errors represent means and SD. Data are from 36 cells from five experiments (KIR2DS1^{WT}), 27 cells from three experiments (KIR2DS1^{K233R}), and 35 cells from four experiments (KIR2DS1^{K233A}). ns, non-significant. *p < 0.05, ***p < 0.001, ****p < 0.0001, Kruskal-Wallis test by ranks with Dunn's post-test. See also Figure S6.

OPEN
ACCESS
CellPress

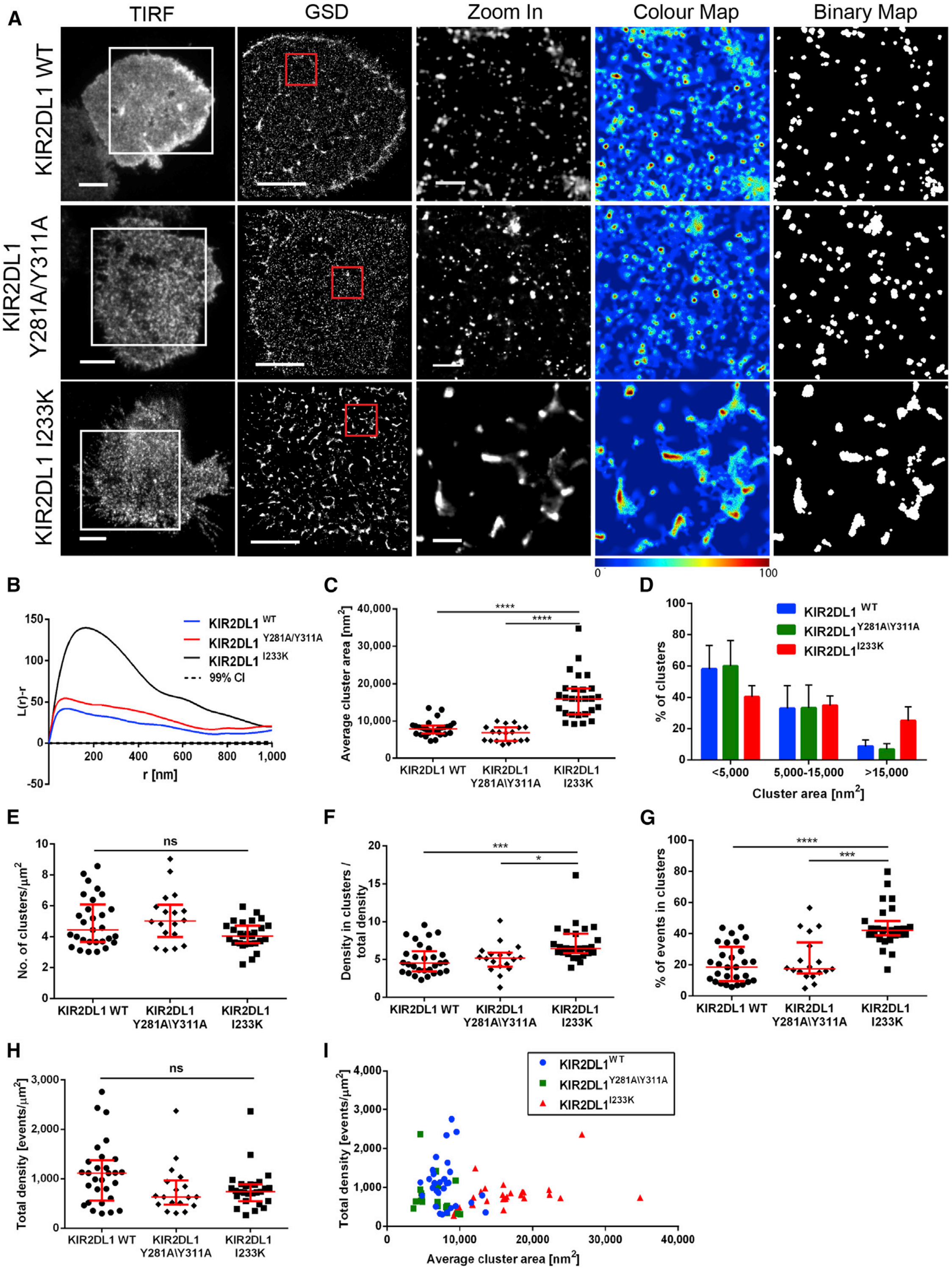

Figure 4. Substitution of the Transmembrane Isoleucine with Lysine in KIR2DL1 Changes Its Nanometer-Scale Organization

(A) Representative TIRF and GSD images of NKL/KIR2DL1-HA, NKL/KIR2DL1$^{Y281A/Y311A}$, and NKL/KIR2DL1^{I233K} cells incubated on PLL-coated slides, stained with anti-HA mAb labeled with AF488. Scale bars represent 5 μm. The 3 × 3 μm regions (red boxes in GSD images) are magnified and shown with corresponding color maps and binary maps (scale bars represent 500 nm).

(B) Ripley's *K* function of the events in the selected regions (red boxes) shown in (A).

(legend continued on next page)

protein's nanoscale organization (Figure 4A), as shown in plots of $L(r) - r$ (Figure 4B) and quantitative analysis of clusters. In contrast, replacing the signaling tyrosines had little, if any, effect on clustering. The clusters of KIR2DL1^{I233K} were significantly larger than wild-type KIR2DL1 ($p < 0.0001$) and had a median area of 16,000 nm^2 (Figure 4C). The distribution of KIR2DL1^{I233K} cluster sizes was skewed toward larger clusters (Figure 4D). We detected similar number of clusters per μm^2 for KIR2DL1^{I233K} and wild-type KIR2DL1 (Figure 4E). The relative density of events in clusters and the proportion of events localized within clusters also increased for KIR2DL1^{I233K} (Figures 4F and 4G).

The mean levels of surface expression for mutated receptors differed to some extent from wild-type proteins (Figures S6A and S6C), but several lines of evidence determine that this did not account for the differences in nanoscale clustering. First, cells imaged had comparable levels of detected molecules (Figures 3H and 4H). Second, there was no obvious correlation between the total density of molecules detected and the average cluster size (Figure 3I and 4I). Third, to directly test for any effect of expression level, cells within the population that naturally express different densities of receptors were compared. For cells with low or high levels of expression, wild-type KIR2DS1 clusters were similar in size to KIR2DS1^{K233R} but consistently larger than clusters of KIR2DS1^{K233A} (Figure S6B). Similarly, KIR2DL1^{I233K} formed bigger and denser clusters than wild-type receptor or KIR2DL1$^{Y281A/Y311A}$ in cells expressing the protein at different densities (Figure S6D).

Association with DAP12 Does Not Account for the Differences in Clustering between KIR2DS1 and KIR2DL1

The lysine in the transmembrane sequence of KIR2DS1 facilitates its interaction with DAP12 (Lanier et al., 1998; Bottino et al., 2000; Feng et al., 2006). Thus, we next tested if an association with DAP12 affects the nanoscale clustering of KIR2DS1. For this, we stably transduced the human leukemic T cell line Jurkat E6.1, which lacks DAP12 (Lanier et al., 1998), to express KIR2DL1-HA or KIR2DS1-HA. Cells were plated on poly-L-lysine-coated slides, fixed, stained with anti-HA mAb and imaged by GSDIM (Figures S3H–S3N). The organization of KIR2DL1 and KIR2DS1 at the surface of Jurkat cells closely resembled that seen in NK cells (Figure S3H). Clusters of KIR2DS1 in Jurkat T cells were significantly larger than KIR2DL1 ($p < 0.0001$, Mann-Whitney test; Figure S3I) and were less abundant ($p = 0.0001$; Figure S3J). Also, a higher fraction of KIR2DS1 molecules was found within more densely packed clusters (Figures S3K and S3L). As in NK cells, the differences in KIR2DL1 and KIR2DS1 nanoclusters sizes were consistent across a range of molecular densities (Figures S3N and S6F). Thus, although it cannot be excluded that KIR2DS1 might associate with other adaptor molecules in Jurkat T cells, the presence of DAP12 is not required for KIR2DS1 to assemble in larger clusters.

Nanoclusters of KIR2DS1 and DAP12 Mix upon Ligation of KIR2DS1

Having established that clustering of KIR2DS1 is independent of its association with DAP12, we next examined how these proteins are organized at the surface of NK cells with and without receptor ligation. For this, NKL/KIR2DS1-HA cells were plated on slides coated with anti-KIR2DL/S1 mAb or isotype-matched control mAb, and imaged by STED microscopy. Positive colocalization was indicated using KIR2DS1 labeled with anti-KIR2DS/L1 mAb conjugated to Atto 488 and anti-HA mAb followed by secondary Ab conjugated to AF568 (Figures 5A and 5B). Unexpectedly, on non-activating control-coated slides there was little direct overlap between clusters of KIR2DS1 and DAP12 (Figure 5A). This was reflected by a low Pearson correlation coefficient (median 0.22; Figure 5B). However, clusters of DAP12 tended to contact the edges of clusters of KIR2DS1 (Figure 5A). Importantly, when cells were stimulated on anti-KIR2DL/S1 mAb, the degree of mixing between nanoclusters of KIR2DS1 and DAP12 significantly increased. This was clearly seen in the overlay of images as well as by an increase in the Pearson correlation coefficient (median 0.45) (Figures 5A and 5B) and a decrease in the average distance between the KIR2DS1 and DAP12 cluster centroids, from a median of 240 nm in unstimulated cells to 153 nm upon ligation of KIR2DS1 (Figure S7A). Ligation of KIR2DS1 also led to the recruitment of DAP12 to the membrane at the contact site, as evidenced by an increase in DAP12 fluorescence intensity (Figure 5C), suggesting that at least some of the increased mixing between DAP12 and KIR2DS1 can come from increased levels of DAP12 at the membrane. Thus, ligation of KIR2DS1 triggers a closer association of receptor clusters with DAP12. In contrast, the Pearson correlation coefficient between the KIR and DAP12 clusters was significantly lower in both resting and activated NKL/KIR2DS1^{K233A} cells (Figure 5B). Clusters of DAP12 were localized significantly further from KIR2DS1^{K233A} clusters than wild-type KIR2DS1 (Figures S7A and S7B) and less DAP12 was detected at the membrane (Figure S7C) consistent with the prediction that DAP12 does not associate with this receptor.

Larger Clusters of KIR2DS1 Are More Often Associated with Clusters of DAP12

Ligation of KIR2DS1 also led to an increase in the mean area of the receptor clusters (Figure S7D). There was also a small but not significant increase in the area of DAP12 clusters (Figure S7E).

(C–H) Quantitative analysis of wild-type KIR2DL1, KIR2DL1$^{Y281A/Y311A}$, and KIR2DL1^{I233K} clustering: average cluster area (C), size distribution of clusters (D), clusters per square micrometer (E), ratio between density of events in clusters to overall membrane density (F), percentage of events in clusters (G), and overall density of detected events (H).
(I) Total density of detected events plotted against average cluster area in individual cells for wild-type KIR2DL1, KIR2DL1$^{Y281A/Y311A}$, and KIR2DL1^{I233K}.
(C and E–I) Each symbol represents the mean from several regions within one cell. Horizontal bars and errors represent the medians and interquartile range. (D) Bars and errors represent means and SD. Data are from 29 cells from five experiments (KIR2DL1^{WT}), 27 cells from four experiments (KIR2DL1^{I233K}), and 18 cells from three experiments (KIR2DL1$^{Y281A/Y311A}$). ns, non-significant. $^*p < 0.05$, $^{***}p < 0.001$, $^{****}p < 0.0001$, Kruskal-Wallis test by ranks with Dunn's post-test.
See also Figure S6.

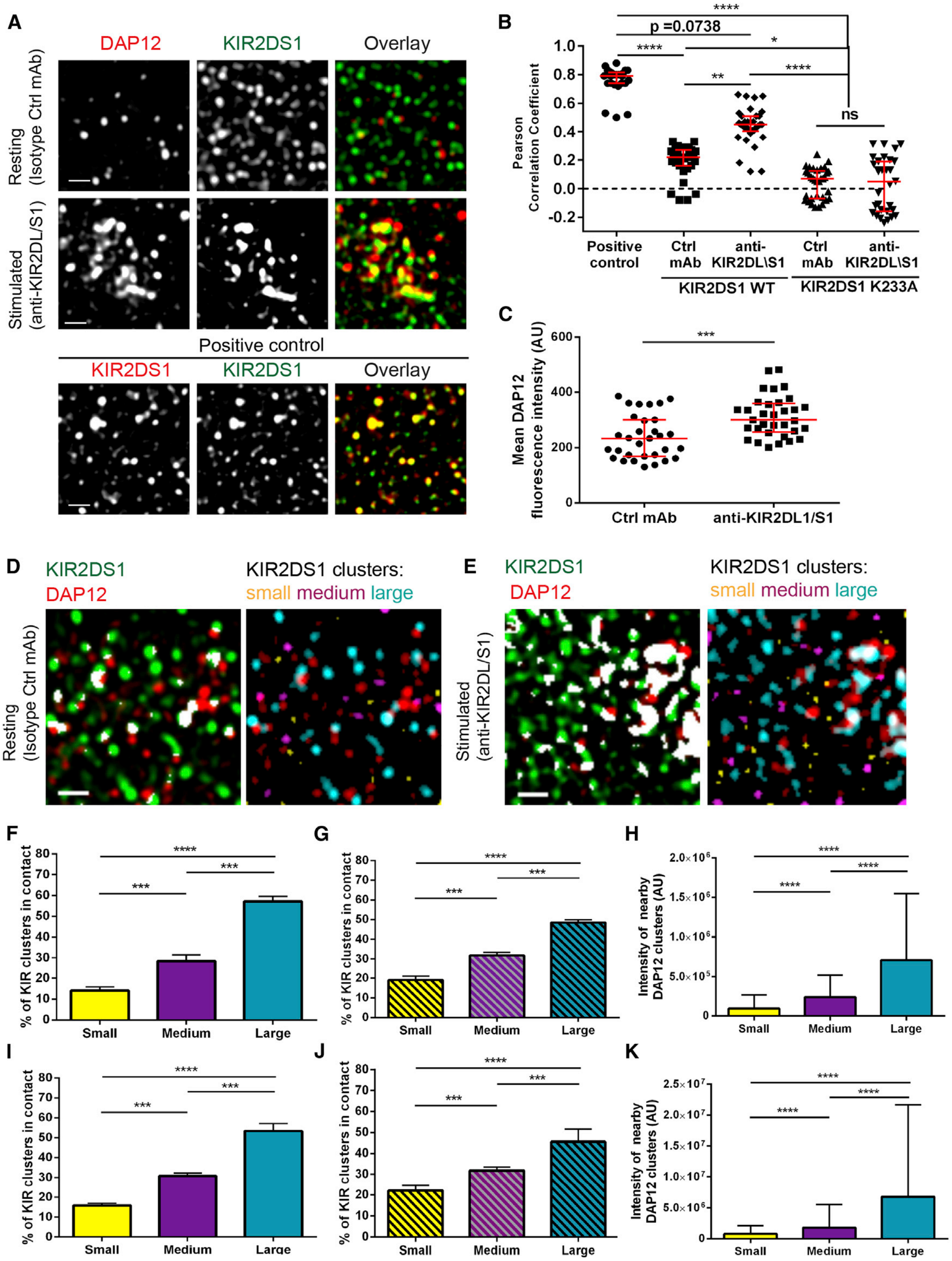

Figure 5. Interaction between Nanoclusters of KIR2DS1 and DAP12

(A) NKL/KIR2DS1-HA cells were incubated on isotype control-coated (top row) or anti-KIR2DL/S1 mAb-coated (middle row) slides, stained for KIR2DS1 (with an anti-HA mAb, AF488) and DAP12 (AF568), and imaged with STED. For a positive control (bottom row), NKL/KIR2DS1-HA was stained with anti-KIR2DL/S1 mAb (green; Atto488) and anti-HA mAb (red; AF568). Representative 3 × 3 μm regions from STED images of NKL/KIR2DS1-HA cells with channels overlaid and separated (overlapping green and red pixels are marked yellow). Scale bars represent 500 nm.

(B) Pearson correlation coefficients for KIR2DS1 or KIR2DS1^{K233A} and DAP12 in cells on isotype control-coated and anti-KIR2DL/S1 mAb-coated slides, compared to positive control.

(legend continued on next page)

Thus, we set out to investigate whether the size of KIR2DS1 clusters is important for its signaling. To test this, we exploited the inherent heterogeneity in the size of KIR nanoclusters within individual cells. Standard methods to quantify protein co-localization only take into account fluorescence that is directly overlapping, e.g., within one pixel, and do not report lateral connections between nanoclusters, such as those apparent in our images. Therefore, to quantify the association between clusters, we screened a circular area around the centroid of each KIR2DS1 cluster for the presence of DAP12 clusters. KIR2DS1 clusters that were associated with DAP12 were split into tertiles according to their area (designated small, medium, or large; Figures 5D–5K). This demonstrated that both in resting cells and upon KIR2DS1 ligation, the largest clusters of KIR2DS1 were most often found in association with DAP12 clusters, accounting for more than half of all cluster interactions (Figures 5F and 5I).

The amount of DAP12 proximal to KIR2DS1 clusters was also higher for large clusters (Figures 5H and 5K). Interestingly, a similar frequency of contacts between differently sized KIR clusters and DAP12 was calculated from imaging data altered so that the positions of DAP12 clusters were randomized within the cell area (Figures 5G and 5J). Thus, large clusters of KIR2DS1 are inherently more likely to associate with clusters of DAP12 on account of their size.

The Size of KIR2DS1 Nanoclusters Is Important for Recruitment and Phosphorylation of ZAP-70

To determine whether the size of KIR2DS1 nanoclusters also affects downstream signaling, we next imaged this receptor and the membrane-proximal kinase ZAP-70 which participates in KIR2DS1 signaling (Lanier et al., 1998), in NK cells plated on isotype-matched or anti-KIR2DL/S1 mAb-coated slides (Figure 6). Ligation of wild-type KIR2DS1, but not KIR2DS1^{K233A}, led to an increase in the amount of ZAP-70 (Figures 6A and S7F) and its activated form phosphorylated on tyrosine 319 (ZAP-70 pY319; Figures 6I and S7G) proximal to the membrane at the contact site. Many clusters of KIR2DS1 (Figures 6B and 6J, green) were not associated with clusters of ZAP-70 or pZAP-70 (red). However, most clusters of the kinase did connect with the periphery of KIR2DS1 nanoclusters (overlap highlighted in white; Figures 6B and 6J). Clusters of ZAP-70 were preferably found in contact with larger clusters of KIR2DS1 in both resting and stimulated cells (Figures 6C and 6F). Only very few clusters of ZAP-70 pY319 were detected in unstimulated cells, but in stimulated cells, clusters of phosphorylated ZAP-70 were more often found near the largest KIR clusters (Figure 6K).

Comparison with simulated datasets, in which the positions of ZAP-70 clusters were randomized, indicated that large clusters were intrinsically more likely to contact clusters of ZAP-70 or ZAP-70 pY319 (Figures 6D, 6G, and 6L). The amount of ZAP-70 and phosphorylated ZAP-70 also increased when associated with larger KIR clusters (Figures 6E, 6H, and 6M). Thus, larger clusters are inherently more likely to contact a cluster of ZAP-70 and trigger its phosphorylation.

The Size of KIR2DL1 Nanoclusters Is Important for SHP-1 Phosphorylation

To determine whether the size of KIR2DL1 nanoclusters also affects signaling, we imaged KIR2DL1 with SHP-1, the phosphatase recruited in response to inhibitory KIR ligation (Fry et al., 1996; Burshtyn et al., 1996), or SHP-1 phosphorylated on tyrosine 536 (SHP-1 pY536), as a mark of phosphatase activity (Zhang et al., 2003). Ligation of KIR2DL1, but not KIR2DL1$^{Y281A/Y311A}$, led to an increase in the fluorescence intensity of membrane-proximal SHP-1 and pSHP1 (Figure 7A, 7I, S7H, and S7I). Larger clusters of KIR2DL1 were more often associated with clusters of SHP-1, under both resting and activating conditions (Figures 7B, 7C, and 7F). pSHP-1 clusters were rarely detected on slides coated with control mAb, while clusters of phosphorylated SHP-1 detected upon KIR2DL1 ligation were preferentially in the vicinity of large KIR2DL1 clusters (Figures 7J and 7K). The median amount of SHP-1 and SHP-1 pY536 was also highest within the proximity of large KIR clusters (Figures 7E, 7H, and 7M). The frequency of contacts between differently sized KIR clusters and SHP-1 calculated for simulated datasets in which positions of SHP-1 clusters were randomized, was again comparable to the experimental data (Figures 7D, 7G, and 7L). Thus, larger clusters of KIR2DL1 are inherently more likely to contact SHP-1 clusters for signaling.

DISCUSSION

Over the past decade, it has emerged that immune cell receptors and ligands are organized into micrometer-scale domains at cell surfaces and immune synapses, and this organization is important for signaling in T cells, B cells, NK cells, and other immune cells (Davis and Dustin, 2004; Harwood and Batista, 2008; Orange, 2008). KIRs, like other signaling receptors, assemble into microclusters where phosphorylation occurs (Davis et al., 1999; Treanor et al., 2006; Abeyweera et al., 2011). Recently however, super-resolution microscopy has forced a revision to our understanding of how immune cell receptors organize within

(C) Mean DAP12-derived fluorescence intensity in NKL/KIR2DS1-HA cells on isotype control- and anti-KIR2DL/S1 mAb-coated slides. In (B) and (C), each symbol represents one cell. Horizontal bars and errors represent the medians and interquartile range.

(D–K) Quantification of interaction between differently sized KIR2DS1 clusters and DAP12 clusters in NKL/KIR2DS1-HA cells incubated on isotype control-coated (D, F–H) and anti-KIR2DL/S1 mAb-coated slides (E, I–K). In (D) and (E), 3 × 3 μm regions of cell membrane with KIR2DS1 (green) and DAP12 (red) are shown with overlapping pixels marked in white (left) or with KIR2DS1 clusters color-coded according to their size (right). Each size bin corresponds to one-third of all KIR2DS1 clusters. (F and I) The fractions of all KIR2DS1 clusters associated with DAP12 that fall into each size group. (G and J) The fractions of all KIR2DS1 clusters associated with DAP12 that fall into each size group in simulated data where the positions of KIR2DS1 clusters were maintained and positions of DAP12 clusters were randomized within the boundaries of cell area. (H and K) Average intensity of DAP12 clusters found nearby KIR2DS1 clusters from specified size bins. (F–K) Bars and errors represent the medians and interquartile range. Data are from 25–34 cells from two independent experiments. AU, arbitrary units; ns, non-significant. *$p < 0.05$, **$p < 0.01$, ***$p < 0.001$, ****$p < 0.0001$. Kruskal-Wallis test by ranks with Dunn's post-test (B), Mann-Whitney test (C), row-matched Friedman test with Dunn's post-test (F–K).

See also Figure S3 and Figure S7.

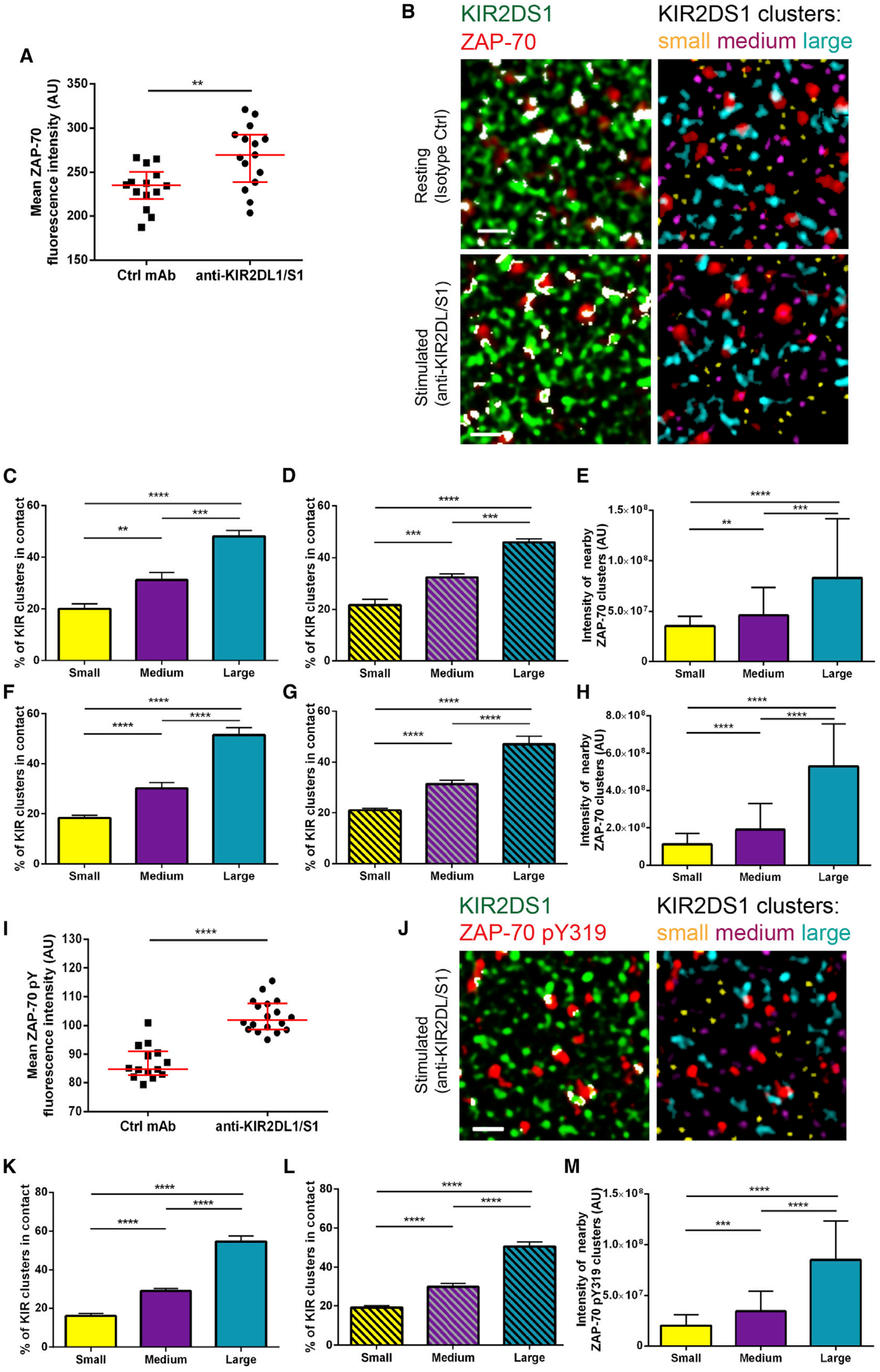

(legend on next page)

the cell membrane, by revealing sub-micron nanoscale receptor assemblies. However, little is understood about the mechanisms or functions of receptor nanoclusters.

Many factors including cholesterol-rich membrane domains (lipid rafts) and cortical actin have been implicated in controlling the clustering of surface proteins (Saka et al., 2014; Lagrue et al., 2013). Here, we demonstrate that the size of KIR nanoclusters is influenced by the transmembrane sequence. The positively charged residue within the transmembrane domain creates larger nanoclusters of KIR2DS1. The transmembrane lysine can affect the organization of KIR2DS1 directly or perhaps indirectly, for example by modulating an interaction in the endoplasmic reticulum, which modifies the protein's glycosylation (Mulrooney et al., 2013).

A crucial unknown is whether the size of immune receptor nanoclusters is functionally important. Primary antigen-experienced T cells have larger oligomers of TCR at their surface, which likely relate to TCR nanoclusters (Kumar et al., 2011). In NK cells, loss of histidine at position 36 in KIR2DL1 increases KIR self-association and ITIM phosphorylation (Kumar et al., 2015). B cell receptor, on the other hand, is signaling-incompetent within tight nanoclusters, but upon activation, clusters open up to permit signal amplification (Kläsener et al., 2014). However, from these studies, the importance of the size of nanoclusters is not obvious. To test whether the size of clusters affects signaling here, we directly imaged membrane-proximal signaling events by super-resolution microscopy. We found that SHP-1 is more efficiently recruited to and phosphorylated within larger KIR nanoclusters. Thus, inhibitory signaling would be preferentially transduced by larger nanoclusters of KIR2DL1. Similarly for activating signals, larger nanoclusters of KIR2DS1 were more often interacting with clusters of the signaling adaptor DAP12, and downstream kinase ZAP-70 was preferentially recruited to, and activated within, larger nanoclusters of KIR2DS1. This establishes a relationship between the size of NK cell receptor nanoclusters and signaling outcomes.

Comparison of the experimental data with simulated data in which the positions of signaling clusters were randomized, demonstrated that larger receptor clusters were inherently more likely to contact independently distributed clusters of signaling molecules. This implies that the size of protein nanoclusters can directly affect signaling efficiencies by influencing the likelihood that clusters meet by chance. Previous work from our lab established that cellular activation can affect the nanoscale organization of NK cell receptors (Pageon et al., 2013). Together with the current data, this implies that changes in nanoclustering could tune the thresholds at which immune cells are switched on and off, independently of changes in expression levels.

In addition to changing the sizes of clusters, cellular activation also changed the relative position of different nanoclusters. Here, we show that cellular activation led to increased coalescence of clusters of KIR2DS1 and its signaling adaptor DAP12. This may occur by recruitment of DAP12 clusters to the interface, since the level of expression of this adaptor increases upon ligation of KIR2DS1, as well as by a lateral reorganization of protein nanoclusters. Because KIR2DS1 only signals via this adaptor, the mixing of the two proteins would allow more KIR2DS1 molecules to become signaling-competent, thus providing a positive feedback-loop to amplify or sustain signaling. This model for NK cell activating receptors is reminiscent of a convergence of BCR and co-receptor CD19 postulated to be important for antigen stimulation of B cells (Mattila et al., 2013). Thus, coalescence of nanoclusters might be a common mechanism for spatiotemporal positive feedback for activating receptor signals. In summary, we have established that the nanometre-scale organization of NK cell receptors nanoclusters is controlled by the transmembrane sequence and affects signaling.

EXPERIMENTAL PROCEDURES

All donors were healthy and gave informed consent for their blood to be used in accordance with the Declaration of Helsinki (with ethics approved by The National Research Ethics Service, Ref 05/Q0401/108).

Cell Lines

The NK cell line NKL (a kind gift from Jack Strominger, Harvard University), leukemic T cell line Jurkat E6.1 (TIB-152, American Type Culture Collection), as well as B cell lines 721.221/Cw4 and 721.221/Cw4/MICA (used as target cells, as previously described; Pageon et al., 2013) were cultured in RPMI 1640 (Sigma) supplemented with 10% fetal bovine serum (FBS; Invitrogen), 2 mM L-glutamine, and 1% penicillin and streptomycin (both GIBCO). NKL were cultured in the presence of IL-2 (100 U/ml; Roche). All cell lines were routinely tested for mycoplasma infection using a PCR-based kit (PromoCell).

Figure 6. ZAP-70 Is More Often Recruited to and Phosphorylated at Larger KIR2DS1 Clusters

(A–M) NKL/KIR2DS1-HA cells were incubated for 5 min on isotype control-coated or anti-KIR2DL/S1 mAb-coated slides, fixed, stained with anti-HA mAb (AF488) and anti-ZAP-70 Ab (AF568; A–H) or anti-ZAP-70 pY319 Ab (AF568; I–M) and imaged with STED microscopy. (A and I) Mean ZAP-70 (A) or ZAP-70 pY319 (I) fluorescence intensity in NKL/KIR2DS1-HA cells on isotype control-coated and anti-KIR2DL/S1 mAb-coated slides. (B and J) 3 × 3 μm regions of cell membrane with KIR2DS1 (green) and ZAP-70 (B) or ZAP-70 pY319 (J; red) with overlapping pixels marked in white (left) or with KIR2DS1 clusters color-coded according to size (right). Each size bin corresponds to one-third of all KIR2DS1 clusters. (C and F) The fractions of all KIR2DS1 clusters associated with ZAP-70 that fall into each size group in cells on isotype control (C) or anti-KIR2DL/S1 (F) coated slides. (D and G) The fractions of all KIR2DS1 clusters associated with ZAP-70 that fall into each size group in simulated data where the positions of KIR2DS1 clusters were maintained and positions of ZAP-70 clusters were randomized within the boundaries of cell area, in cells on isotype control (D) or anti-KIR2DL/S1 (G) coated slides. (E and H) Average intensity of ZAP-70 clusters found nearby KIR2DS1 clusters from specified size bins in cells on isotype control (E) or anti-KIR2DL/S1 (H) mAb-coated slides. (K) The fraction of all KIR2DS1 clusters associated with ZAP-70 pY319 that fall into each size group, in cells on anti-KIR2DL/S1 mAb. (L) The fraction of all KIR2DS1 clusters associated with ZAP-70 pY319 that fall into each size group where ZAP-70 pY319 clusters were randomized within the boundaries of cell area. (M) Average intensity of ZAP-70 pY319 clusters found nearby KIR2DS1 clusters from each size bin. (A and I) Each symbol represents one cell. Horizontal bars and errors represent the medians and interquartile range. (C–H and K–M) Bars and errors represent the medians and interquartile range. The data are from 13–18 cells from two independent experiments (A and I) or 29–39 cells from three independent experiments (C–H and K–M). AU, arbitrary units. **$p < 0.01$,***$p < 0.001$, ****$p < 0.0001$, Mann- Whitney test (A) and (I) or row-matched Friedman test with Dunn's post-test (C–H and K–M).

See also Figure S7.

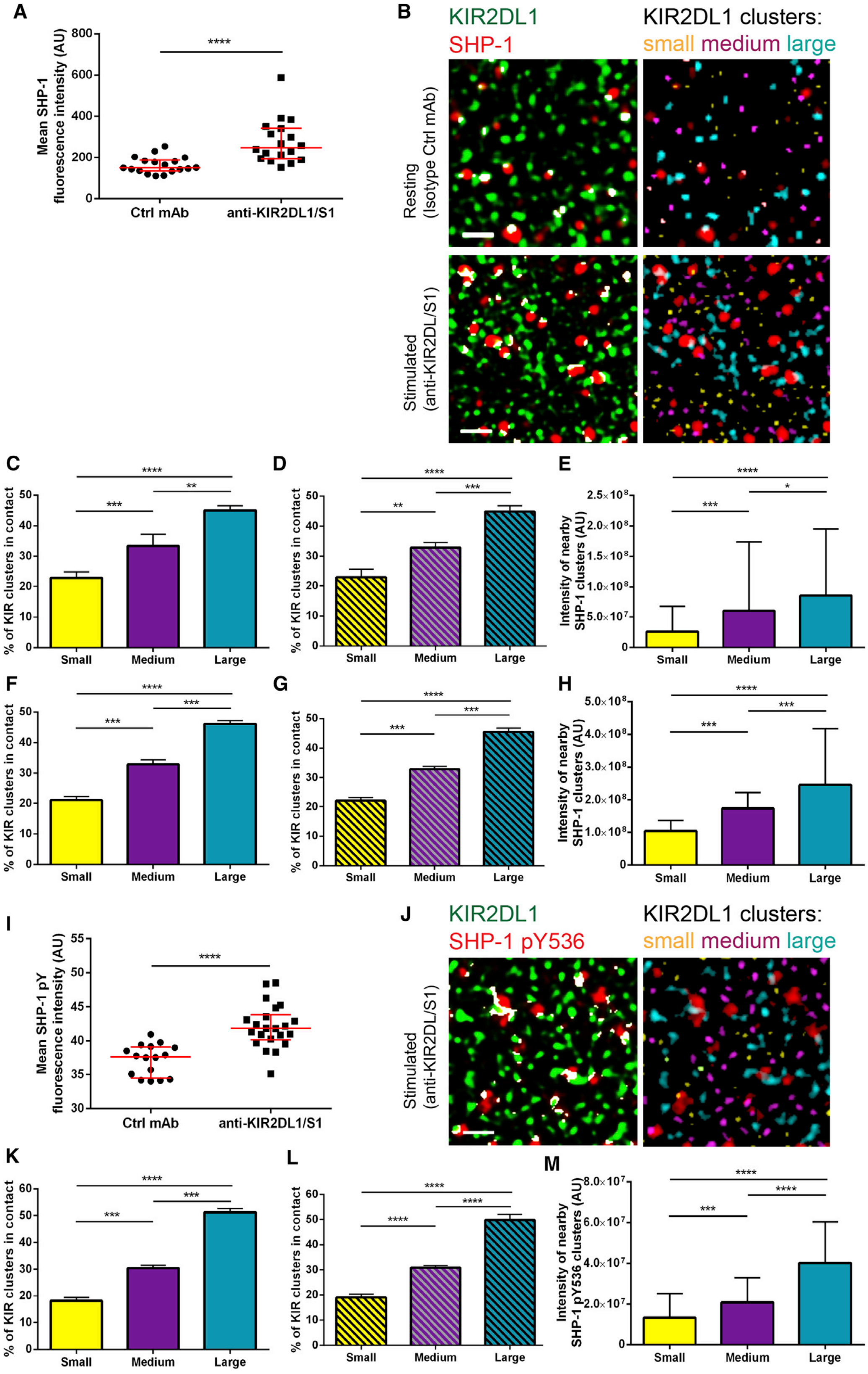

(legend on next page)

All transfectants were generated by retroviral transduction (as detailed in the Supplemental Experimental Procedures). Expression of the receptors was checked by flow cytometry, and their functionality was confirmed by assessing spreading response and IFN-γ production, as detailed in the Supplemental Experimental Procedures.

Generation of NK Clones

NK clones were generated from NK cells freshly isolated from peripheral blood of healthy donors by negative magnetic selection (NK cell isolation kit, Miltenyi Biotec) by plating single NK cells in individual wells of 96-well plates (details can be found in the Supplemental Experimental Procedures). Cells were tested by flow cytometry for clonality and the presence of KIR2DS1 and KIR2DL1. Additionally, cells were checked for expression of KIR2DL3 due to cross-reactivity of the anti-KIR2DL/S1 mAb (EB6) with the KIR2DL3*005 allele (Falco et al., 2010), using DX27 mAb (Biolegend, three clones), or 180701 mAb (R&D Systems, two clones), and 2DL3-positive cells were excluded.

Sample Preparation for Imaging

Cells were plated on chambered glass coverslips pre-coated with PLL, fixed and blocked as detailed in the Supplemental Experimental Procedures. For stimulation of cells, slides were additionally coated with 5 μg/ml anti-KIR2DL/S1 mAb (clone EB6) or murine IgG1. KIR2DL1, KIR2DS1, IL-2R alpha subunit, DAP12, ZAP-70, ZAP-70 pY319, SHP-1, and SHP-1 pY536 were fluorescently labeled with antibody, as detailed in the Supplemental Experimental Procedures. To check for changes in membrane organization induced by contact with a glass surface we also compared KIR2DL1 and KIR2DS1 clustering in NK cells fixed in suspension, as detailed in the Supplemental Experimental Procedures.

Microscopy

Single-molecule localization microscopy images were acquired on a Leica SR GSD microscope and STED images were acquired on Leica TCS SP8 STED CW microscope, as detailed in the Supplemental Experimental Procedures.

Image Analysis

GSD image reconstruction was performed using Thunderstorm software (Ovesný et al., 2014), as detailed in the Supplemental Experimental Procedures. Multiple regions of 3 × 3 μm were selected for quantitative analysis based on Ripley's *K* function (Ripley, 1977) and univariate Getis and Franklin's local point pattern analysis (Getis and Franklin, 1987) method, as described previously (Williamson et al., 2011; Pageon et al., 2013).

For comparison of the intensity of fluorescence derived from signaling molecules in STED images, the mean fluorescence intensity per pixel was measured in raw STED images in ImageJ. The size of pixel in the compared images was kept constant. To compare the area of clusters, the co-localization of proteins and the distances between cluster centroids as well as to identify clusters localized within a close proximity, STED images were deconvolved in Huygens Professional 10.1 software (Scientific Volume Imaging). Images were converted into binary maps for quantitative comparison of clusters and measurements of the distances between cluster centroids. To assess protein co-localization, Pearson correlation coefficients were calculated using an ImageJ (Schneider et al., 2012) plug-in Coloc 2 (written by Daniel J. White, Tom Kazimiers, and Johannes Schindelin; available online), which uses the Costes auto-threshold.

To identify clusters of signaling molecules overlapping or in contact with KIR clusters or in proximity, a circular area around the centroid of each KIR2DL/S1 cluster was searched for the presence of DAP12/SHP-1/ZAP-70 clusters. The results were compared to simulated datasets in which positions of signaling molecules clusters were randomized within the cell area. Fractions of KIR2DL/S1 clusters with at least one signaling molecule cluster nearby and relative efficiency of phosphorylation within clusters of certain sizes were quantified in a custom- MATLAB script.

Contrast and brightness of the representative images in the figures has been adjusted to make details visible but all quantitative analysis has been performed on unprocessed files and datasets throughout the manuscript.

Detailed information on image analysis can be found in the Supplemental Experimental Procedures.

Statistical Analysis

Sample sizes chosen were appropriate to provide enough power for statistical tests used in this study (StatMate software, GraphPad Software). The statistical significance of differences between two datasets was assessed by Mann-Whitney test comparing ranks; multiple comparisons were made with Kruskal-Wallis test by ranks or matched-values Friedman test with Dunn's post-testing. All statistical analyses were performed using GraphPrism 6.0 (GraphPad Software).

SUPPLEMENTAL INFORMATION

Supplemental Information includes Supplemental Experimental Procedures, seven figures, and one table and can be found with this article online at http://dx.doi.org/10.1016/j.celrep.2016.04.075.

AUTHOR CONTRIBUTIONS

A.O., S-P.C., and D.M.D. conceived the project; A.O., S-P.C., and K.S. generated cell lines and NK clones; A.O. and D.M.D designed experiments and wrote the manuscript; A.O performed most of the experiments; D.J.M. and P.R.K performed additional experiments; D.J.W wrote MATLAB scripts; and A.O. and D.J.W. analyzed the data.

ACKNOWLEDGMENTS

We thank Alex Carisey and other members of our laboratory for useful discussions, Pawel Paszek for advice on statistical analysis, and Gareth Howell for

Figure 7. SHP-1 Is More Often Recruited to and Phosphorylated at Larger KIR2DL1 Clusters

(A–M) NKL/KIR2DL1-HA cells were incubated for 5 min on isotype control-coated or anti-KIR2DL/S1 mAb-coated slides, fixed, stained with anti-HA mAb (AF488) and anti-SHP-1 mAb (AF568; A–H) or anti-SHP-1 pY536 Ab (AF568; I–M) and imaged with STED microscopy. (A and I) Mean SHP-1 (A) or SHP-1 pY536 (I) fluorescence intensity in NKL/KIR2DL1-HA cells on isotype control-coated and anti-KIR2DL/S1 mAb-coated slides. (B and J) 3 × 3 μm regions of cell membrane with KIR2DL1 (green) and SHP-1 (B; red) or SHP-1 pY536 (J; red) with overlapping pixels marked in white (left) or with KIR2DL1 clusters color-coded according to their size (right). Each size bin corresponds to one-third of all KIR2DL1 clusters. (C and F) The fractions of all KIR2DL1 clusters associated with SHP-1 that fall into each size group, in cells on isotype control (C) or anti-KIR2DL/S1 (F) mAb. (D and G) The fractions of all KIR2DL1 clusters associated with SHP-1 that fall into each size group in simulated datasets where the positions of KIR2DL1 clusters were maintained and positions of SHP-1 clusters were randomized within the boundaries of cell area, in cells on isotype control (D) or anti-KIR2DL/S1 (G) mAb. (E and H) Average intensity of SHP-1 clusters found nearby KIR2DL1 clusters from specified size bins in cells on isotype control (E) or anti-KIR2DL/S1 (H) mAb. (K) The fraction of all KIR2DL1 clusters associated with SHP-1 pY536 that fall into each size group in cells on anti-KIR2DL/S1 mAb. (L) The fraction of all KIR2DL1 clusters associated with SHP-1 pY536 that fall into each size group in simulated datasets where the positions of KIR2DL1 clusters were maintained and positions of SHP-1 pY536 clusters were randomized within the boundaries of cell area. (M) Average intensity of SHP-1 pY536 clusters found nearby KIR2DL1 clusters from each size bin. (A and I) Each symbol represents one cell. Horizontal bars and errors represent the medians and interquartile range. (C–H and K–M) Bars and errors represent the medians and interquartile range. The data are from 14–22 cells from two independent experiments (A and I) or 27–33 cells from three independent experiments (C–H and K–M). AU, arbitrary units. *$p < 0.05$, **$p < 0.01$,***$p < 0.001$, ****$p < 0.0001$, Mann- Whitney test (A and I) or row-matched Friedman test with Dunn's post-test (C–H and K–M).
See also Figure S7.

help with flow cytometry. This work was supported by the Medical Research Council (award G1001044), a Wellcome Trust Investigator Award (110091), and the Manchester Collaborative Centre for Inflammation Research (funded by a pre-competitive open innovation award from GSK, AstraZeneca, and The University of Manchester, UK).

Received: November 6, 2015
Revised: March 15, 2016
Accepted: April 20, 2016
Published: May 19, 2016

REFERENCES

Abeyweera, T.P., Merino, E., and Huse, M. (2011). Inhibitory signaling blocks activating receptor clustering and induces cytoskeletal retraction in natural killer cells. J. Cell Biol. *192*, 675–690.

Alter, G., Martin, M.P., Teigen, N., Carr, W.H., Suscovich, T.J., Schneidewind, A., Streeck, H., Waring, M., Meier, A., Brander, C., et al. (2007). Differential natural killer cell-mediated inhibition of HIV-1 replication based on distinct KIR/HLA subtypes. J. Exp. Med. *204*, 3027–3036.

Alter, G., Heckerman, D., Schneidewind, A., Fadda, L., Kadie, C.M., Carlson, J.M., Oniangue-Ndza, C., Martin, M., Li, B., Khakoo, S.I., et al. (2011). HIV-1 adaptation to NK-cell-mediated immune pressure. Nature *476*, 96–100.

Biassoni, R., Pessino, A., Malaspina, A., Cantoni, C., Bottino, C., Sivori, S., Moretta, L., and Moretta, A. (1997). Role of amino acid position 70 in the binding affinity of p50.1 and p58.1 receptors for HLA-Cw4 molecules. Eur. J. Immunol. *27*, 3095–3099.

Bottino, C., Falco, M., Sivori, S., Moretta, L., Moretta, A., and Biassoni, R. (2000). Identification and molecular characterization of a natural mutant of the p50.2/KIR2DS2 activating NK receptor that fails to mediate NK cell triggering. Eur. J. Immunol. *30*, 3569–3574.

Burshtyn, D.N., Scharenberg, A.M., Wagtmann, N., Rajagopalan, S., Berrada, K., Yi, T., Kinet, J.P., and Long, E.O. (1996). Recruitment of tyrosine phosphatase HCP by the killer cell inhibitor receptor. Immunity *4*, 77–85.

Carr, W.H., Rosen, D.B., Arase, H., Nixon, D.F., Michaelsson, J., and Lanier, L.L. (2007). Cutting Edge: KIR3DS1, a gene implicated in resistance to progression to AIDS, encodes a DAP12-associated receptor expressed on NK cells that triggers NK cell activation. J. Immunol. *178*, 647–651.

Davis, D.M. (2014). The Compatibility Gene (Penguin Books).

Davis, D.M., and Dustin, M.L. (2004). What is the importance of the immunological synapse? Trends Immunol. *25*, 323–327.

Davis, D.M., Chiu, I., Fassett, M., Cohen, G.B., Mandelboim, O., and Strominger, J.L. (1999). The human natural killer cell immune synapse. Proc. Natl. Acad. Sci. USA *96*, 15062–15067.

Della Chiesa, M., Marcenaro, E., Sivori, S., Carlomagno, S., Pesce, S., and Moretta, A. (2014). Human NK cell response to pathogens. Semin. Immunol. *26*, 152–160.

Falco, M., Romeo, E., Marcenaro, S., Martini, S., Vitale, M., Bottino, C., Mingari, M.C., Moretta, L., Moretta, A., and Pende, D. (2010). Combined genotypic and phenotypic killer cell Ig-like receptor analyses reveal KIR2DL3 alleles displaying unexpected monoclonal antibody reactivity: identification of the amino acid residues critical for staining. J. Immunol. *185*, 433–441.

Fauriat, C., Ivarsson, M.A., Ljunggren, H.G., Malmberg, K.J., and Michaëlsson, J. (2010). Education of human natural killer cells by activating killer cell immunoglobulin-like receptors. Blood *115*, 1166–1174.

Feng, J., Call, M.E., and Wucherpfennig, K.W. (2006). The assembly of diverse immune receptors is focused on a polar membrane-embedded interaction site. PLoS Biol. *4*, e142.

Foley, B., Felices, M., Cichocki, F., Cooley, S., Verneris, M.R., and Miller, J.S. (2014). The biology of NK cells and their receptors affects clinical outcomes after hematopoietic cell transplantation (HCT). Immunol. Rev. *258*, 45–63.

Fry, A.M., Lanier, L.L., and Weiss, A. (1996). Phosphotyrosines in the killer cell inhibitory receptor motif of NKB1 are required for negative signaling and for association with protein tyrosine phosphatase 1C. J. Exp. Med. *184*, 295–300.

Getis, A., and Franklin, J. (1987). Second-Order Neighborhood Analysis of Mapped Point Patterns. Ecology *68*, 473–477.

Guia, S., Jaeger, B.N., Piatek, S., Mailfert, S., Trombik, T., Fenis, A., Chevrier, N., Walzer, T., Kerdiles, Y.M., Marguet, D., et al. (2011). Confinement of activating receptors at the plasma membrane controls natural killer cell tolerance. Sci. Signal. *4*, ra21.

Harwood, N.E., and Batista, F.D. (2008). New insights into the early molecular events underlying B cell activation. Immunity *28*, 609–619.

Hiby, S.E., Apps, R., Sharkey, A.M., Farrell, L.E., Gardner, L., Mulder, A., Claas, F.H., Walker, J.J., Redman, C.W., Morgan, L., et al. (2010). Maternal activating KIRs protect against human reproductive failure mediated by fetal HLA-C2. J. Clin. Invest. *120*, 4102–4110.

Ivarsson, M.A., Michaëlsson, J., and Fauriat, C. (2014). Activating killer cell Ig-like receptors in health and disease. Front. Immunol. *5*, 184.

Khakoo, S.I., Thio, C.L., Martin, M.P., Brooks, C.R., Gao, X., Astemborski, J., Cheng, J., Goedert, J.J., Vlahov, D., Hilgartner, M., et al. (2004). HLA and NK cell inhibitory receptor genes in resolving hepatitis C virus infection. Science *305*, 872–874.

Kläsener, K., Maity, P.C., Hobeika, E., Yang, J., and Reth, M. (2014). B cell activation involves nanoscale receptor reorganizations and inside-out signaling by Syk. eLife *3*, e02069.

Köhler, K., Xiong, S., Brzostek, J., Mehrabi, M., Eissmann, P., Harrison, A., Cordoba, S.P., Oddos, S., Miloserdov, V., Gould, K., et al. (2010). Matched sizes of activating and inhibitory receptor/ligand pairs are required for optimal signal integration by human natural killer cells. PLoS ONE *5*, e15374.

Kumar, R., Ferez, M., Swamy, M., Arechaga, I., Rejas, M.T., Valpuesta, J.M., Schamel, W.W., Alarcon, B., and van Santen, H.M. (2011). Increased sensitivity of antigen-experienced T cells through the enrichment of oligomeric T cell receptor complexes. Immunity *35*, 375–387.

Kumar, S., Sarkar, P., Sim, M.J., Rajagopalan, S., Vogel, S.S., and Long, E.O. (2015). A single amino acid change in inhibitory killer cell Ig-like receptor results in constitutive receptor self-association and phosphorylation. J. Immunol. *194*, 817–826.

Lagrue, K., Carisey, A., Oszmiana, A., Kennedy, P.R., Williamson, D.J., Cartwright, A., Barthen, C., and Davis, D.M. (2013). The central role of the cytoskeleton in mechanisms and functions of the NK cell immune synapse. Immunol. Rev. *256*, 203–221.

Lanier, L.L., Corliss, B.C., Wu, J., Leong, C., and Phillips, J.H. (1998). Immunoreceptor DAP12 bearing a tyrosine-based activation motif is involved in activating NK cells. Nature *391*, 703–707.

Mandelboim, O., Kent, S., Davis, D.M., Wilson, S.B., Okazaki, T., Jackson, R., Hafler, D., and Strominger, J.L. (1998). Natural killer activating receptors trigger interferon gamma secretion from T cells and natural killer cells. Proc. Natl. Acad. Sci. USA *95*, 3798–3803.

Mattila, P.K., Feest, C., Depoil, D., Treanor, B., Montaner, B., Otipoby, K.L., Carter, R., Justement, L.B., Bruckbauer, A., and Batista, F.D. (2013). The actin and tetraspanin networks organize receptor nanoclusters to regulate B cell receptor-mediated signaling. Immunity *38*, 461–474.

Moretta, A., Sivori, S., Vitale, M., Pende, D., Morelli, L., Augugliaro, R., Bottino, C., and Moretta, L. (1995). Existence of both inhibitory (p58) and activatory (p50) receptors for HLA-C molecules in human natural killer cells. J. Exp. Med. *182*, 875–884.

Mulrooney, T.J., Posch, P.E., and Hurley, C.K. (2013). DAP12 impacts trafficking and surface stability of killer immunoglobulin-like receptors on natural killer cells. J. Leukoc. Biol. *94*, 301–313.

Orange, J.S. (2008). Formation and function of the lytic NK-cell immunological synapse. Nat. Rev. Immunol. *8*, 713–725.

Ovesný, M., Křížek, P., Borkovec, J., Svindrych, Z., and Hagen, G.M. (2014). ThunderSTORM: a comprehensive ImageJ plug-in for PALM and STORM data analysis and super-resolution imaging. Bioinformatics *30*, 2389–2390.

Pageon, S.V., Rudnicka, D., and Davis, D.M. (2012). Illuminating the dynamics of signal integration in Natural Killer cells. Front. Immunol. *3*, 308.

Pageon, S.V., Cordoba, S.P., Owen, D.M., Rothery, S.M., Oszmiana, A., and Davis, D.M. (2013). Superresolution microscopy reveals nanometer-scale reorganization of inhibitory natural killer cell receptors upon activation of NKG2D. Sci. Signal. *6*, ra62.

Ripley, B.D. (1977). Modeling spatial patterns. J. R. Stat. Soc. B *39*, 172–212.

Rose, M.J., Brooks, A.G., Stewart, L.A., Nguyen, T.H., and Schwarer, A.P. (2009). Killer Ig-like receptor ligand mismatch directs NK cell expansion in vitro. J. Immunol. *183*, 4502–4508.

Saka, S.K., Honigmann, A., Eggeling, C., Hell, S.W., Lang, T., and Rizzoli, S.O. (2014). Multi-protein assemblies underlie the mesoscale organization of the plasma membrane. Nat. Commun. *5*, 4509.

Schneider, C.A., Rasband, W.S., and Eliceiri, K.W. (2012). NIH Image to ImageJ: 25 years of image analysis. Nat. Methods *9*, 671–675.

Sivori, S., Carlomagno, S., Falco, M., Romeo, E., Moretta, L., and Moretta, A. (2011). Natural killer cells expressing the KIR2DS1-activating receptor efficiently kill T-cell blasts and dendritic cells: implications in haploidentical HSCT. Blood *117*, 4284–4292.

Stebbins, C.C., Watzl, C., Billadeau, D.D., Leibson, P.J., Burshtyn, D.N., and Long, E.O. (2003). Vav1 dephosphorylation by the tyrosine phosphatase SHP-1 as a mechanism for inhibition of cellular cytotoxicity. Mol. Cell. Biol. *23*, 6291–6299.

Stewart, C.A., Laugier-Anfossi, F., Vély, F., Saulquin, X., Riedmuller, J., Tisserant, A., Gauthier, L., Romagné, F., Ferracci, G., Arosa, F.A., et al. (2005). Recognition of peptide-MHC class I complexes by activating killer immunoglobulin-like receptors. Proc. Natl. Acad. Sci. USA *102*, 13224–13229.

Treanor, B., Lanigan, P.M., Kumar, S., Dunsby, C., Munro, I., Auksorius, E., Culley, F.J., Purbhoo, M.A., Phillips, D., Neil, M.A., et al. (2006). Microclusters of inhibitory killer immunoglobulin-like receptor signaling at natural killer cell immunological synapses. J. Cell Biol. *174*, 153–161.

Valés-Gómez, M., Reyburn, H.T., Erskine, R.A., and Strominger, J. (1998). Differential binding to HLA-C of p50-activating and p58-inhibitory natural killer cell receptors. Proc. Natl. Acad. Sci. USA *95*, 14326–14331.

Vivier, E., Ugolini, S., Blaise, D., Chabannon, C., and Brossay, L. (2012). Targeting natural killer cells and natural killer T cells in cancer. Nat. Rev. Immunol. *12*, 239–252.

Watzl, C., Stebbins, C.C., and Long, E.O. (2000). NK cell inhibitory receptors prevent tyrosine phosphorylation of the activation receptor 2B4 (CD244). J. Immunol. *165*, 3545–3548.

Williamson, D.J., Owen, D.M., Rossy, J., Magenau, A., Wehrmann, M., Gooding, J.J., and Gaus, K. (2011). Pre-existing clusters of the adaptor Lat do not participate in early T cell signaling events. Nat. Immunol. *12*, 655–662.

Xiong, S., Sharkey, A.M., Kennedy, P.R., Gardner, L., Farrell, L.E., Chazara, O., Bauer, J., Hiby, S.E., Colucci, F., and Moffett, A. (2013). Maternal uterine NK cell-activating receptor KIR2DS1 enhances placentation. J. Clin. Invest. *123*, 4264–4272.

Zhang, Z., Shen, K., Lu, W., and Cole, P.A. (2003). The role of C-terminal tyrosine phosphorylation in the regulation of SHP-1 explored via expressed protein ligation. J. Biol. Chem. *278*, 4668–4674.

Cell Reports, Volume *15*

Supplemental Information

The Size of Activating and Inhibitory Killer Ig-like Receptor Nanoclusters Is Controlled by the Transmembrane Sequence and Affects Signaling

Anna Oszmiana, David J. Williamson, Shaun-Paul Cordoba, David J. Morgan, Philippa R. Kennedy, Kevin Stacey, and Daniel M. Davis

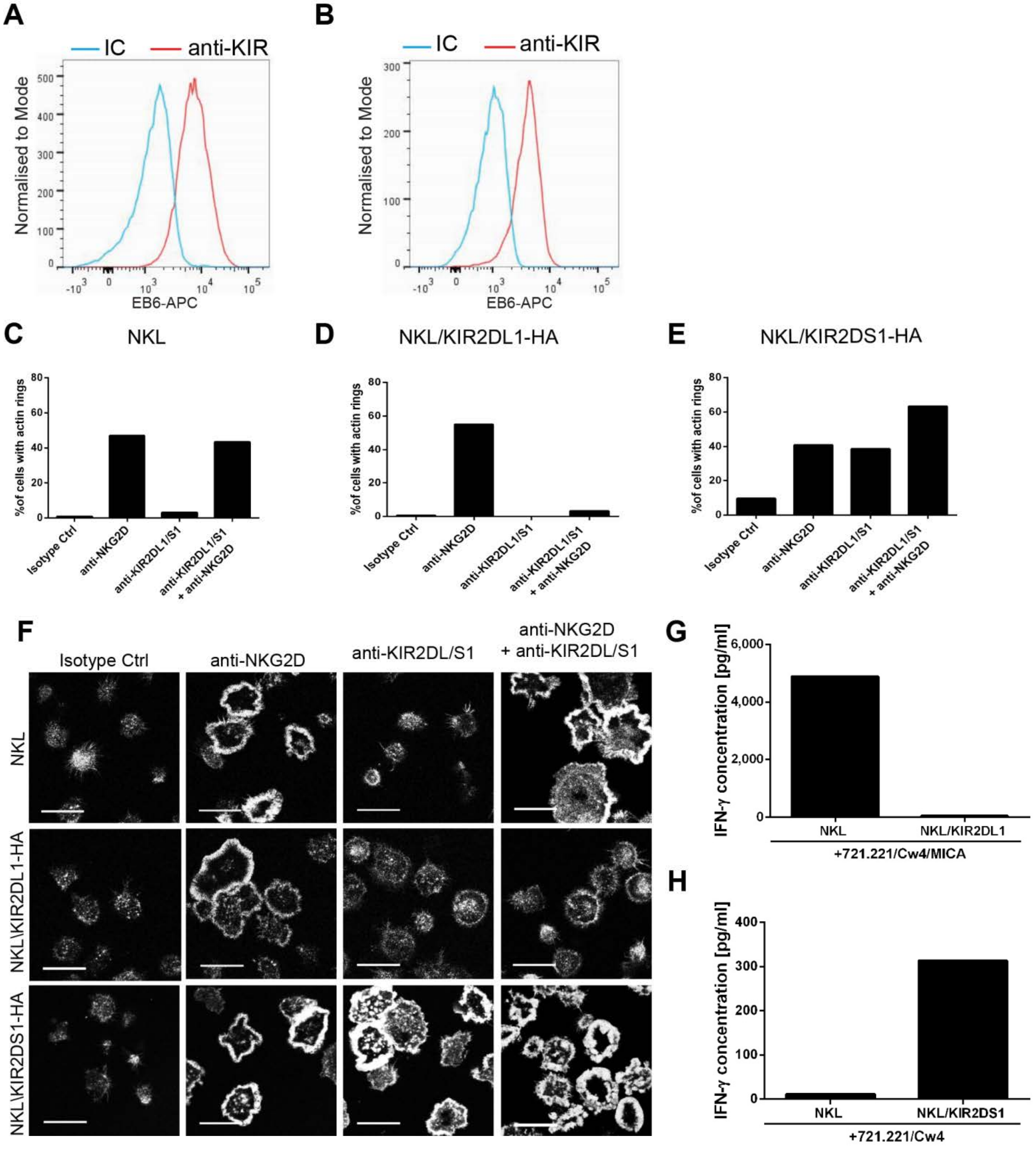

Figure S1. Generation and characterization of NKL/KIR2DL1-HA and NKL/KIR2DS1-HA transfectants (referring to figure 1). NKL/KIR2DL1-HA and NKL/KIR2DS1-HA transfectants were created by retroviral transduction of the human NK cell line NKL. NKL/KIR2DL1-HA (**A**) and NKL/KIR2DS1-HA (**B**) cells (red) were stained with anti-KIR2DL/S1 mAb (clone EB6) conjugated with APC and analyzed by flow cytometry. Respective cells stained with isotype-matched control mAb (blue) were analyzed as negative controls. (**C**-**F**) Parental NKL cells (**C**), NKL/KIR2DL1-HA (**D**) and NKL/KIR2DS1-HA (**E**) transfectants were incubated for 10 min at 37°C on slides coated with anti-KIR2DL/S1 or anti-NKG2D mAb at 5 µg/ml plus 5 µg/ml of murine IgG1, 10 µg/ml of IgG1 or 5 µg/ml of anti-KIR2DL/S1 plus 5 µg/ml of anti-NKG2D mAb. Filamentous actin was visualized using fluorescently labeled phalloidin and percentages of cells forming peripheral actin rings were quantified. One representative experiment of two is shown, n ≥ 100 cells per condition. Representative fields of view are shown in (**F**). Scale bars 5 µm. (**G**-**H**) Parental NKL cells and NKL/KIR2DL1-HA (**G**) or NKL/KIR2DS1-HA (**H**) were co-cultured for 24 hours with 721.221 transfectants expressing HLA-Cw4 and MICA (**G**) or -Cw4 only (**H**). IFN-γ release was measured by ELISA. E:T ratio was 10:1 in all experiments. One representative experiment of two is shown.

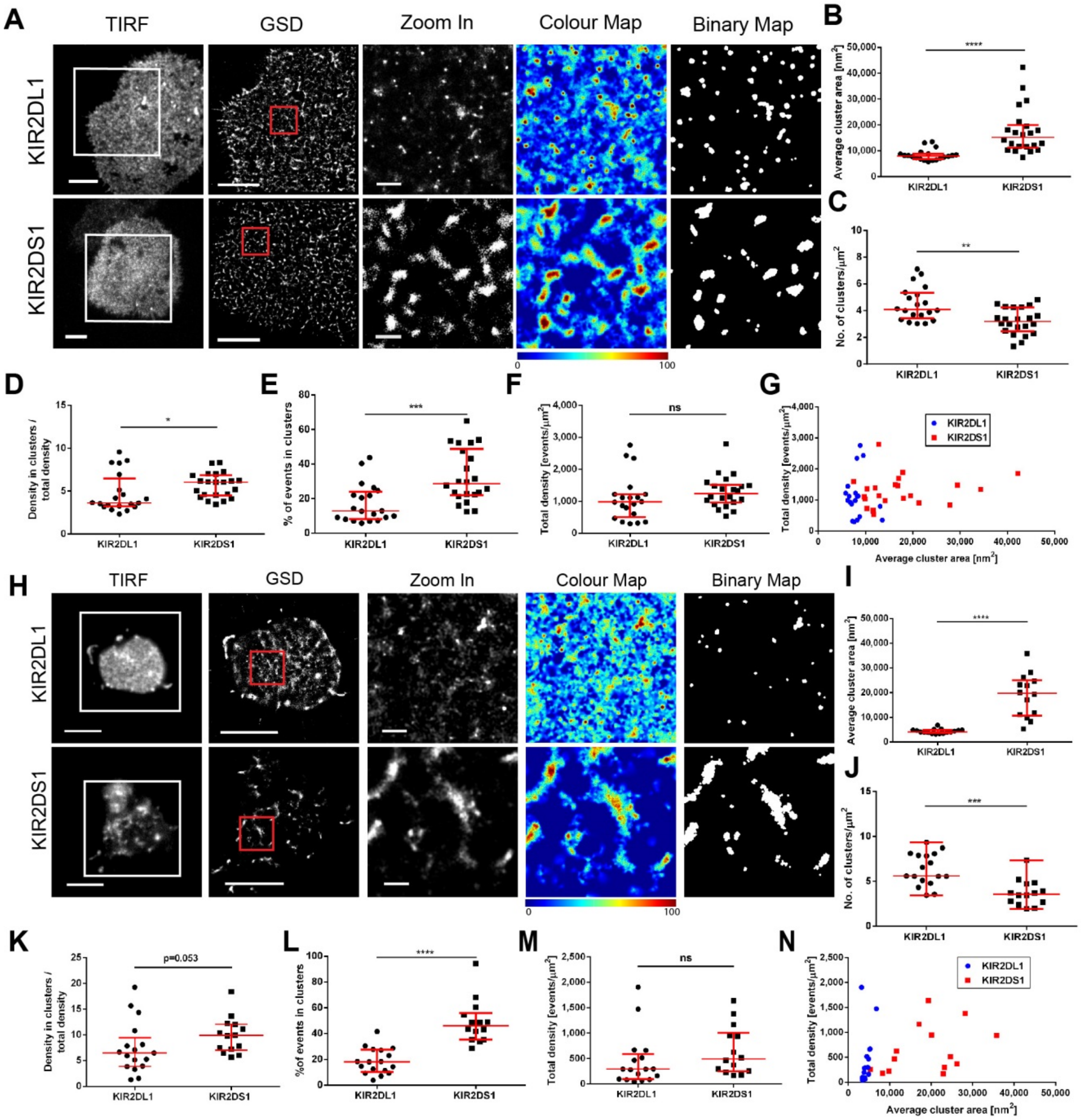

Figure S2. KIR2DL1-HA and KIR2DS1-HA display different clustering patterns at the surface of human NK cells irrespective of labeling strategy and contact with glass slides (referring to figure 1). (**A-G**) NKL/KIR2DL1-HA and NKL/KIR2DS1-HA cells were incubated on slides coated with PLL and stained with anti-HA mAb labeled with AF488. (**A**) Representative TIRF and GSD images of NKL/KIR2DL1-HA and NKL/KIR2DS1-HA cells stained with anti-HA mAb. The 3 × 3 μm regions (red boxes in GSD images) are zoomed-in and shown with corresponding color maps and binary maps. Colors correspond to the L(30) values. (**B** to **F**) Quantitative analysis of KIR2DL1 and KIR2DS1 clustering in NKL/KIR2DL1-HA and NKL/KIR2DS1-HA cells stained with anti-HA mAb. (**G**) Total density of detected molecules plotted against average cluster area measured for individual NKL/KIR2DL1-HA and NKL/KIR2DS1-HA cells stained with anti-HA mAb. Data are from 20 (KIR2DL1) and 22 (KIR2DS1) cells from three independent experiments. (**H** to **N**) The same cells were fixed in suspension, stained with anti-KIR2DL/S1 mAb labeled with AF647, thoroughly washed and added to chambered glass slides pre-coated with PLL. (**H**) Representative TIRF and GSD images of NKL/KIR2DL1-HA and NKL/KIR2DS1-HA cells fixed in suspension. The 3 × 3 μm regions are zoomed-in and shown with corresponding color maps and binary maps. (**I** to **M**) Quantitative analysis of KIR2DL1 and KIR2DS1 clustering in NKL/KIR2DL1-HA and NKL/KIR2DS1-HA cells fixed in suspension. (**N**) Total density of detected molecules plotted against average cluster area measured for individual NKL/KIR2DL1-HA and NKL/KIR2DS1-HA cells fixed in suspension. Data are from 17 (KIR2DL1) and 14 (KIR2DS1) cells from two independent experiments. Each symbol represents the mean from several regions within one cell. Horizontal bars and errors represent the medians and interquartile range. ns non-significant, * $p < 0.05$, ** $p < 0.01$, *** $p < 0.001$, **** $p < 0.0001$, Mann-Whitney test. Scale bars 5 μm in TIRF images and 500 nm in zoomed-in regions.

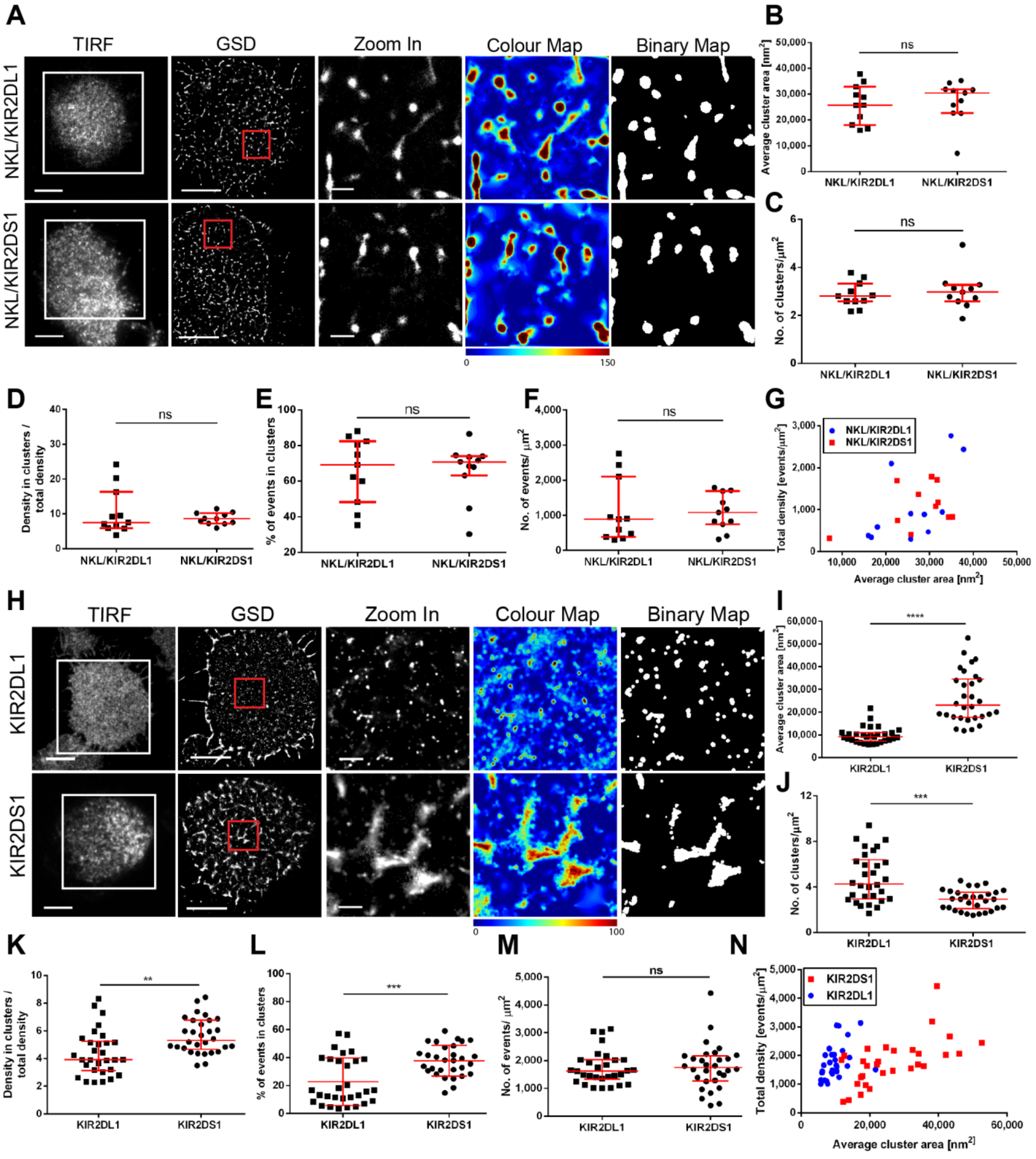

Figure S3. Differential organization of KIR2DL1 and KIR2DS1 is not a result of general differences in plasma membrane organization and does not depend on the association with DAP12 (referring to figure 1 and 5). (**A**) Representative TIRF and GSD images of IL-2Rα labeled with AF 488 in NKL/KIR2DL1-HA and NKL/KIR2DS1-HA cells on slides coated with PLL. The 3 × 3 µm regions (red boxes in GSD images) are zoomed-in and shown with corresponding color maps and binary maps. (**B** to **G**) Quantitative analysis of IL-2Rα clustering in NKL/KIR2DL1-HA and NKL/KIR2DS1-HA cells. (**G**) Total density of detected events plotted against average IL-2Rα cluster area in individual NKL/KIR2DL1-HA and NKL/KIR2DS1-HA cells. (**H**) Representative TIRF and GSD images of Jurkat/KIR2DL1-HA and Jurkat/KIR2DS1-HA cells stained with anti-HA mAb labeled with AF488. The 3 × 3 µm regions (red boxes) are zoomed-in and shown with corresponding color and binary maps. (**I** to **M**) Quantitative analysis of KIR2DL1 and KIR2DS1 clustering in Jurkat cells. (**N**) Total density of detected events plotted against average KIR2DL1 and KIR2DS1 cluster area in individual Jurkat cells. (**B - G** and **I - N**) Each symbol represents the mean from several regions within one cell. Horizontal bars and errors represent the medians and interquartile range. Data are from 11 cells from two independent experiments (**B** – **G**) or 30 cells from 3 independent experiments (**I** – **N**). Scale bars 5 µm in TIRF and 500 nm in zoomed-in regions. ns non-significant, ** $p < 0.01$, *** $p < 0.001$, Mann-Whitney test.

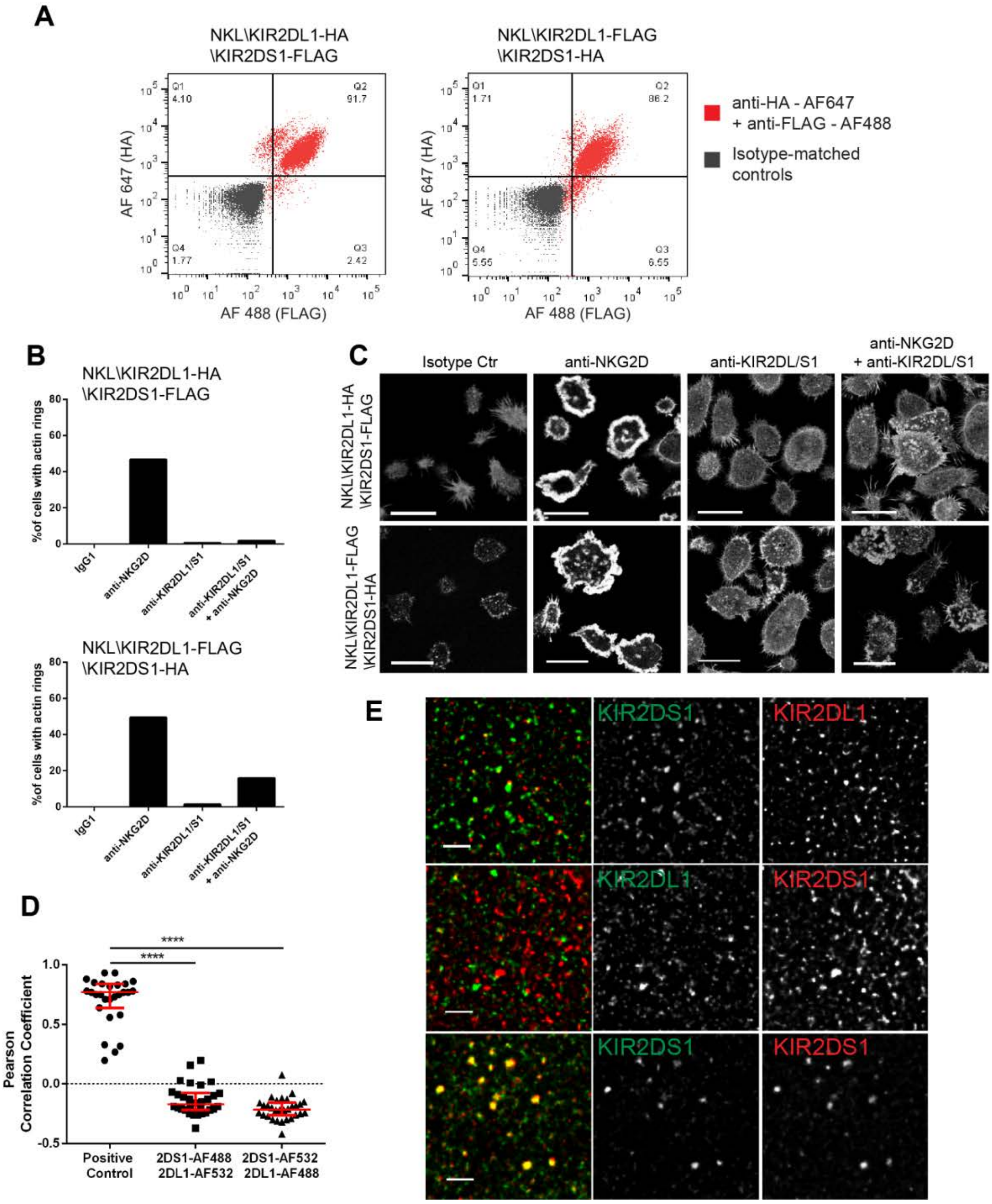

Figure S4. KIR2DL1 and KIR2DS1 are not localized in the same clusters in double-positive cells (referring to figure 1). NKL/KIR2DL1-HA/KIR2DS1-FLAG and NKL/KIR2DL1-FLAG/KIR2DS1-HA cell lines were created by retroviral transduction of human NK cell line NKL. (**A**) NKL/KIR2DL1-HA/KIR2DS1-FLAG and NKL/KIR2DL1-FLAG/KIR2DS1-HA cells were fixed, permeabilized, stained with anti-HA mAb conjugated with AF647 and anti-FLAG mAb conjugated with AF488 (red) and analyzed by flow cytometry. Respective cells stained with isotype-matched control mAb (grey) were analyzed as negative controls. (**B**-**C**) NKL/KIR2DL1-HA/KIR2DS1-FLAG and NKL/KIR2DL1-FLAG/KIR2DS1-HA transfectants were incubated for 10 min at 37°C on slides coated with anti-KIR2DL/S1 or anti-NKG2D mAb at 5 µg/ml plus 5 µg/ml of murine IgG1, 10 µg/ml of IgG1 or 5 µg/ml of anti-KIR2DL/S1 plus 5 µg/ml of anti-NKG2D mAb. Actin was visualized using fluorescently labeled phalloidin and percentages of cells forming peripheral actin rings were calculated. One representative experiment of three is shown, n ≥ 100 cells per condition. Representative fields of view are shown in (**C**). (**D**-**E**) NKL/KIR2DL1-FLAG/KIR2DS1-HA and NKL/KIR2DL1-HA/KIR2DS1-FLAG cells on PLL-coated slides were stained with anti-HA mAb (AF488) and anti-FLAG mAb (AF532) and imaged by STED microscopy. As a positive control, NKL/KIR2DS1-HA cells were stained with anti-HA mAb (AF488) and anti-KIR2DL/S1 mAb (AF532). (**D**) Pearson's correlation coefficient values calculated for KIR2DL1 and KIR2DS1 are compared to KIR2DS1 stained in both channels. Each symbol represents one cell. Horizontal bars and errors represent the medians and interquartile range. Data are from 27-30 cells from two (positive control) or three (KIR2DS1 vs. KIR2DL1) independent experiments. Representative 3 × 3 µm regions from STED images are shown in (**E**), scale bars 500 nm. *** $p < 0.001$, Kruskal-Wallis test with Dunn's post-test.

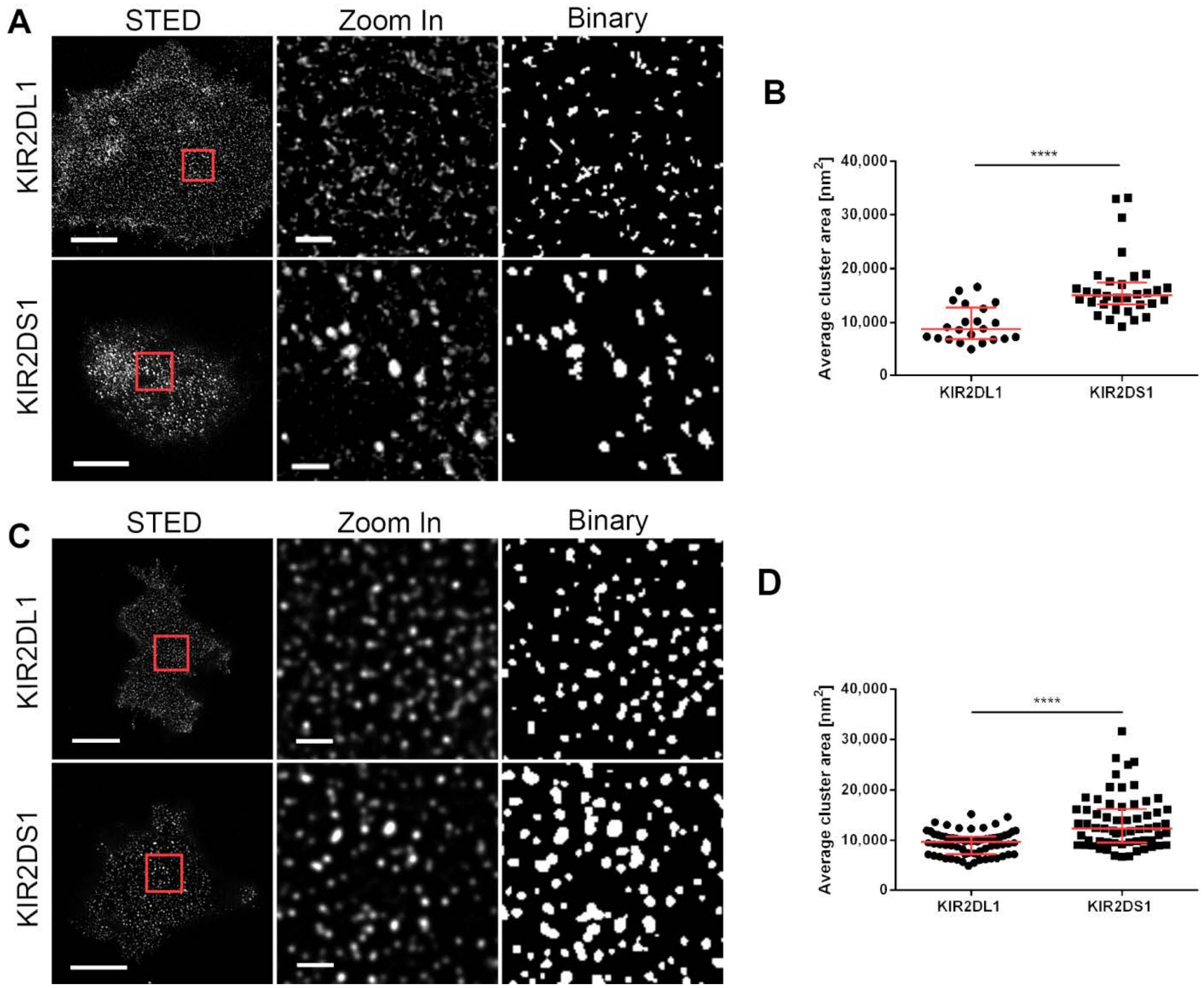

Figure S5. KIR2DL1 and KIR2DS1 are differentially organized at the surface of human NK cells as visualized by STED microscopy (referring to figure 1 and figure 2). NKL/KIR2DL1-HA and NKL/KIR2DS1-HA cells (**A-B**) as well as 2DS1$^-$/2DL1$^+$ and 2DS1$^+$/2DL1$^-$ NK clones (**C-D**) on PLL-coated slides were stained with anti-HA mAb conjugated to AF 488 (**A-B**) or anti-KIR2DL/S1 mAb conjugated to Atto 488 (**C-D**) and imaged by STED microscopy. (**A** and **C**) Representative STED images of KIR2DL1 and KIR2DS1 stained in NKL transfectants (**A**) and in primary NK clones (**C**). Scale bars 5 µm. The 3 × 3 µm regions outlined in red in STED images are zoomed-in and shown with corresponding binary maps (scale bars 500 nm). (**B**) Comparison of average cluster area between KIR2DL1 and KIR2DS1 in NKL/KIR2DL1-HA and NKL/KIR2DS1-HA cells, respectively. Data are from 22 (KIR2DS1) and 32 (KIR2DL1) cells from three independent experiments. (**D**) Comparison of average cluster area between KIR2DL1 and KIR2DS1 in 2DS1$^-$/2DL1$^+$ and 2DS1$^+$/2DL1$^-$ NK clones, respectively. Data are from 69 (KIR2DL1) and 62 (KIR2DS1) cells each from two clones derived from one donor. Each symbol represents the mean value from one cell. Horizontal bars and errors represent the medians and interquartile range. **** $p < 0.0001$, Mann-Whitney test.

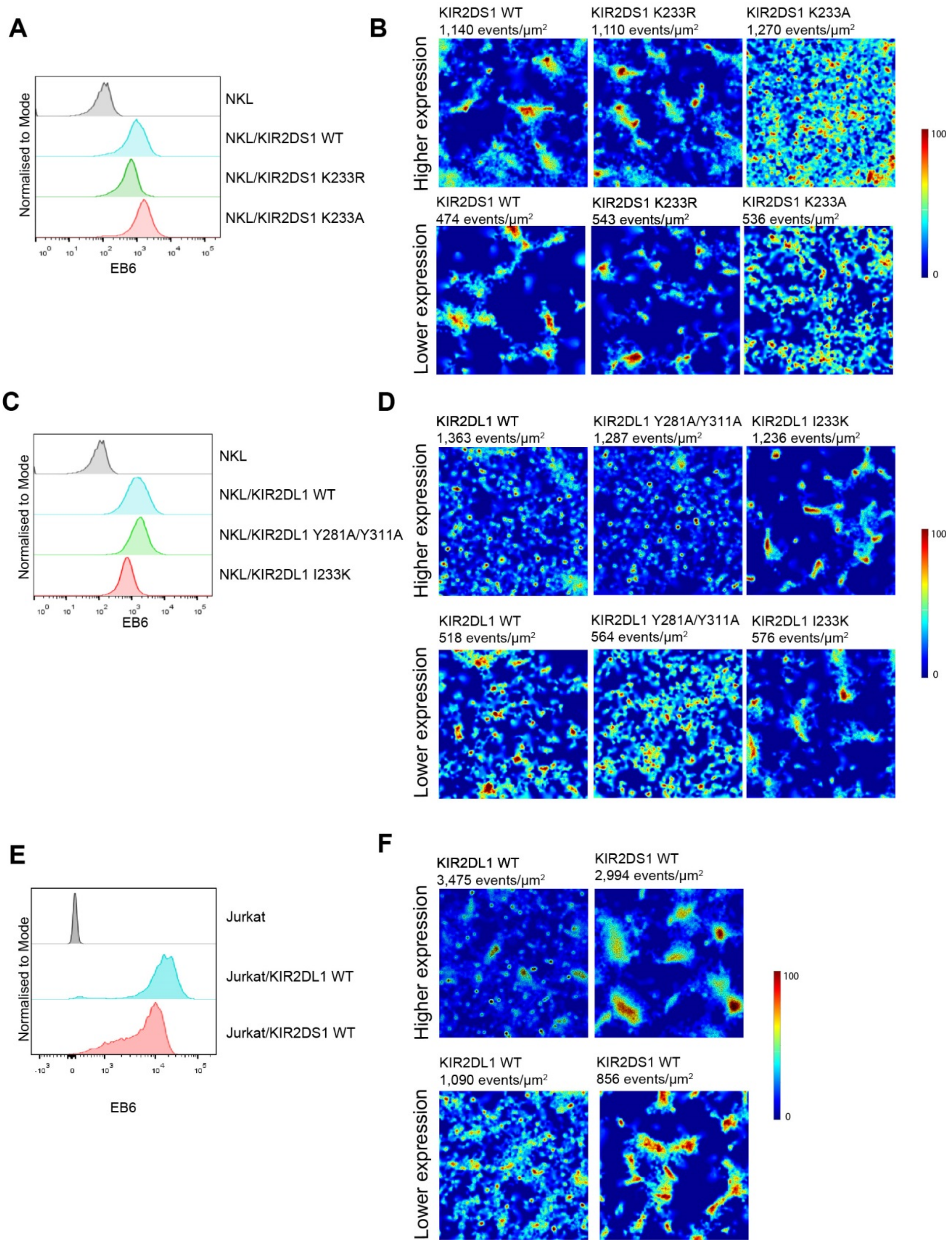

Figure S6. Differences in clustering of wild-type KIR2DS1 and KIR2DL1 and mutated receptors KIR2DS1^{K233A}, KIR2DS1^{K233R}, KIR2DL1^{I233K} and KIR2DL1$^{Y281A/Y311A}$ are not affected by relative density of detected molecules (referring to figure 3 and figure 4). (**A**) Parental NKL, NKL/KIR2DS1-HA, NKL/KIR2DS1^{K233A}, NKL/KIR2DS1^{K233R}, (**C**) Parental NKL, NKL/KIR2DL1-HA, NKL/KIR2DL1^{I233K} NKL/KIR2DL1$^{Y281A/Y311A}$, (**E**) Parental Jurkat, Jurkat/KIR2DL1-HA and Jurkat/KIR2DS1-HA cells were stained with anti-KIR2DL/S1 mAb (clone EB6) and analyzed by flow cytometry. (**B**, **D** and **F**) Wild-type and mutated forms of KIR2DS1 (**B**) and KIR2DL1 (**D**) in NKL and wild-type KIR2DL1 and KIR2DS1 in Jurkat (**F**) were stained with anti-HA mAb conjugated with AF488 and imaged by GSD microscopy. Selected 3 × 3 μm regions are shown as color maps where colors correspond to the extent of clustering according to pseudo-color scale. Regions from cells with relatively high (upper rows) and low (lower rows) densities of detected events are shown.

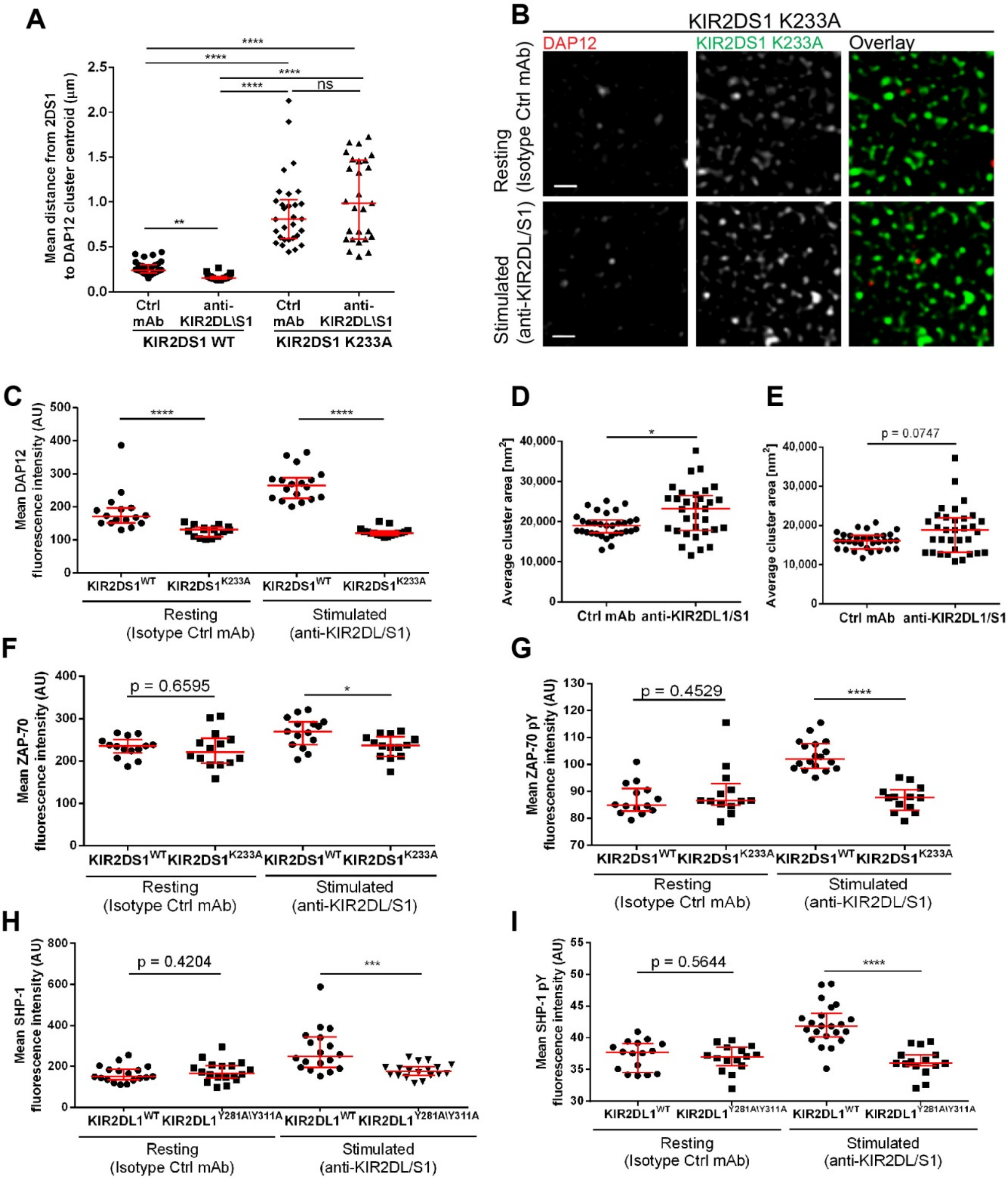

Figure S7. The effect of ligation of wild-type KIR2DL1 and KIR2DS1 and mutated receptors (referring to figure 5, figure 6 and figure 7). (A – E) NKL/KIR2DS1-HA or NKL/KIR2DS1^{K233A} cells were incubated on isotype control- or anti-KIR2DL/S1 mAb-coated slides, stained for KIR2DS1 (AF488) and DAP12 (AF568) and imaged by STED. (**A**) The average distances between centroids of KIR2DS1 clusters and a nearest cluster of DAP12. (**B**) Representative 3 × 3 µm regions from STED images of NKL/KIR2DS1^{K233A} cells with channels overlaid and separated. Scale bars 500 nm. (**C**) Mean DAP12-derived fluorescence intensity compared between NKL/KIR2DS1-HA and NKL/KIR2DS1^{K233A} cells on isotype control- and anti-KIR2DL/S1 mAb-coated slides. **(D – E)** Average area of KIR2DS1 (**D**) and DAP12 (**E**) clusters in NKL/KIR2DS1-HA cells on isotype control- or anti-KIR2DL/S1 mAb. (**F** and **G**) Mean ZAP-70- (**F**) and ZAP-70 pY319- (**G**) derived fluorescence intensity compared between NKL/KIR2DS1-HA and NKL/KIR2DS1^{K233A} cells on isotype control- and anti-KIR2DL/S1 mAb-coated slides. (**H** and **I**) Mean SHP-1- (**H**) and SHP-1 pY536- (**I**) derived fluorescence intensity compared between NKL/KIR2DL1-HA and NKL/KIR2DL1$^{Y281A/Y311A}$ cells on isotype control- and anti-KIR2DL/S1 mAb-coated slides. Each symbol represents one cell. Horizontal bars and errors represent the medians and interquartile range.

ns non-significant, * $p < 0.05$, ** $p < 0.01$, *** $p < 0.001$, **** $p < 0.0001$, Kruskal-Wallis test by ranks with Dunn's post-test (**A**) or Mann-Whitney test (**C** - **I**).

Supplemental Experimental Procedures

Plasmid generation.

The coding sequences of *KIR2DL1*002* and *KIR2DS1*00502* were ligated into a retroviral transfer vector pIB2 at the BamHI and EcoRI sites. HA and FLAG tags were then inserted at EcoRI site by non-directional ligation resulting in a glutamic acid, phenylalanine linker. Point mutations in KIR2DS1 and KIR2DL1 sequence were introduced by site-directed mutagenesis (Q5 Site-directed Mutagenesis Kit, New England Biolabs) using primers listed in Table S1.

Table S1. Primers used for site-directed mutagenesis (referring to figure 3 and figure 4).

**KIR2DS1 K233A**	Forward 5'CTCAGTGGTCGCCATCCCTTTCACC3'
	Reverse 5'GTCCCAATCAGAACATGTAG3'
**KIR2DS1 K233R**	Forward 5'CTCAGTGGTCCGGATCCCTTTCAC3'
	Reverse 5'GTCCCAATCAGAACATGTAG3'
**KIR2DL1 I233K**	Forward 5'CTCAGTGGTCAAGATCCTCTTCATC3'
	Reverse 5'GTCCCAATCAGAATGTGC3'
**KIR2DL1/Y281A/Y311A**	Forward 5'CCCTTCTCAGAGGCCCAAGACACCCCCAACAGATATCATCGTGGCCACGGAACTTCCAAATGCTG3'
	Reverse 5'CGAGTGATTTTTCTCTGTGTGAAAACGCAGTGATTCAACTGTGTGGCTGTCACCTCCTGAGGGTC3'

Retroviral transduction of NKL and Jurkat E6.1 cells.

The packaging cell line Phoenix-Amphotropic (Nolan Lab, Stanford) was transfected with Lipofectamine 3000 (Invitrogen) with a retroviral transfer vector pIB2 encoding KIR2DL1 or KIR2DS1 fused to HA tag or FLAG tag, or the same receptors in which single amino-acids were mutated. Viral supernatant collected 48 hours after transfection was used for three sequential centrifugations (300g, 45 min, 32°C) for infection of 10^6 cells. Cells expressing desired receptors were selected by addition of 10 µg/ml of blasticidin (InvivoGen) to the culture media.

NK clones generation.

Single NK cells were plated in individual wells of 96-well plates and cultured in clone medium (DMEM, 10% human serum, 15% F-12 HAMS, 1% penicillin and streptomycin, 2mM L-glutamine, 1mM sodium pyruvate, 1% MEM non-essential amino acids) in the presence of 10^8/ml irradiated (40 Gy) peripheral blood mononuclear cells (PBMCs) from two allogeneic individuals, 5×10^6/ml irradiated (40 Gy) RPMI 8866 cells, 400 U/ml of IL-2 and 5 ng/ml phytohemagglutinin for a week. On day 7, fresh clone media supplemented with 10^8/ml irradiated (40 Gy) PBMCs from two allogeneic individuals, 5×10^6/ml irradiated (40 Gy) RPMI 8866 cells and 400 U/ml of IL-2 were added. After a further seven days, cells were cultured in clone medium supplemented with 400 U/ml of IL-2 and passaged every 3 - 4 days.

Flow cytometry.

To assess surface expression of KIR2DL1-HA, KIR2DS1-HA and mutated forms of the receptors cells were washed in washing buffer (1% FBS/phosphate-buffered saline (PBS)), blocked with 1% AB human serum (Sigma)/washing buffer for 20 min at room temperature (RT) and stained with anti-KIR2DL/S1 mAb (clone EB6, Beckman Coulter) or isotype-matched control mAb (clone P3.6.2.8.1, eBioscience) conjugated with APC for 30 min at 4°C.

To assess expression of KIR2DL1-HA and KIR2DS1-FLAG or KIR2DL1-FLAG and KIR2DS1-HA in double transfectants, cells were fixed by resuspending in 4% PFA/PBS, incubated for 30 min incubation at RT, washed and blocked with 1% AB human serum/washing and permeabilizing buffer (1% FBS/0.1% saponin (Sigma)/PBS). Cells were then stained with 5 µg/ml of anti-HA mAb (clone 6E2, Cell Signaling) conjugated with AF647 and 5 µg/ml of anti-DYKDDDK Tag mAb (rabbit polyclonal, Cell Signaling) conjugated with AF488 or appropriate isotype-matched control Ab (mouse IgG1 isotype control, clone MOPC-21 from Biolegend and rabbit IgG isotype control, #4340 from Cell Signaling Technology) for 30 min at 4°C. Cells were then washed with washing buffer, fixed in 2% paraformaldehyde/PBS, assessed by BD FACS Canto II flow cytometer (BD Biosciences) and analyzed (FlowJo_V10 software).

Activation on slides assay.

Chambered glass coverslips (#1.5 Lab-Tek, Nunc) were coated with 0.01% poly-L-lysine (PLL, Sigma) and anti-KIR2DL/S1 (clone EB6) or anti-NKG2D (clone 149810; R&D Systems) mAb at 5 µg/ml plus 5 µg/ml of control murine IgG1 (clone MOPC-21, Biolegend), 10 µg/ml of IgG1 or 5 µg/ml of anti-KIR2DL1 plus 5µg/ml of anti-NKG2D mAb. Cell were allowed to settle on the slides for 10 min at 37°C and fixed in 4% PFA/PBS at RT for 30 min. Actin was visualized with AF488-labeled phalloidin (Invitrogen, 1/200 dilution) in 0.1% saponin/PBS and imaged by confocal microscopy (Leica TCS SP8 STED CW microscope). Images were exported to ImageJ and the percentage of cells forming peripheral actin rings was quantified.

Enzyme-linked immunosorbent assay (ELISA).

To assess the effect of KIR2DL1 ligation on the NKG2D-mediatd production of IFN-γ, parental NKL and NKL/KIR2DL1-HA cells were co-incubated with 721.221 target cells transfected to express MICA (a ligand for NKG2D) and HLA-Cw4 (a ligand for KIR2DL1 and KIR2DS1; 721.221/MICA/Cw4). To assess the effect of KIR2DS1 ligation on the production of IFN-γ, parental NKL and NKL/KIR2DS1-HA cells were co-incubated with 721.221 target cells transfected to express HLA-Cw4 only (721.221/Cw4). Cells were co-incubated with at an E:T ratio of 10:1 (200,000 NK cells and 20,000 target cells) for 24 hours. ELISA plates were coated with anti-IFN-γ mAb (clone NIB42, BD Biosciences, 1 µg/ml) in binding buffer (carbonate bicarbonate; Sigma) and blocked with 1% bovine serum albumin (BSA)/0.05% Tween-20/PBS. Supernatants were added in triplicate to the plate and incubated for 1 hour at RT. Plates were washed and incubated with biotinylated IFN-γ mAb (clone 4S.B3, BD Biosciences, 1 µg/ml) and then streptavidin HRP (BD Biosciences). The plates were developed with TMB ELISA substrate (Sigma-Aldrich); reaction was stopped with 1 N H_2SO_4. Absorbance was measured at 450 nm using a 570 nm reference line.

Sample preparation for imaging.

Chambered glass coverslips were coated with PLL and used for imaging of unstimulated cells or coated with 5 µg/ml anti-KIR2DL/S1 mAb (clone EB6) or murine IgG1 in PBS overnight at 4°C for stimulation of cells. Cells were allowed to settle on the slides for 10 min (resting on PLL) or 5 min (stimulating and isotype control-coated surfaces) at 37°C, fixed with 4% PFA/PBS for 30 min at RT and washed three times in PBS. Samples were blocked in 4% BSA/PBS for 40 min at RT. For immunostaining antibodies were diluted in 0.2% BSA/PBS at 5 µg/ml and where indicated cells were permeabilized with 0.1% saponin/0.2%BSA/PBS.

To visualize KIR2DL1 and/or KIR2DS1 cells were stained with following Ab: anti-KIR2DL/S1 (clone EB6) labeled in-house with Atto488 (Atto Tec), AF532 or AF647, anti-HA labeled with AF488 (clone 16B12, Invitrogen), anti-FLAG (clone M2; Sigma) conjugated in house with AF532 or rabbit anti-HA (C29F4, Cell Signaling Technology) followed by fluorescently labeled anti-rabbit secondary Ab (Invitrogen). The number of fluorophore molecules per antibody was 6 – 8. To visualize other proteins, the following primary Ab were used: anti-IL-2R alpha subunit (clone 7G7/B6, Millipore), anti-DAP12 (rabbit mAb, clone EPR5173, Abcam), anti-ZAP-70 (rabbit polyclonal, ab134509, Abcam) anti-ZAP-70 pY319 (rabbit polyclonal, ab131270 Abcam), anti-SHP-1 (rat mAb, clone 255402, R&D Systems), anti-SHP-1 pY536 (rabbit polyclonal, AP08023PU-N, Acris GmbH,); followed by fluorescently labeled secondary antibodies (Invitrogen). Staining was performed at RT for 60 min. Samples were then washed and imaged. GSD imaging was performed in 50 mM Tris-HCI (pH 8.0) with addition of 10 mM NaCl, 10% (w/v) Glucose, 1% (v/v) 2-mercaptoethanol, 0.7 mg/ml glucose oxidase and 42.5 µg/ml catalase.

Preparation of cells fixed in suspension.

To check for changes in membrane organization induced by contact with a glass surface we compared KIR2DL1 and KIR2DS1 clustering in NK cells fixed in suspension. For this, cells were suspended in 500 µl of 4% PFA/PBS and incubated for 30 min at RT, spun down, washed three times in PBS and blocked in 4% BSA/PBS for 20 min at RT. Cells were then incubated in 0.2% BSA/PBS in the presence of 5 µg/ml anti-KIR2DL/S1 mAb (clone EB6) conjugated with Atto 488, for 60 min at RT, washed 5 times with 1 ml of PBS, added to chambered slides pre-coated with PLL and allowed to settle for 5 min before imaging.

Microscopy

Single-molecule localization microscopy images were acquired on a Leica SR GSD microscope using an 160× oil immersion total internal reflection fluorescence (TIRF) objective (NA 1.43), and fluorescence was collected on an electron-multiplying charge-coupled device (EMCCD) camera (Andor iXon DU897E-C50-#BV). During acquisition, cells were illuminated in TIRF mode with 488 nm and 642 nm lasers. Up to 20,000 frames were acquired with a camera integration time of 15 ms.

STED images were acquired on Leica TCS SP8 STED CW microscope with a 100× oil immersion objective (1.40 NA). STED of AF488 and AF532 dyes was achieved using 592 nm continuous-wave fibre laser and for STED of AF568 a 660 nm continuous-wave fibre laser was used. Time-gated detection with a gate of 0.9‒6.0 ms was applied. To exclude cross-excitation of fluorophores in two-channel imaging, we acquired control images with only one of the two used laser lines (495 nm or 532/575 nm) active. To control for possible anti-Stokes excitation of AF532 by the 592 nm depletion laser, for each STED image of AF532 we took an additional image with only the depletion laser active and subtracted this image from its respective STED image. To ensure the specificity of the immuno-staining, all images were compared to images of respective cells stained with an isotype-matched Ab.

Image analysis.

For GSD image reconstruction using Thunderstorm software, each raw image frame was filtered using a wavelet transform (Izeddin et al., 2012) followed by calculation of approximate molecular positions from local intensity maxima. Sub-pixel localization of molecules was performed by fitting a symmetric two-dimensional Gaussian point-spread function models using a maximum likelihood estimation fitting method (Thompson et al., 2002). Poorly-localized molecular events were discarded by filtering events with intensity < 400 photons, sigma > 9 and uncertainty ≤ 50 nm. During the course of an image acquisition, the sample may undergo drift. Lateral drift was corrected using a cross-correlation method in Thunderstorm. To correct for multiple localizations originating from the same molecule, each event with subsequent localizations appearing nearby in space (within 30 nm) and time (using an off-gap of 25 frames) were combined and counted as one molecule. Merging parameters have to be selected to balance the desired merging of re-blinking events against inadvertently merging two truly discrete emitters that happen to be nearby (e.g. because they are in a real cluster). Here, parameters for merging events were selected by preparing a dilute solution of fluorophore-labeled antibody adsorbed onto glass and assessing the spatial and temporal scale over which re-blinking occurred when imaged by GSD microscopy. It remains possible that the merging parameters used here impose a systematic error in the precise counting of molecules and data are best interpreted comparatively. The table of event localizations was exported as a text file and raster images saved by rendering each localized event as a normalized Gaussian function. For quantitative analysis, between 1 and 5 non-overlapping 3 × 3 µm regions were selected for each cell from which data was then averaged, giving a representative result for that cell. Spatial pattern analysis using Ripley's *K* function was performed with SpPack (Perry, 2004), an add-in for Microsoft Excel.

Quantitative color-scale cluster maps based on univariate Getis and Franklin's local point pattern analysis method were generated using a custom MATLAB (Mathworks) with a sampling radius of 30 nm. The sampling radius of 30 nm for mapping clusters was chosen in an empirical way by analysing the same data set using several different radii and selecting the value which allowed clusters to be identified in the binary maps most accurately. Two-dimensional pseudo-color heat-maps were created by interpolating a surface plot with L(30) as the z-axis on a grid of resolution 5 nm. Binary maps were generated by overlaying a disc element of 25 nm radius around all point localizations with L(30) above a threshold value, L(30) ≥ 65. The threshold value was chosen by comparison of distribution of L(30) values between localizations in experimental regions and randomized regions of the same area and number of molecules. Number of clusters, cluster size and other cluster parameters were measured from the binary cluster maps using ImageJ.

Signaling molecules-derived fluorescence intensity was measured in raw STED images. For other measurements, STED images were deconvolved in Huygens Professional 10.1 software (Scientific Volume Imaging) using CMLE algorithm. For nanocluster measurements, STED images were converted into binary maps by applying an automatic triangle threshold using ImageJ to regions-of-interest containing a single cell. To avoid false detections caused by noise fluctuations or unbound labeled antibodies, objects with an area smaller than 1,000 nm^2 were excluded from further analysis. Analogical binary maps of clusters identified in two-channel STED images were used for calculations of distances between KIR2DS1 and DAP12 cluster centroids using a custom MATLAB script.

To assess protein co-localization we calculated thresholded Pearson's correlation coefficients in deconvolved multi-channel STED images using an ImageJ plug-in Coloc 2 (written by Daniel J White, Tom Kazimiers and Johannes Schindelin; available on-line), which employs the Costes auto-threshold.

To identify clusters of signaling molecules overlapping or in contact with KIR clusters and residing within their close proximity (i.e. partially overlapping or remaining in contact at the edges) we converted STED images into binary maps as described above and identified x-y coordinates of cluster-centroids using the particle analysis function in ImageJ. The area and integrated fluorescence intensity of each detected cluster were also measured.

A custom MATLAB script was used to search a circular area around the centroid of each KIR2DL/S1 cluster for the presence of DAP12/SHP-1/ZAP-70 clusters. For each KIR cluster, the search radius was the sum of that cluster's own radius (calculated from the cluster area and assuming circularity) and the 75th-percentile value for cluster radii of the cognate signaling molecule (for DAP12 78 nm in unstimulated and 80 nm in activated cells; for total SHP-1 122 nm in 103 unstimulated and 114 nm in activated cells; for pY536 SHP-1 84 nm; for total ZAP-70 99 nm in resting and 126 nm in activated cells; for pY319 ZAP-70 90 nm). The distribution of those KIR2DL/S1 cluster areas with at least one signaling molecule cluster nearby was compared against the distribution of all KIR cluster areas by dividing them according to their size into the three bins. The bins corresponded to tertiles, i.e. each bin contained one third of the total number of KIR clusters detected in a particular cell.

To assess whether covering a larger area by bigger clusters could account for these clusters forming more contacts with signaling molecules, data were compared against randomised equivalents. Here, the cluster segmentation data (comprising a list of cluster descriptors including centroid coordinates and the pixels comprising each segmented cluster) for the signaling molecule channel image were randomly assigned new centroid coordinates within a region of interest delineating the cell boundary. The pixel intensity values for each cluster were also repositioned about the new centroid location and the analysis (using the original cluster segmentation data for the KIR channel) was performed. In this way, signaling molecule clusters were disconnected from their original spatial locations (while retaining their original shape and intensity properties) and a measurement of a randomised, spatially unrelated co-clustering of KIR and signaling molecules could be obtained.

To assess relative efficiency of signaling proteins recruitment and phosphorylation within clusters of certain sizes, the median integrated fluorescence intensity of DAP12/SHP-1/ZAP-70 clusters was plotted against area bins of KIR2DL/S1 clusters. Only KIR2DL/S1 clusters in contact with at least one cluster of a respective molecule were included in this analysis.

Supplemental References

Izeddin, I., Boulanger, J., Racine, V., Specht, C. G., Kechkar, A., Nair, D., Triller, A., Choquet, D., Dahan, M. & Sibarita, J. B. 2012. Wavelet analysis for single molecule localization microscopy. *Opt Express,* 20**,** 2081-95.

Perry, G. L. W. 2004. SpPack: spatial point pattern analysis in Excel using Visual Basic for Applications (VBA). *Environmental Modelling & Software,* 19**,** 559-569.

Thompson, R. E., Larson, D. R. & Webb, W. W. 2002. Precise nanometer localization analysis for individual fluorescent probes. *Biophys J,* 82**,** 2775-83.
